# Supplementary material for: Circadian oscillation in primary cilium length by clock genes regulates fibroblast cell migration
Source: EMBO Rep. 2023 Nov 16;24(12):e56870. doi: 10.15252/embr.202356870 (PMC10702818; doi:10.15252/embr.202356870)
Supplement: Supplementary file 1 — Expanded View Figures PDF [file EMBR-24-e56870-s013.pdf]

Expanded View Figures

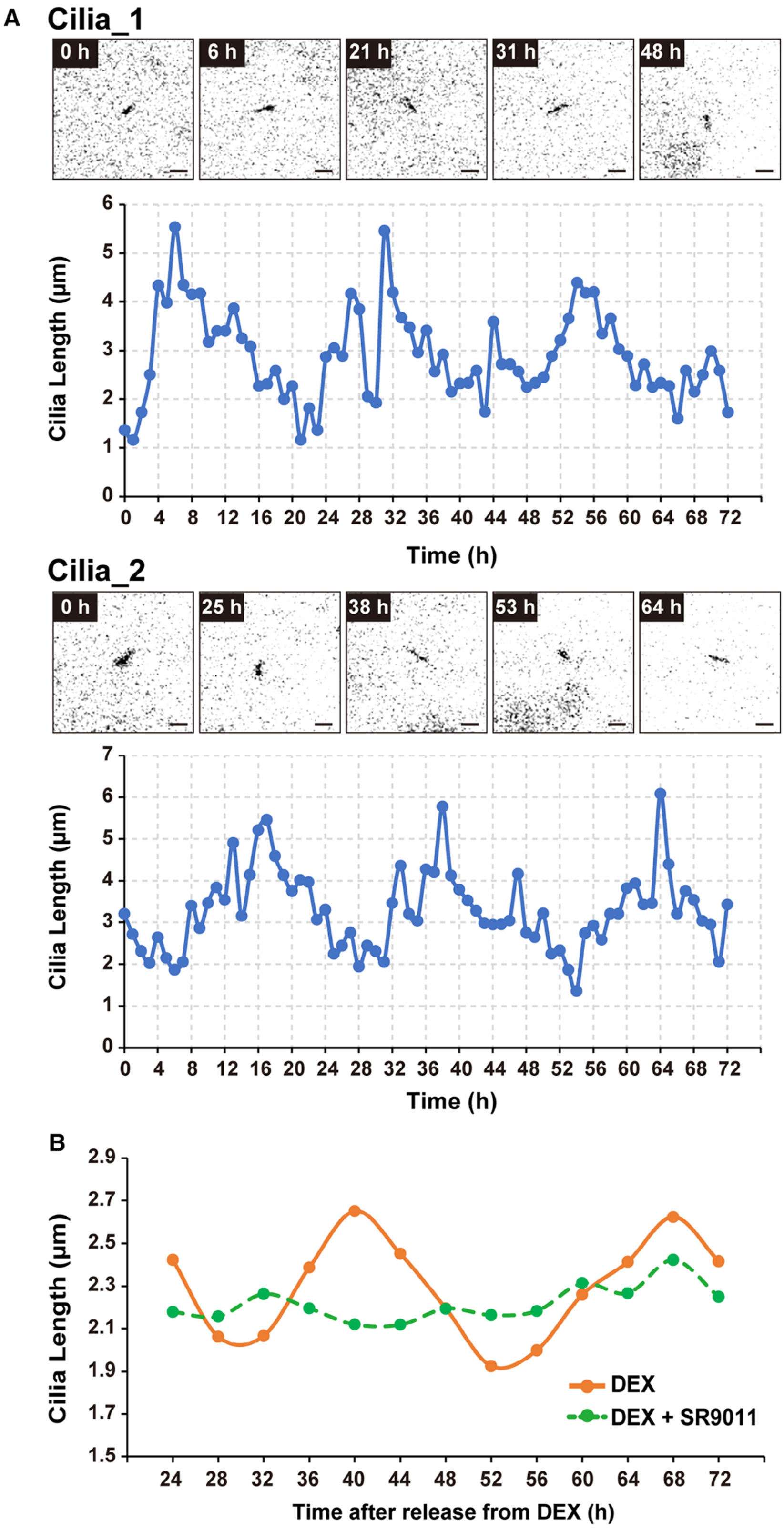

Figure EV1. Circadian rhythm of primary cilium length is continuous for more than 48 h.

A Representative time-lapse images of Arl13b-venus-expressing NIH/3T3 cells at the indicated time and quantitative analysis of primary cilium length from Movies EV3 and EV4. Scale bar, 2 μm.

B Quantitative analysis of primary cilium length in NIH/3T3 cells treated with 2 μM SR9011 at each indicated time point after release from DEX.

Source data are available online for this figure.

 © 2023 The Authors

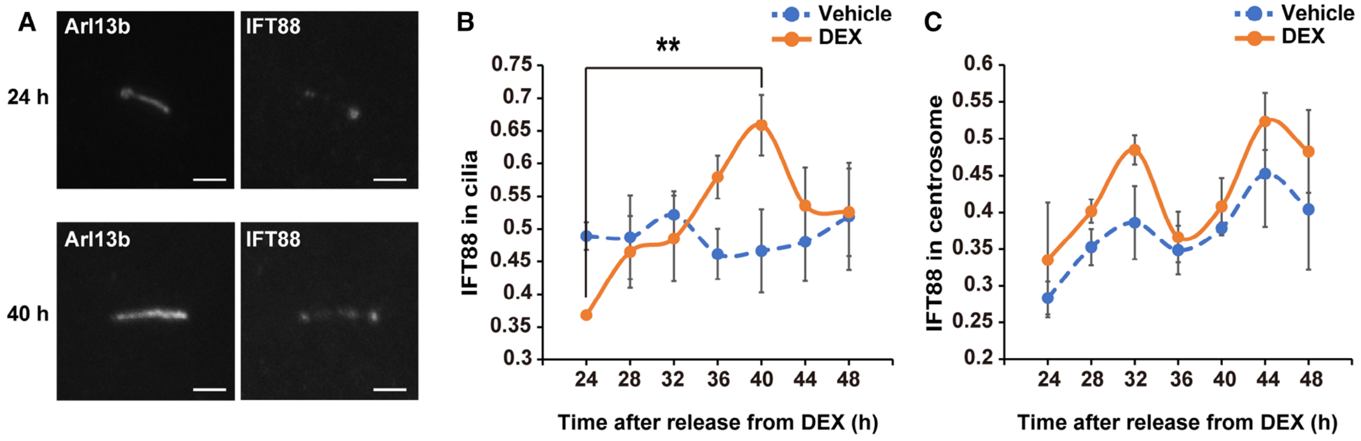

Figure EV2. Rhythmic dynamics of IFT88 in primary cilia.

A Immunostaining for primary cilia (Arl13b) and IFT88 in NIH/3T3 cells at 24 or 40 h after release from DEX. Scale bar, 2 μm.

B, C Quantitative analysis of fluorescence intensity of IFT88 in primary cilia (Arl13b, B) and centrosome (γ-tubulin, C) of NIH/3T3 cells at the indicated time point after release from DEX. The data are from three independent experiments.

Data information: Data in panels B and C are presented as mean ± SEM. $^{**}P \leq 0.01$ (one-way ANOVA).
Source data are available online for this figure.

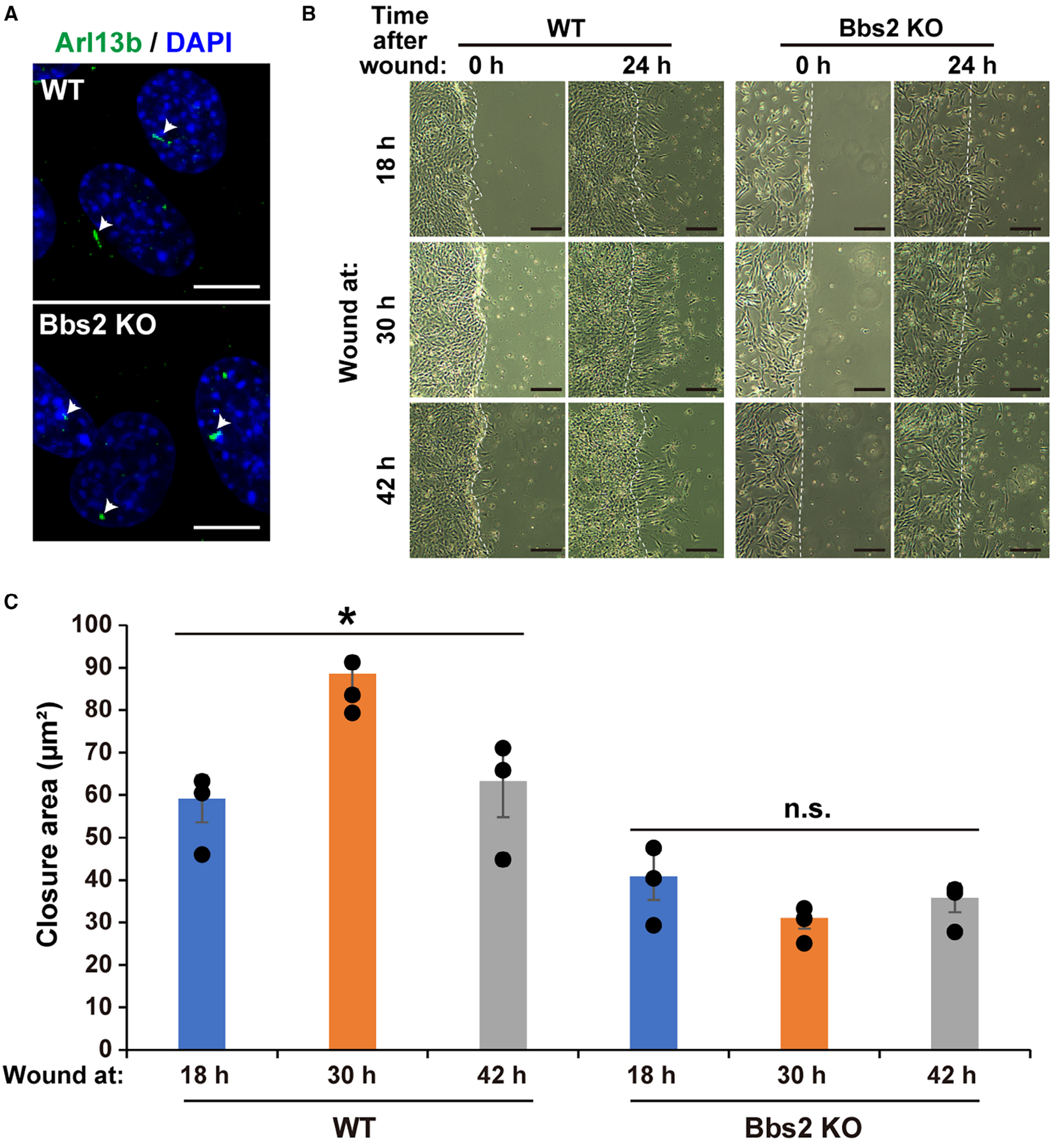

Figure EV3. Bbs2 knockout NIH/3T3 cells in wound healing.

A Immunostaining for primary cilia (red; Arl13B) and DNA (blue; DAPI) in WT or Bbs2-KO NIH/3T3 cells. Scale bar, 10 μm. White arrows point to primary cilia.

B Representative images of WT or Bbs2-KO NIH/3T3 cells at 0 or 24 h after wounding in the wound healing assay with the protocol illustrated in Fig 7 panel A. Scale bar, 200 μm.

C Quantitative analysis of the area of wound healing from panel B at 24 h after wounding. The data are from three independent experiments.

Data information: Data in panel C are presented as mean ± SEM. **P* ≤ 0.05; n.s. indicates no significant difference (one-way ANOVA).
Source data are available online for this figure.

 © 2023 The Authors

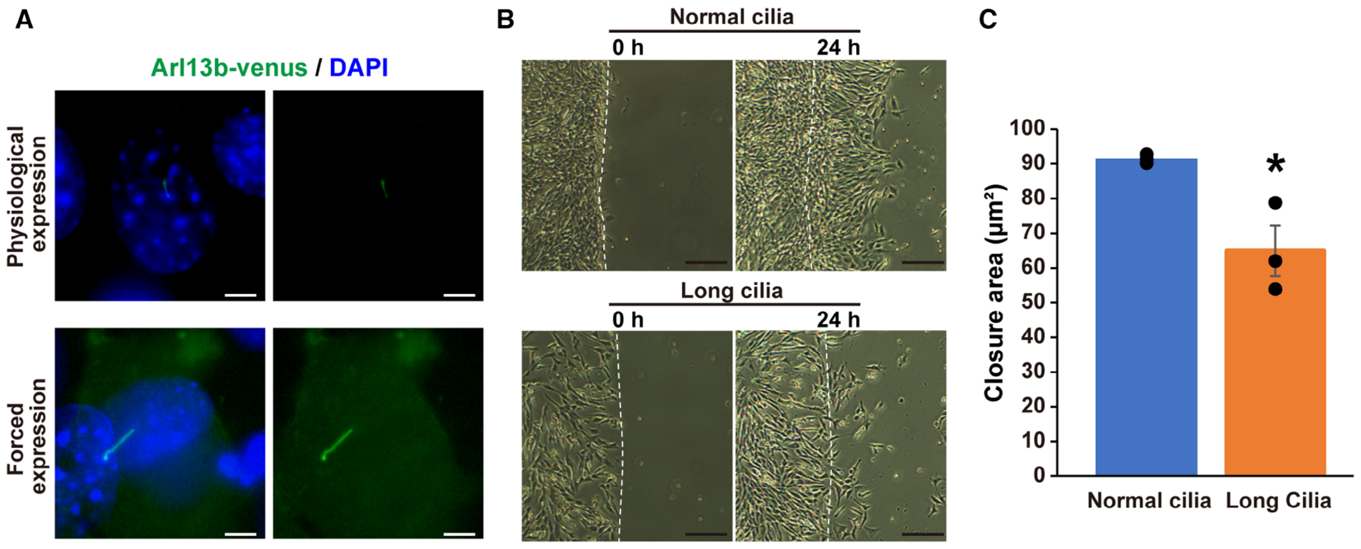

Figure EV4. Increased primary cilium length decreases cell migration.

A Immunostaining for GFP in NIH/3T3 cells with forced expression of Arl13b-venus (long cilia) or physiological expression of Arl13b-venus (normal cilia) at 0 or 24 h after wounding in the wound healing assay. Scale bar, 5 μm.

B Representative images of NIH/3T3 cells with long cilia or normal cilia at 0 or 24 h after wounding in the wound healing assay. Scale bar, 200 μm.

C Quantitative analysis of the area of wound healing from panel B at 24 h after wounding. The data are from three independent experiments.

Data information: Data in panel C are presented as mean ± SEM. $^*P \leq 0.05$ (one-way ANOVA).
Source data are available online for this figure.

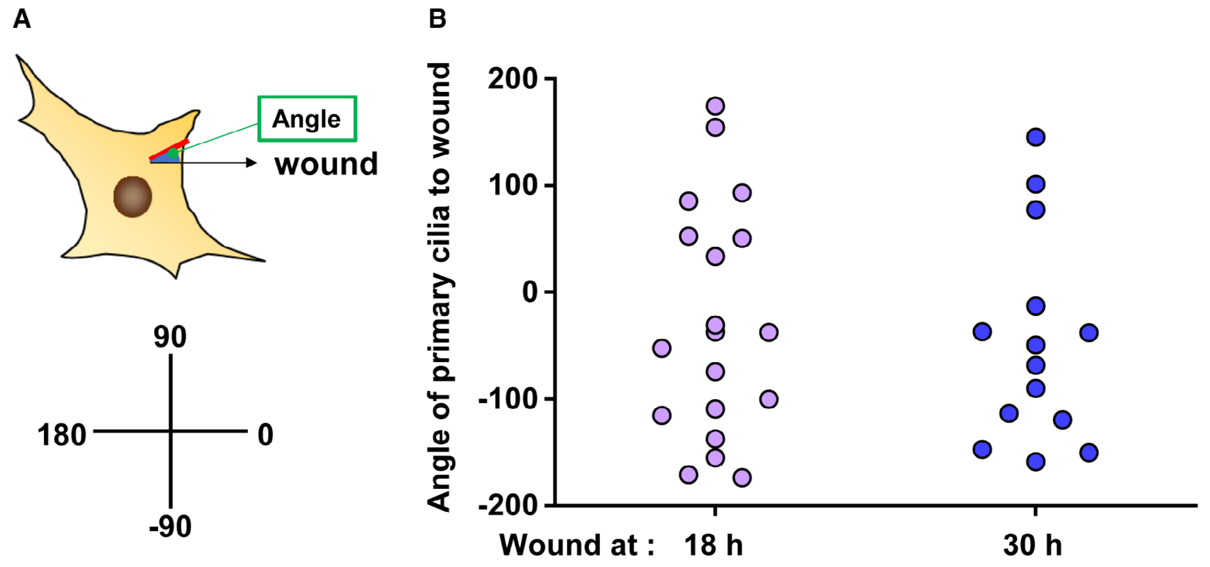

Figure EV5. Primary cilia angle in wound healing.

A Schematic diagram of the angle of primary cilia relative to the direction of the wound.

B Quantitative analysis of the angle of primary cilia from panel E in Fig 8. The data are from 19 cells at 18 h and 14 cells at 30 h.

Source data are available online for this figure.

© 2023 The Authors
