# Supplementary material for: Circadian oscillation in primary cilium length by clock genes regulates fibroblast cell migration
Source: EMBO Rep. 2023 Nov 16;24(12):e56870. doi: 10.15252/embr.202356870 (PMC10702818; doi:10.15252/embr.202356870)
Supplement: Supplementary file 2 — Movie EV1 [file EMBR-24-e56870-s011.zip › Movie EV1/Movie EV1 figure legend.docx]

**Movie EV1-Primary cilia influence fibroblast mobilization with circadian changes in wound healing (relates to Fig. 7D).**

This movie shown NIH/3T3 WT wounded at 30-hours after DEX synchronization in wound healing assay.
