# Supplementary material for: Circadian oscillation in primary cilium length by clock genes regulates fibroblast cell migration
Source: EMBO Rep. 2023 Nov 16;24(12):e56870. doi: 10.15252/embr.202356870 (PMC10702818; doi:10.15252/embr.202356870)
Supplement: Supplementary file 4 — Movie EV3 [file EMBR-24-e56870-s003.zip › Movie EV3/Movie EV3 figure legend_V2.docx]

**Movie EV3- Circadian rhythm of primary cilium length is continuous for 72 hours (relates to Fig. EV1A Cilia_1).**

This movie shown an Arl13b-venus expressing NIH/3T3 cells. Timestamp (hr:min) indicates the time since the start of the time-lapse imaging.
