# Supplementary figures and images for: Circadian oscillation in primary cilium length by clock genes regulates fibroblast cell migration (part 1 of 2)
Source: EMBO Rep. 2023 Nov 16;24(12):e56870. doi: 10.15252/embr.202356870 (PMC10702818; doi:10.15252/embr.202356870)

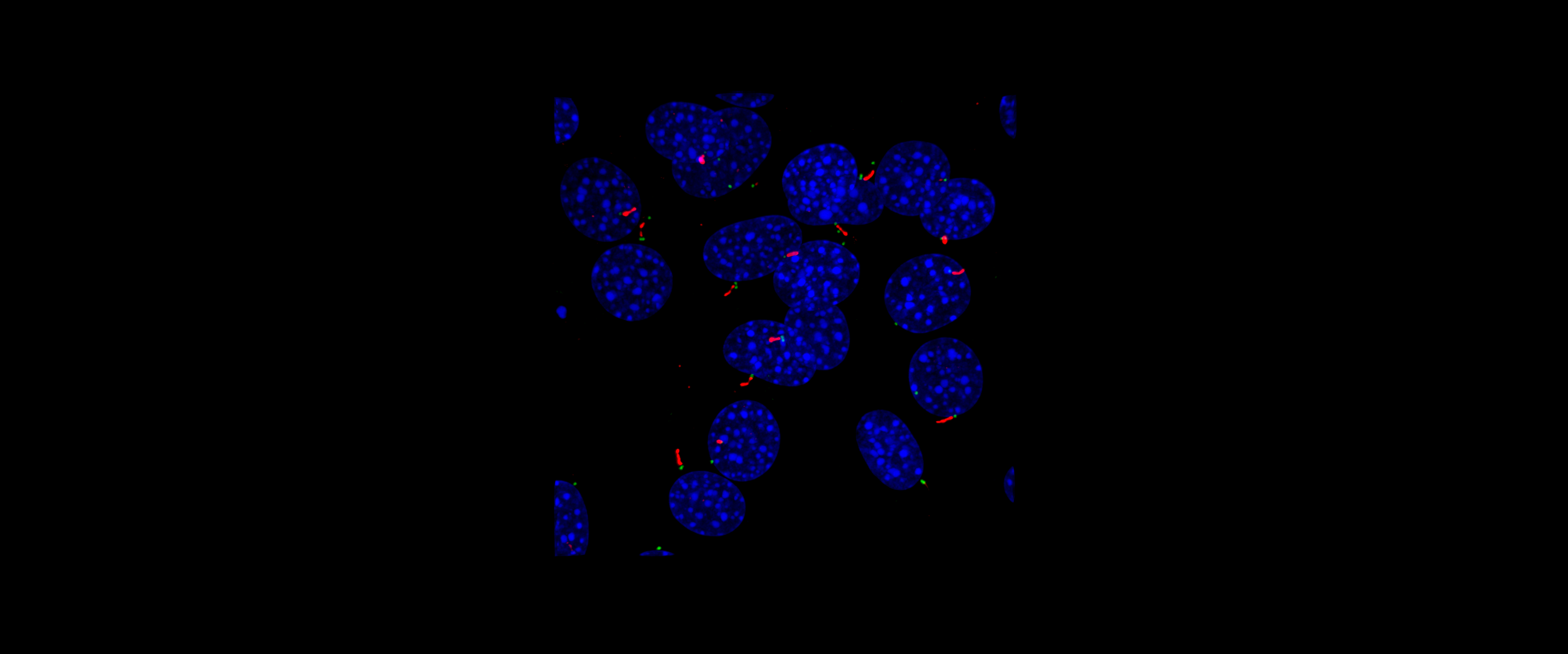

Supplement: Supplementary file 8 — Source Data for Figure 1 [file EMBR-24-e56870-s006.zip › Figure 1/1C/DEX/24 h.tif]

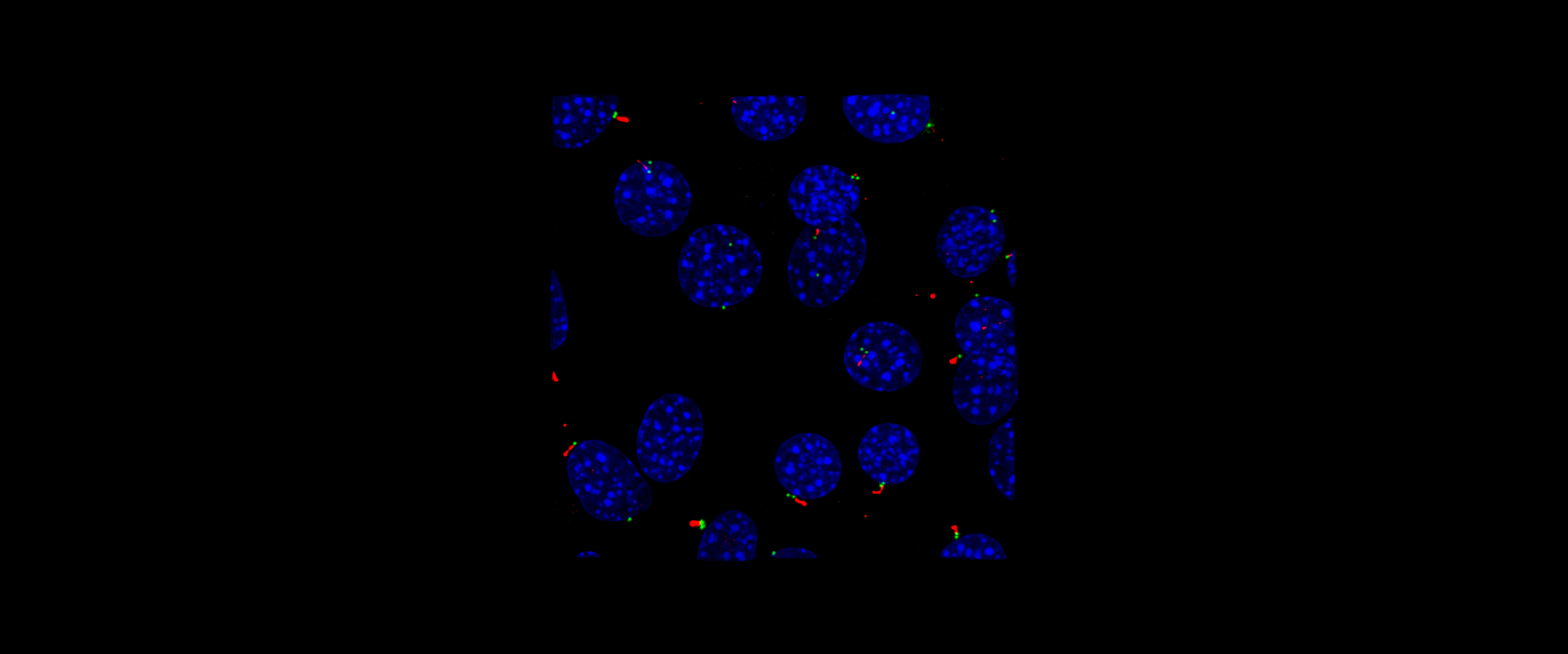

Supplement: Supplementary file 8 — Source Data for Figure 1 [file EMBR-24-e56870-s006.zip › Figure 1/1C/DEX/28 h.tif]

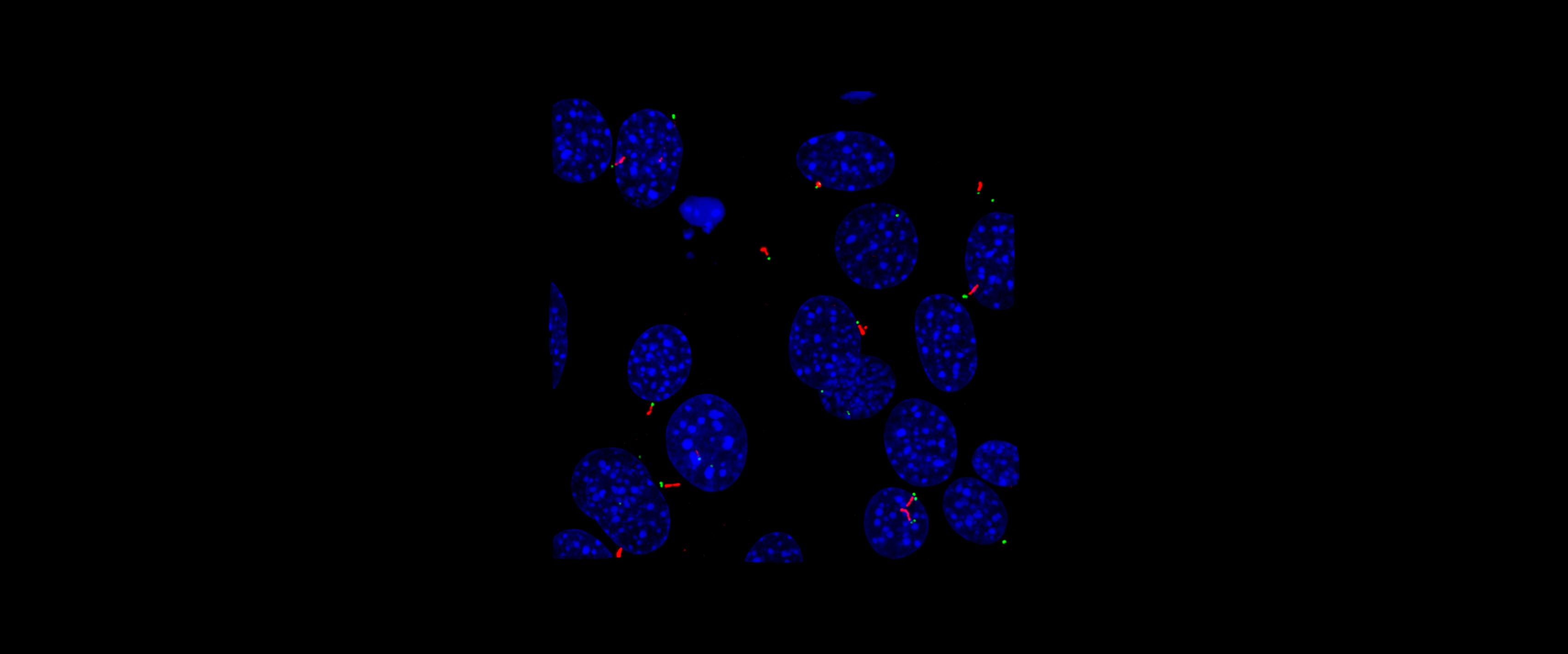

Supplement: Supplementary file 8 — Source Data for Figure 1 [file EMBR-24-e56870-s006.zip › Figure 1/1C/DEX/32 h.tif]

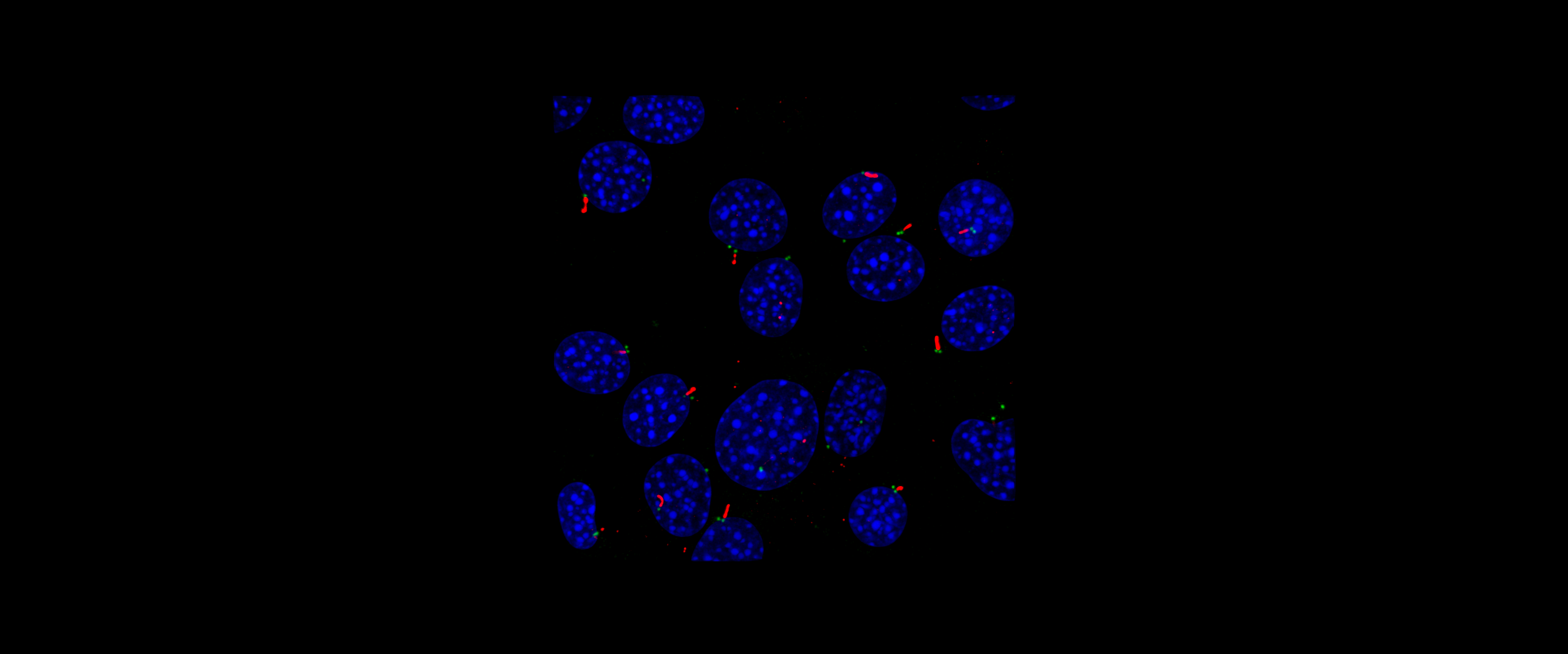

Supplement: Supplementary file 8 — Source Data for Figure 1 [file EMBR-24-e56870-s006.zip › Figure 1/1C/DEX/36 h.tif]

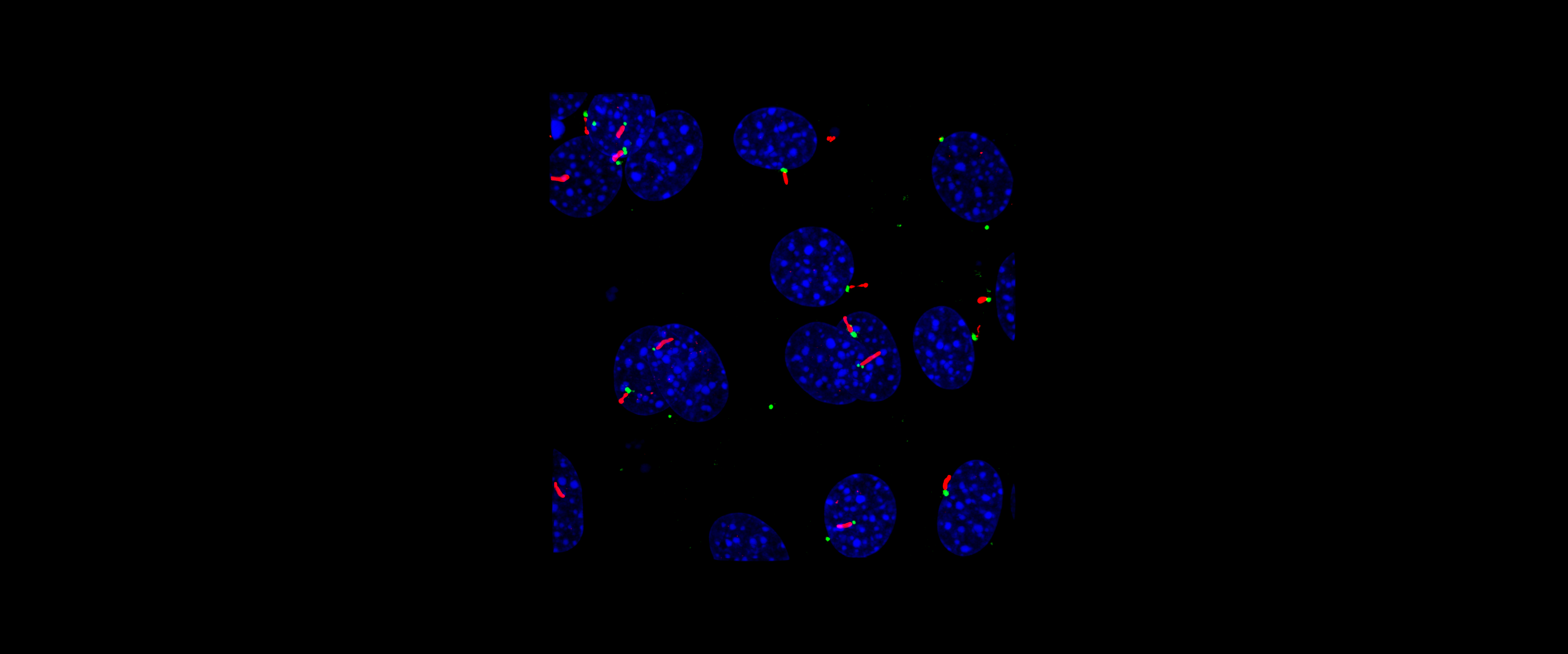

Supplement: Supplementary file 8 — Source Data for Figure 1 [file EMBR-24-e56870-s006.zip › Figure 1/1C/DEX/40 h.tif]

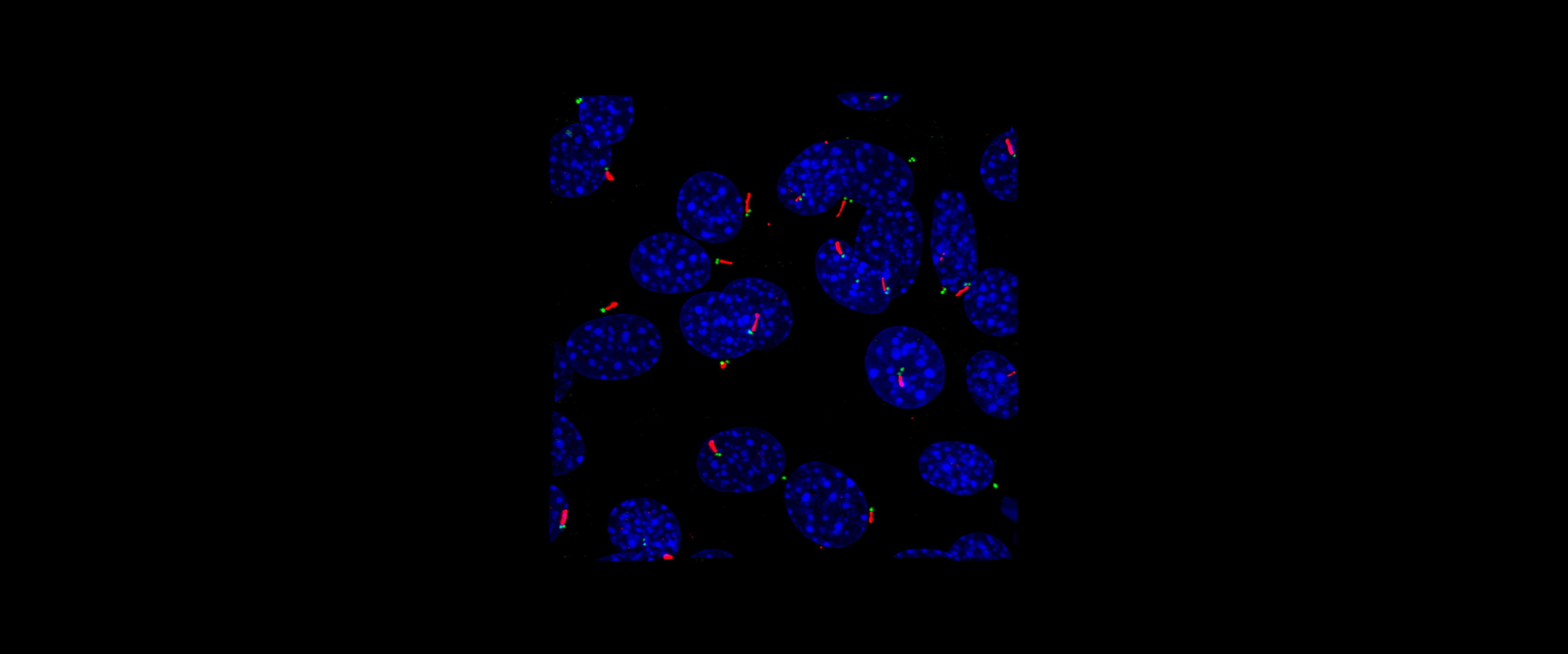

Supplement: Supplementary file 8 — Source Data for Figure 1 [file EMBR-24-e56870-s006.zip › Figure 1/1C/DEX/44 h.tif]

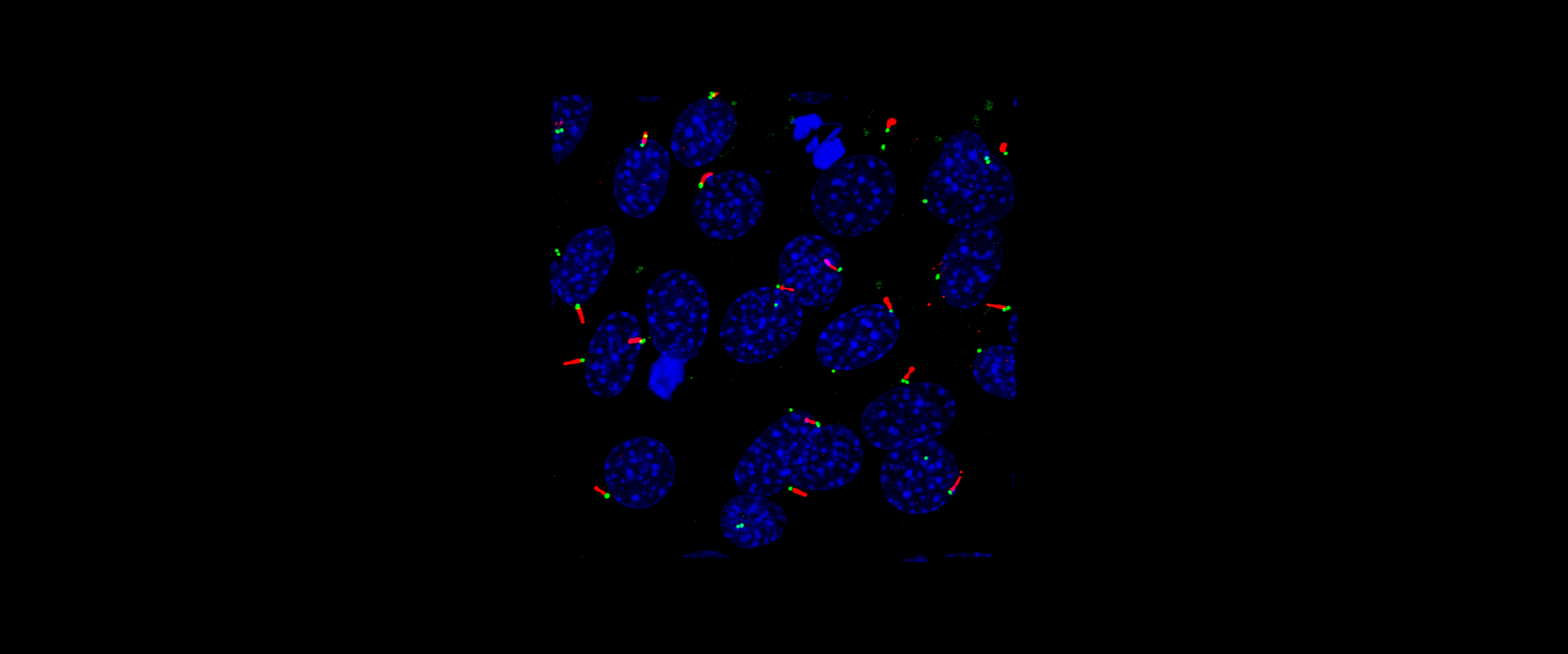

Supplement: Supplementary file 8 — Source Data for Figure 1 [file EMBR-24-e56870-s006.zip › Figure 1/1C/DEX/48 h.tif]

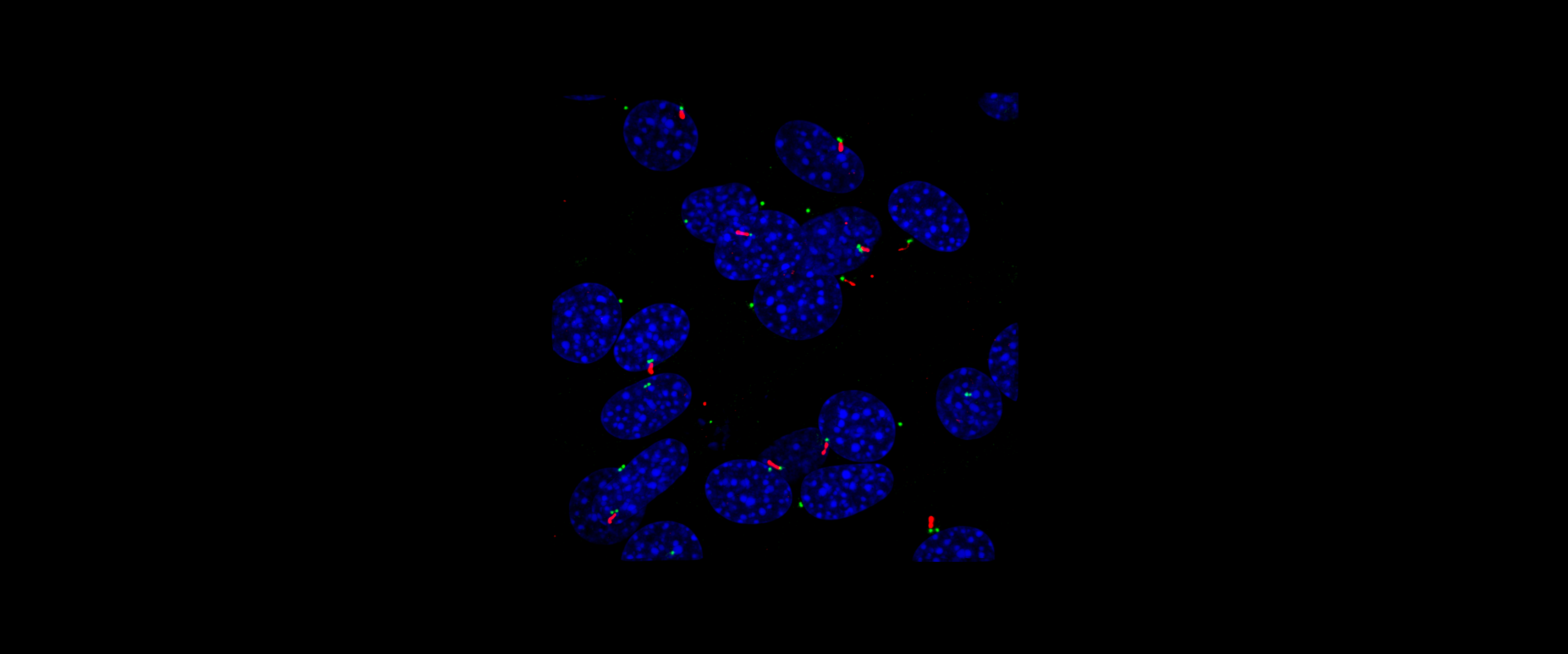

Supplement: Supplementary file 8 — Source Data for Figure 1 [file EMBR-24-e56870-s006.zip › Figure 1/1C/Vehicle/24 h.tif]

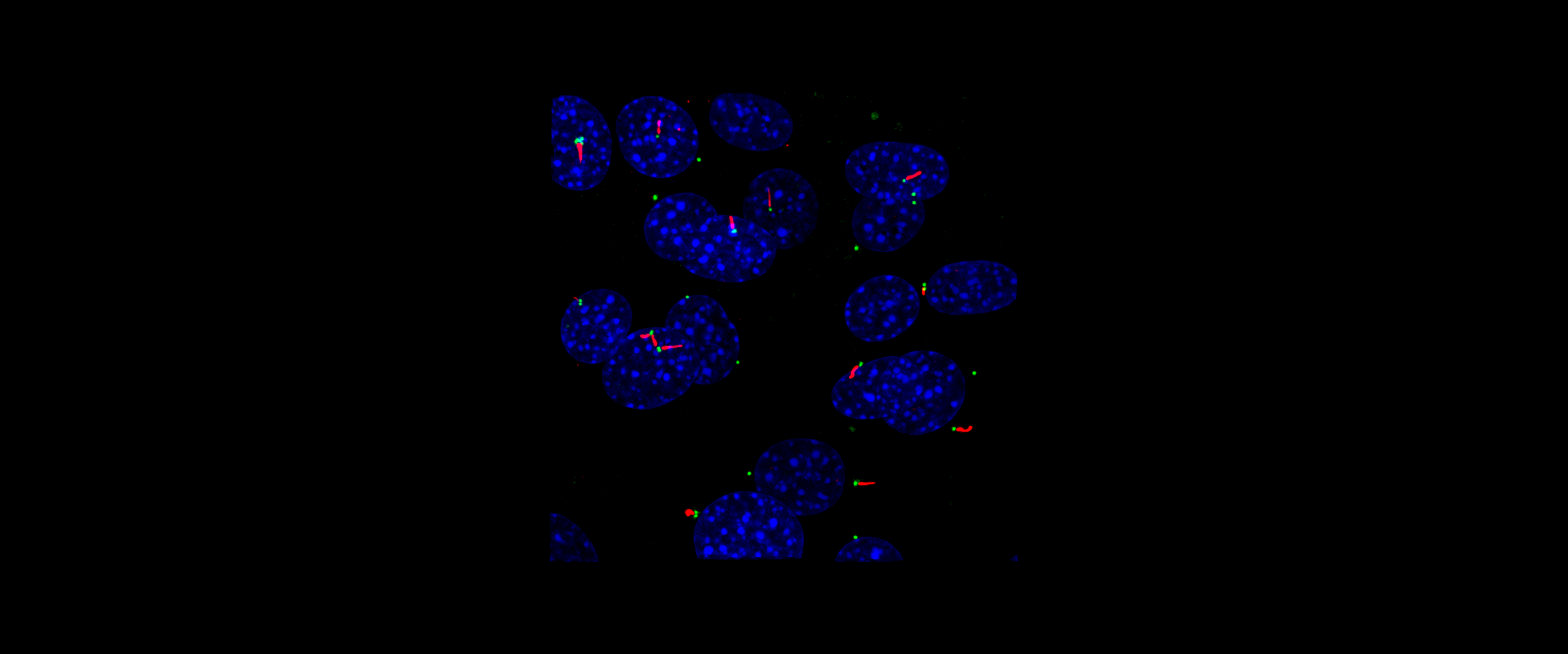

Supplement: Supplementary file 8 — Source Data for Figure 1 [file EMBR-24-e56870-s006.zip › Figure 1/1C/Vehicle/28 h.tif]

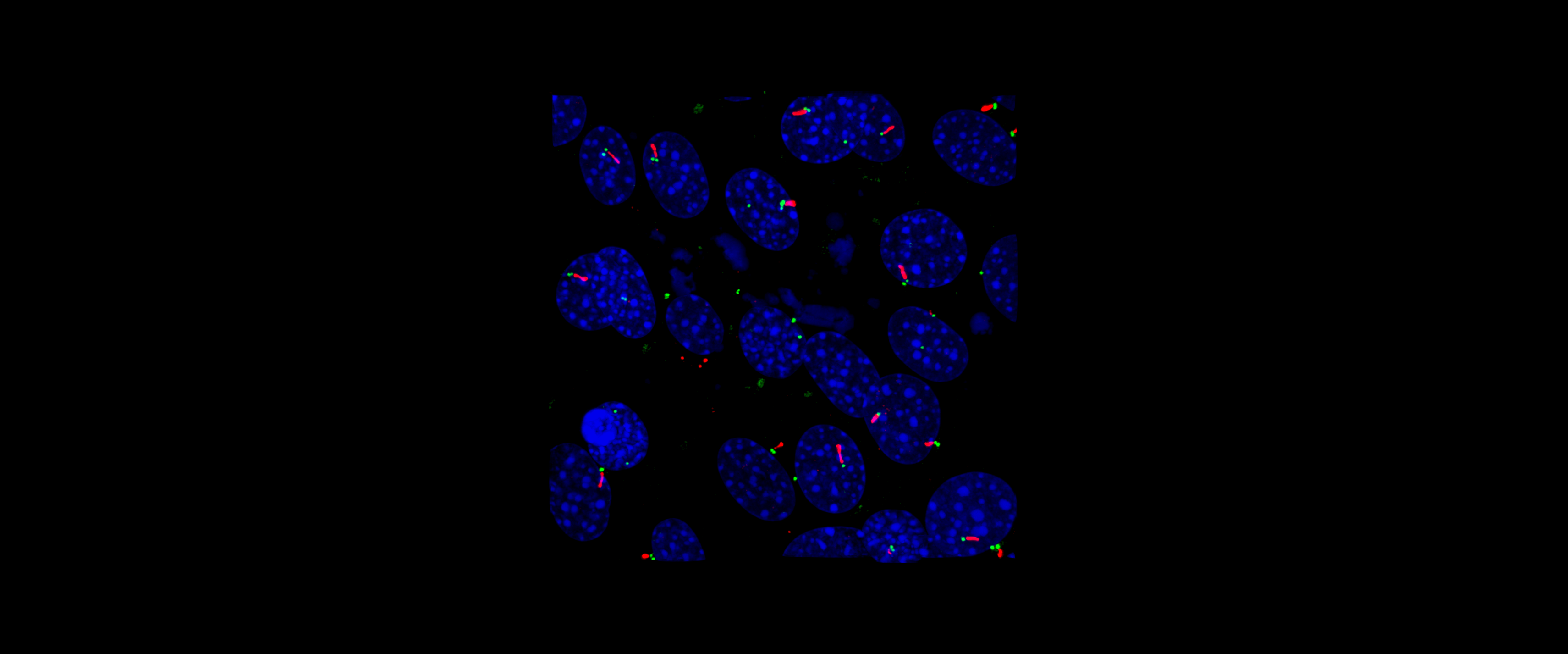

Supplement: Supplementary file 8 — Source Data for Figure 1 [file EMBR-24-e56870-s006.zip › Figure 1/1C/Vehicle/32 h.tif]

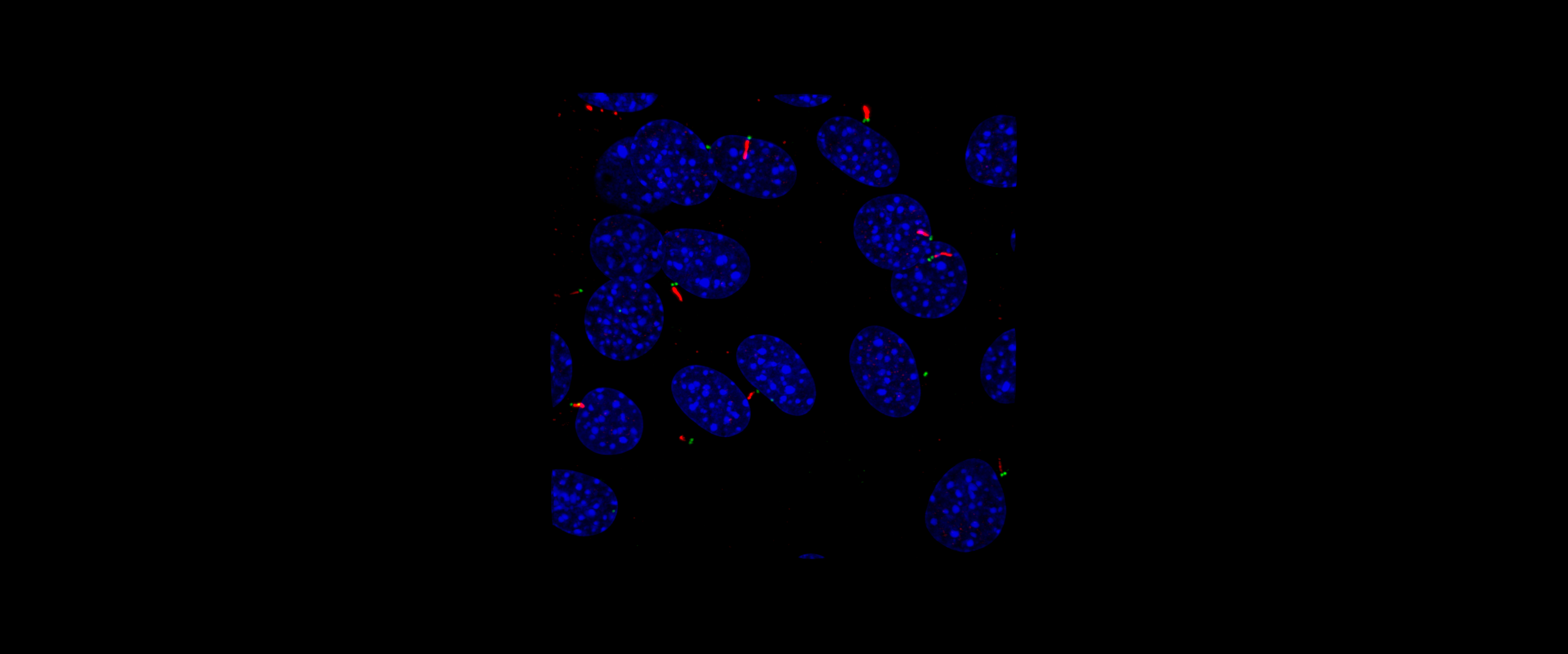

Supplement: Supplementary file 8 — Source Data for Figure 1 [file EMBR-24-e56870-s006.zip › Figure 1/1C/Vehicle/36 h.tif]

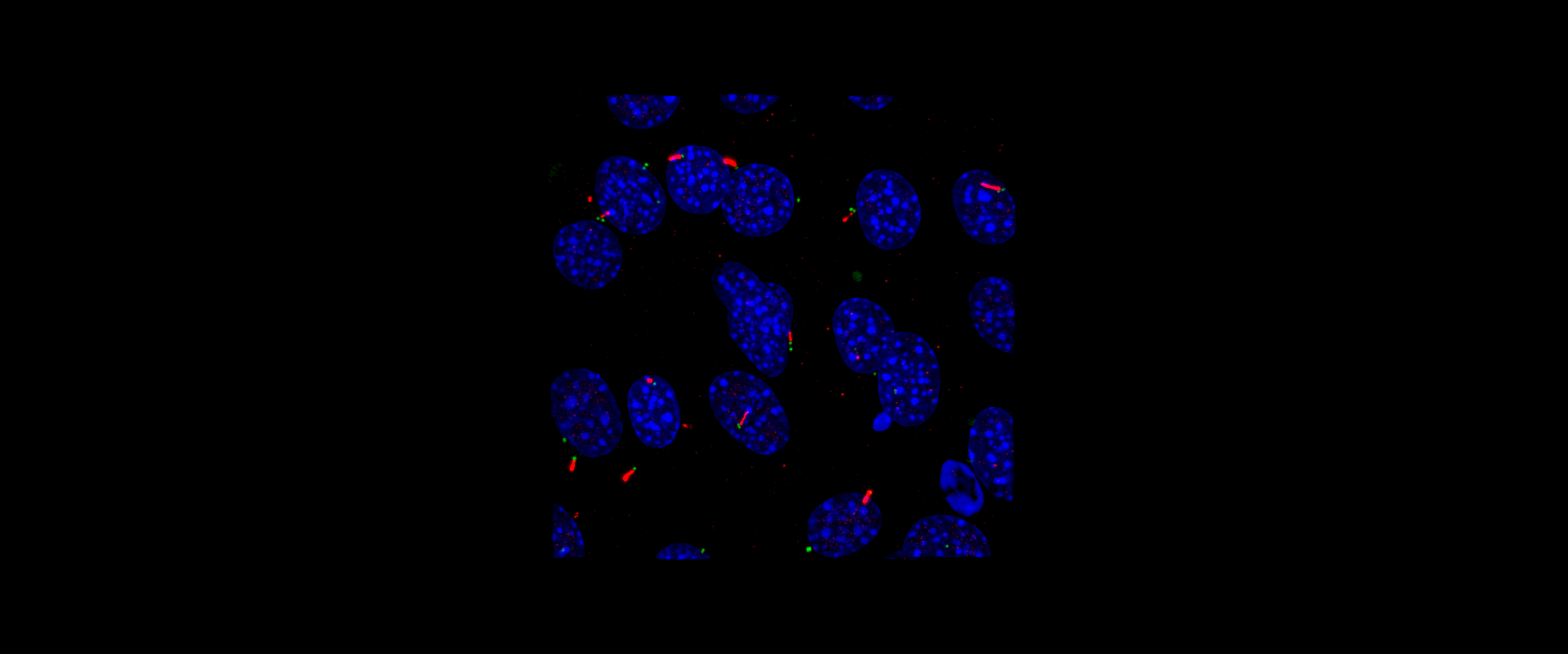

Supplement: Supplementary file 8 — Source Data for Figure 1 [file EMBR-24-e56870-s006.zip › Figure 1/1C/Vehicle/40 h.tif]

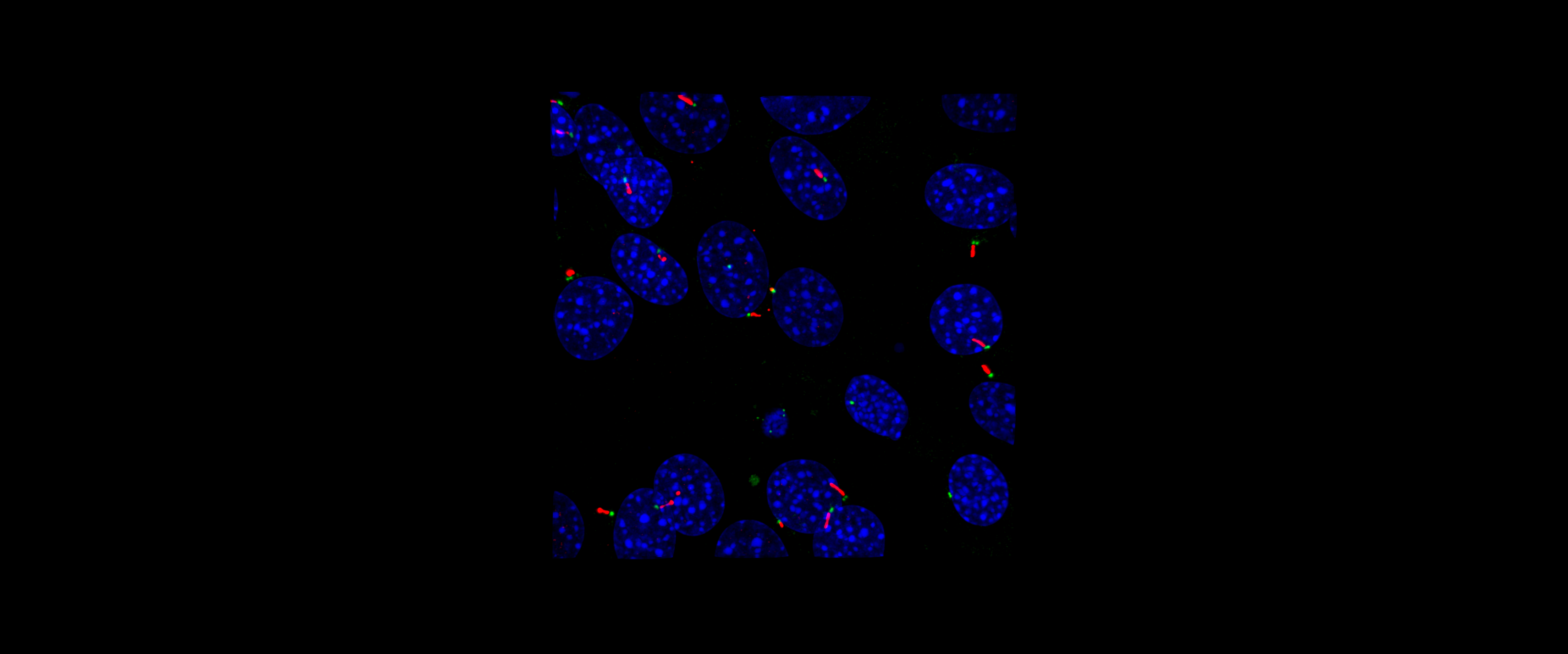

Supplement: Supplementary file 8 — Source Data for Figure 1 [file EMBR-24-e56870-s006.zip › Figure 1/1C/Vehicle/44 h.tif]

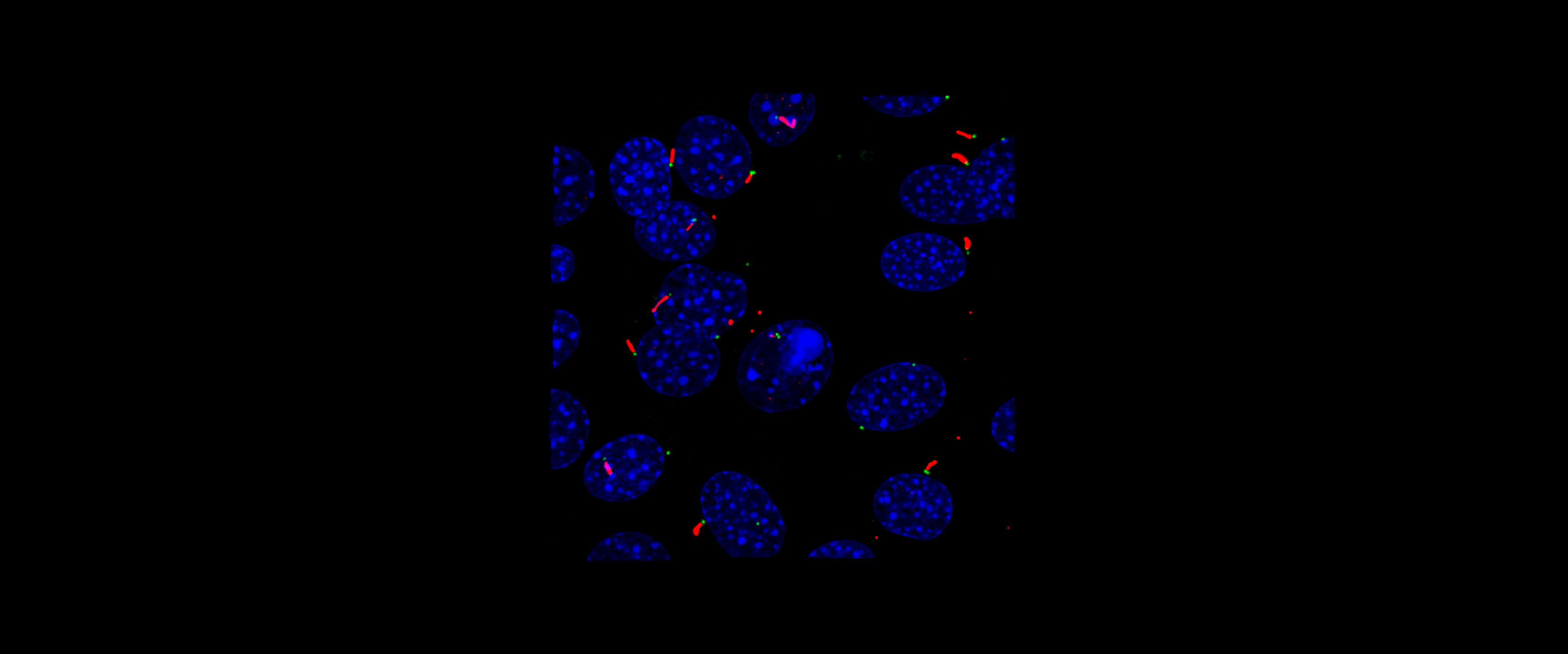

Supplement: Supplementary file 8 — Source Data for Figure 1 [file EMBR-24-e56870-s006.zip › Figure 1/1C/Vehicle/48 h.tif]

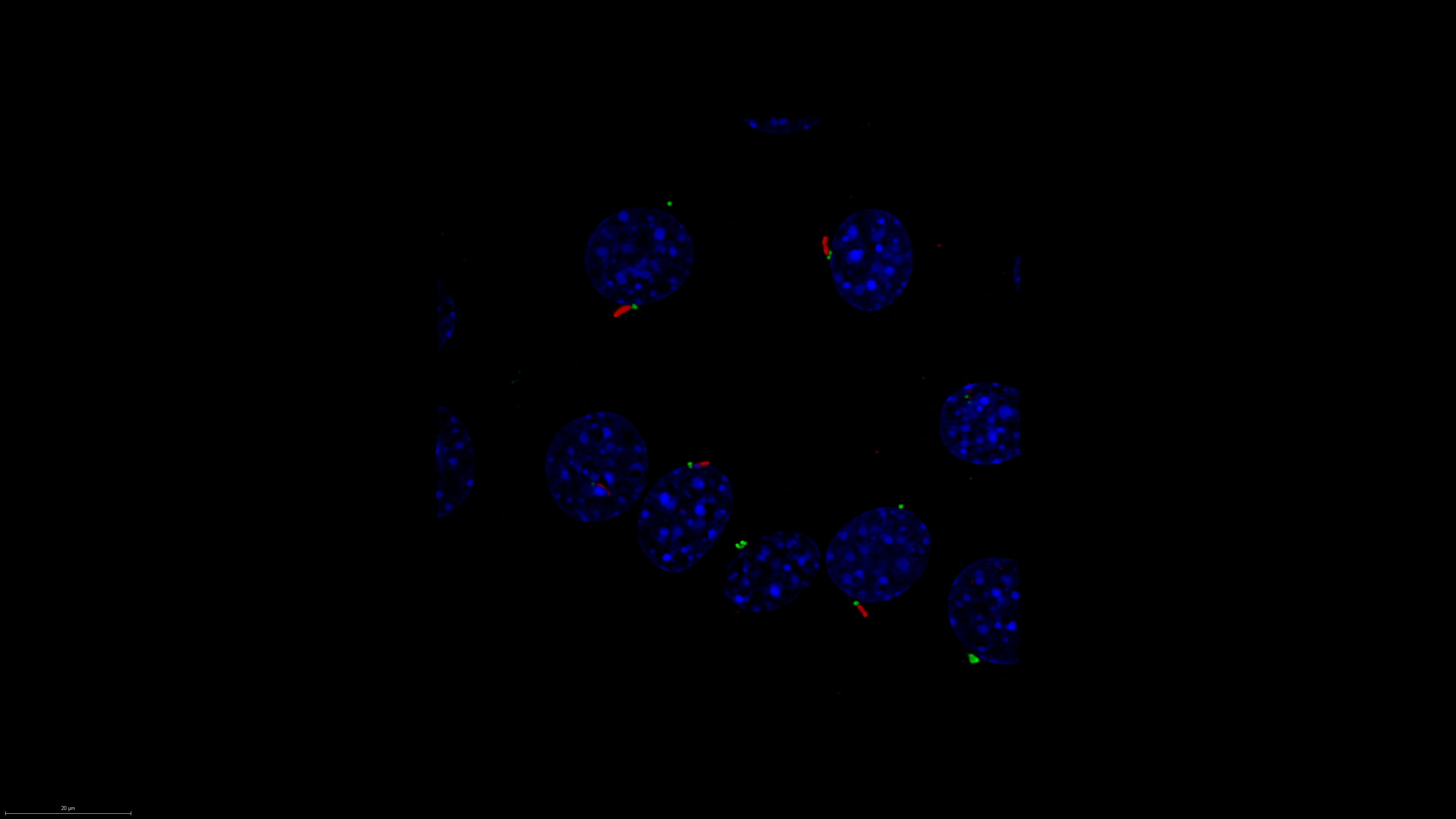

Supplement: Supplementary file 8 — Source Data for Figure 1 [file EMBR-24-e56870-s006.zip › Figure 1/1E/24h.tif]

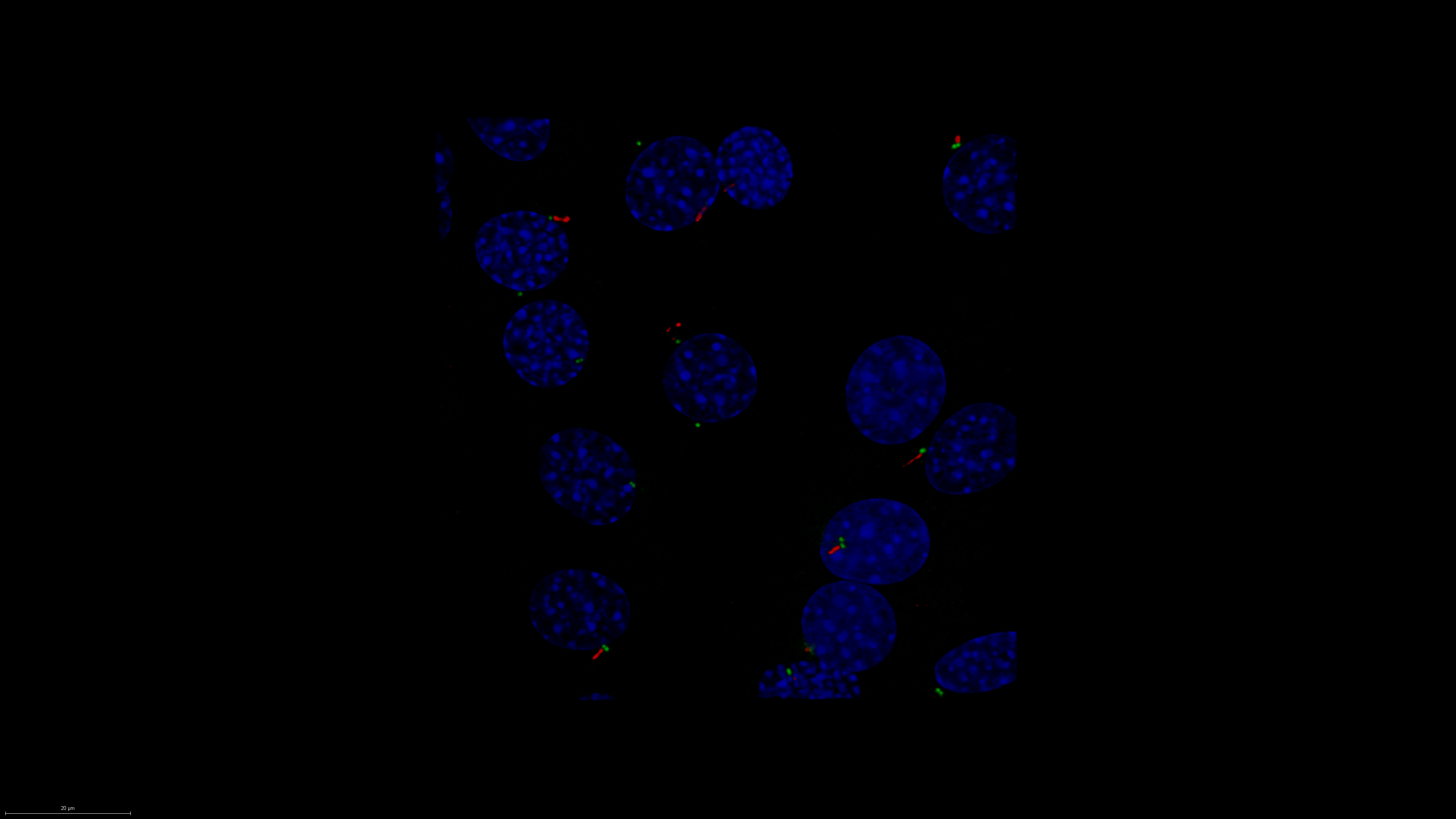

Supplement: Supplementary file 8 — Source Data for Figure 1 [file EMBR-24-e56870-s006.zip › Figure 1/1E/28 h.tif]

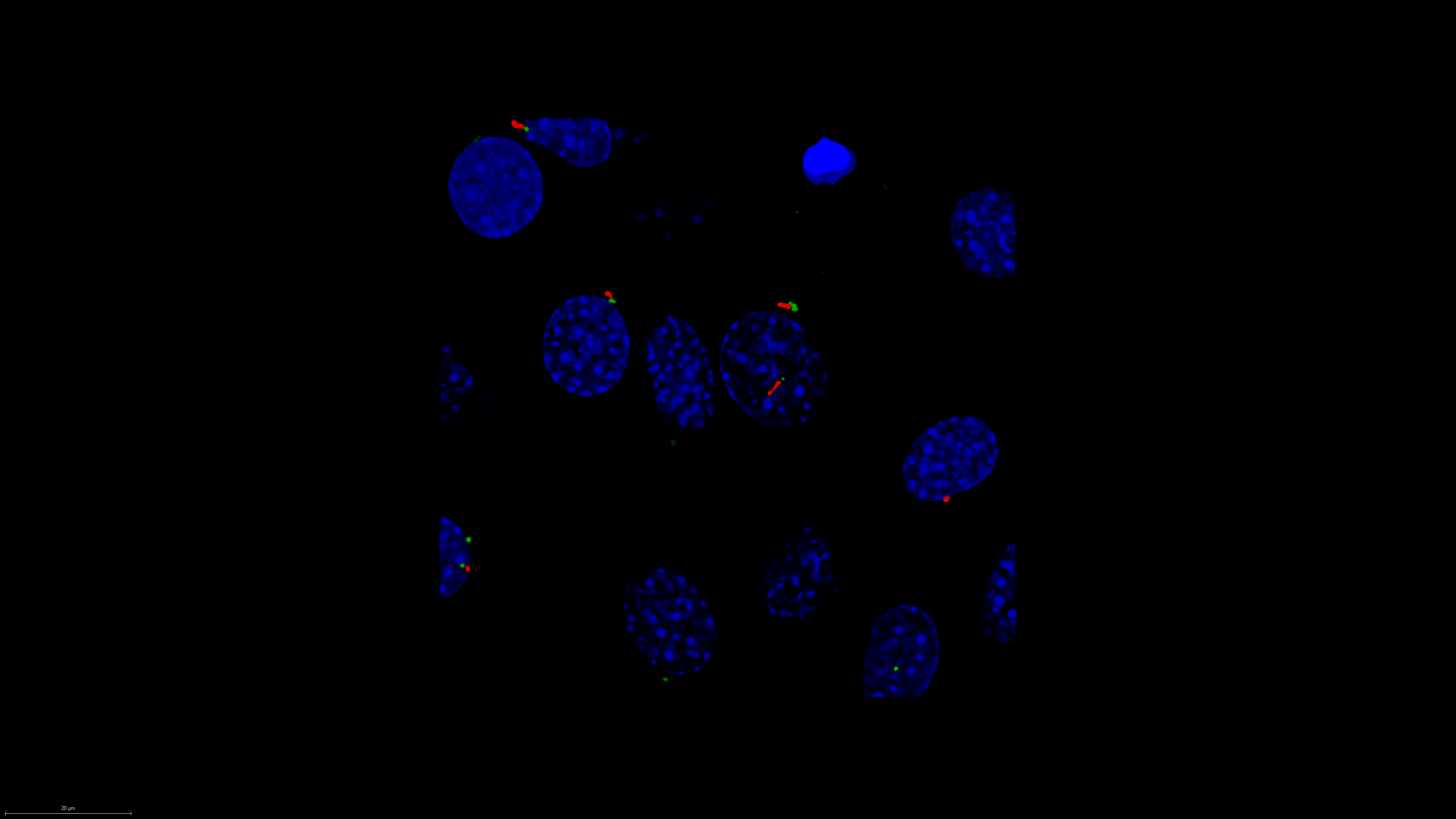

Supplement: Supplementary file 8 — Source Data for Figure 1 [file EMBR-24-e56870-s006.zip › Figure 1/1E/32h.tif]

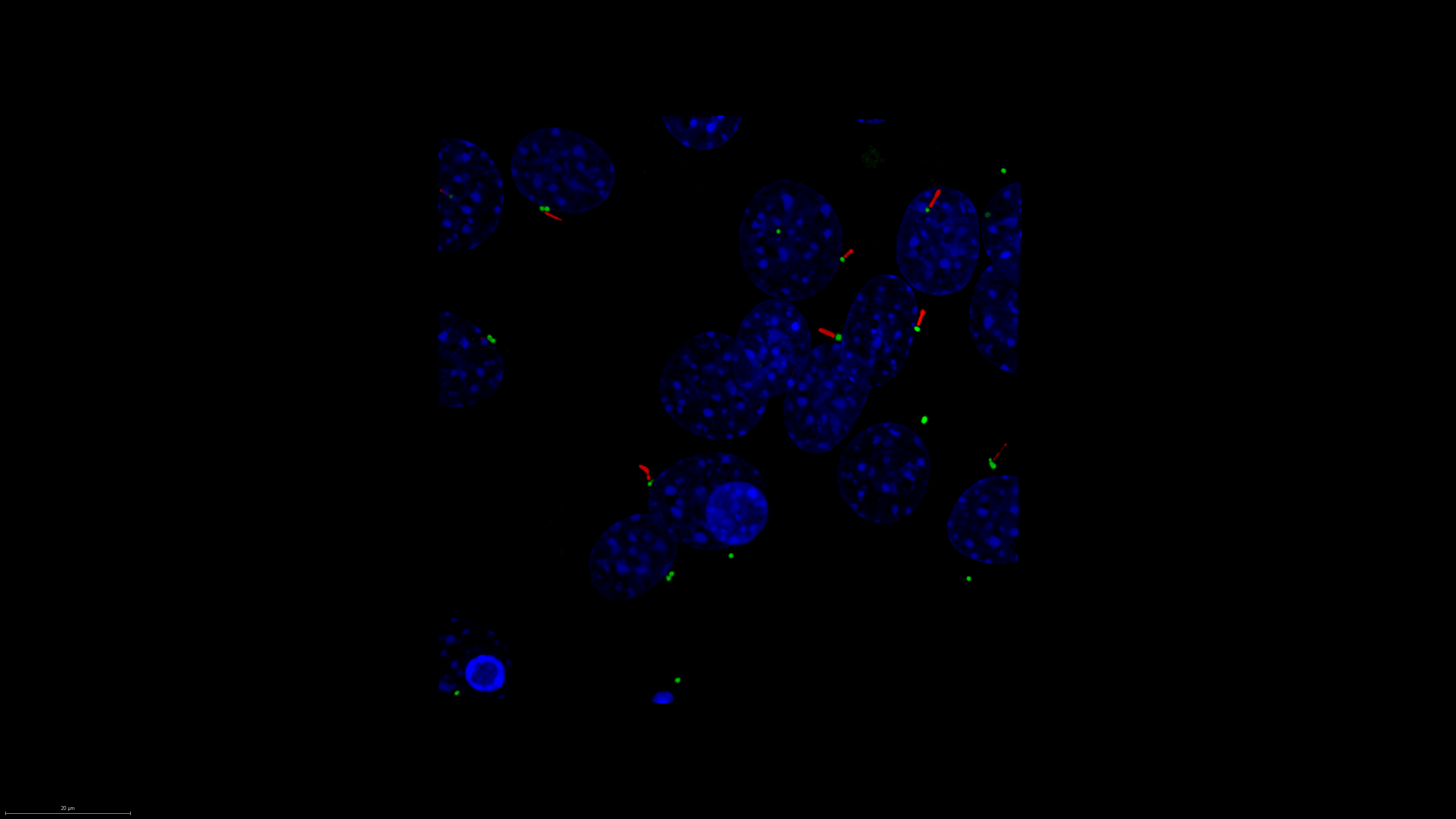

Supplement: Supplementary file 8 — Source Data for Figure 1 [file EMBR-24-e56870-s006.zip › Figure 1/1E/36h.tif]

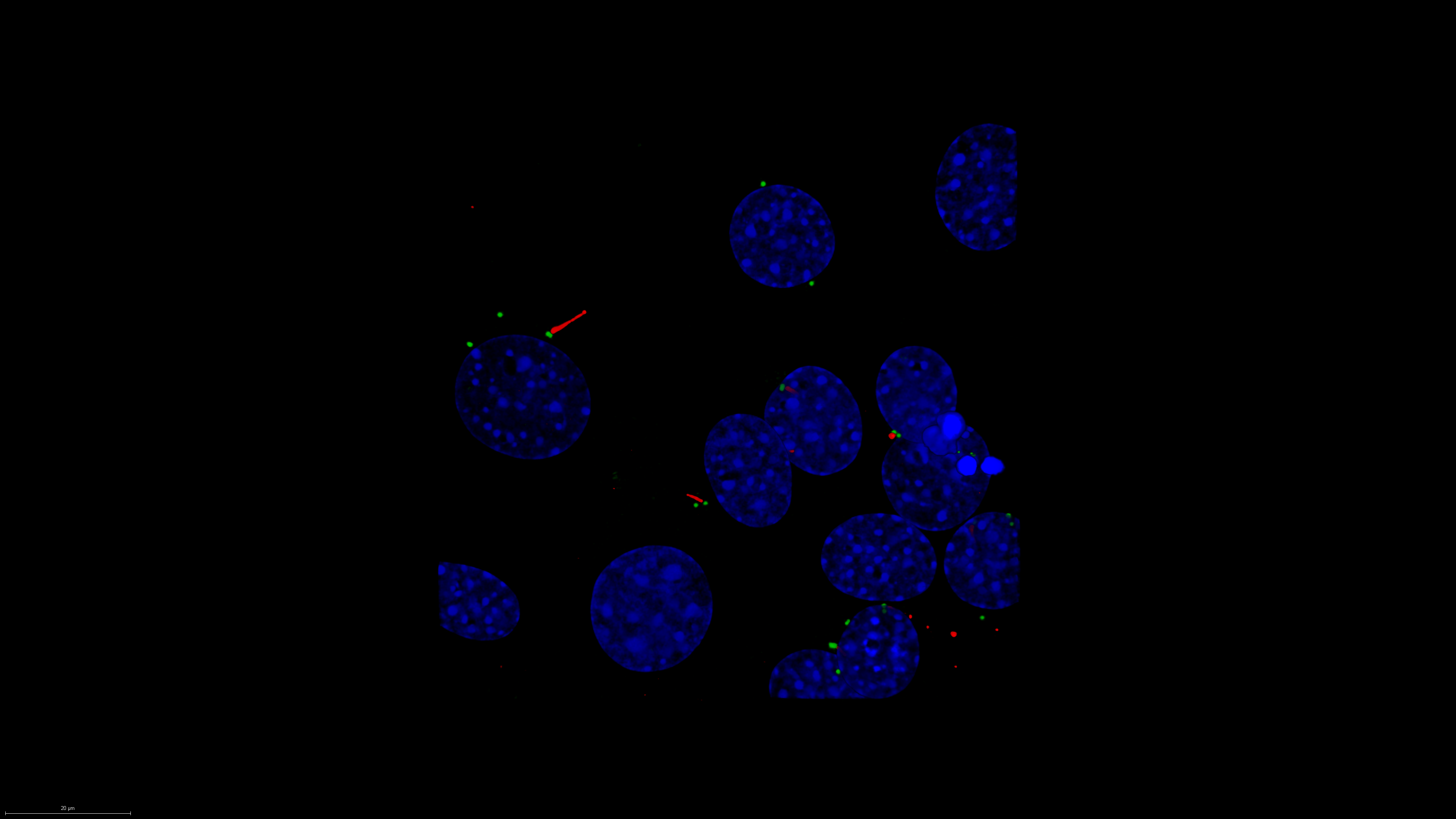

Supplement: Supplementary file 8 — Source Data for Figure 1 [file EMBR-24-e56870-s006.zip › Figure 1/1E/40 h.tif]

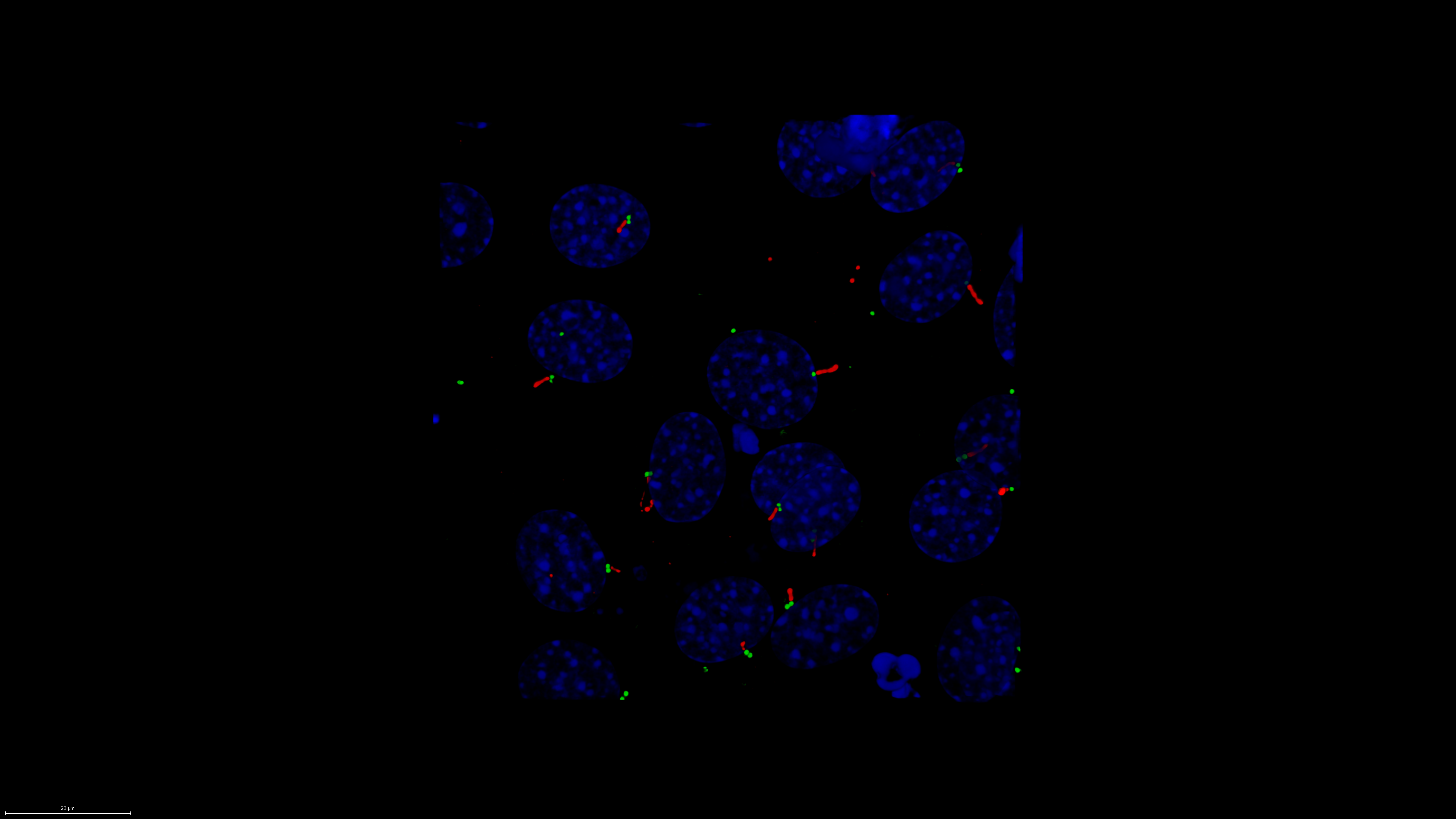

Supplement: Supplementary file 8 — Source Data for Figure 1 [file EMBR-24-e56870-s006.zip › Figure 1/1E/44h.tif]

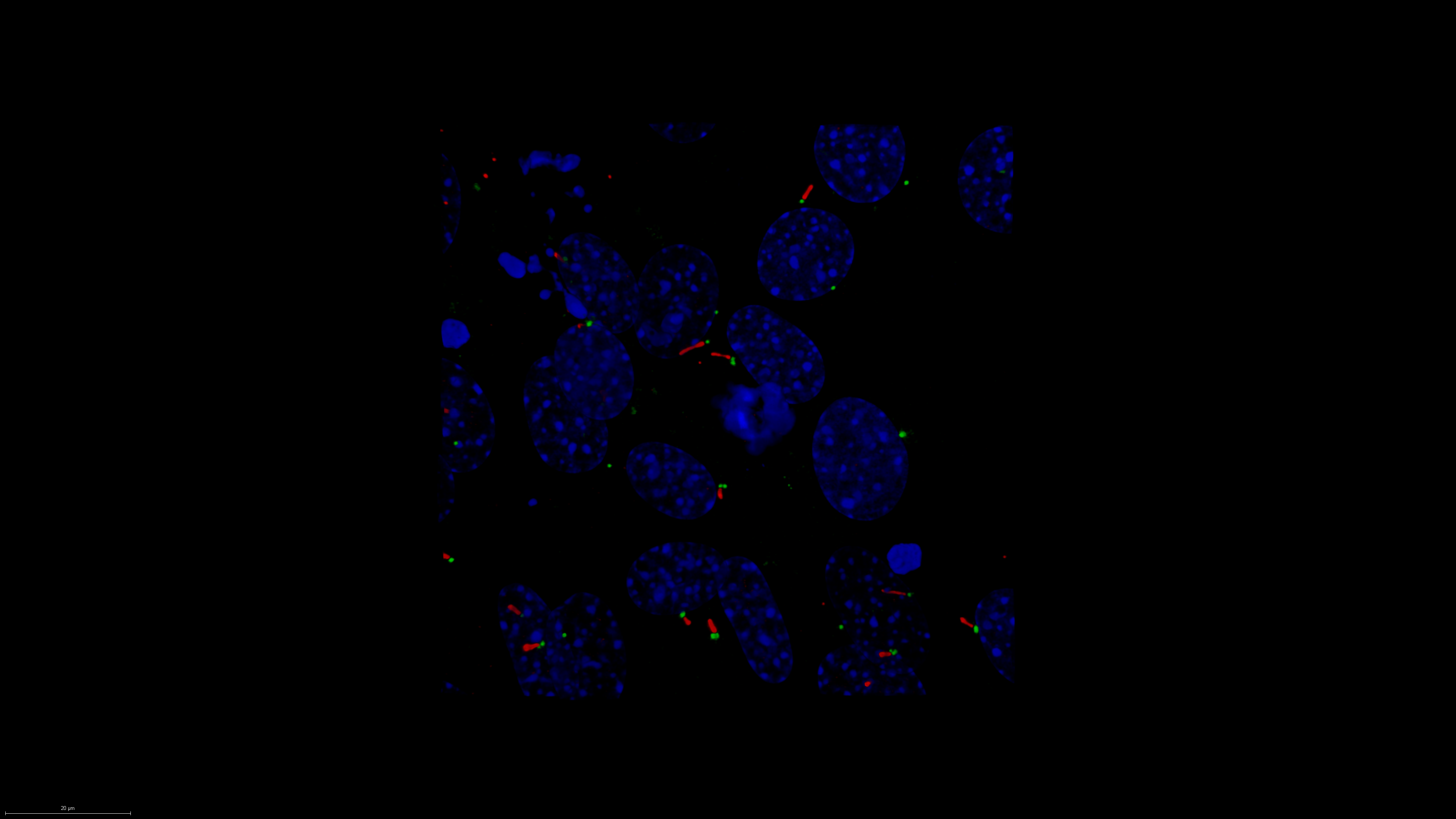

Supplement: Supplementary file 8 — Source Data for Figure 1 [file EMBR-24-e56870-s006.zip › Figure 1/1E/48h.tif]

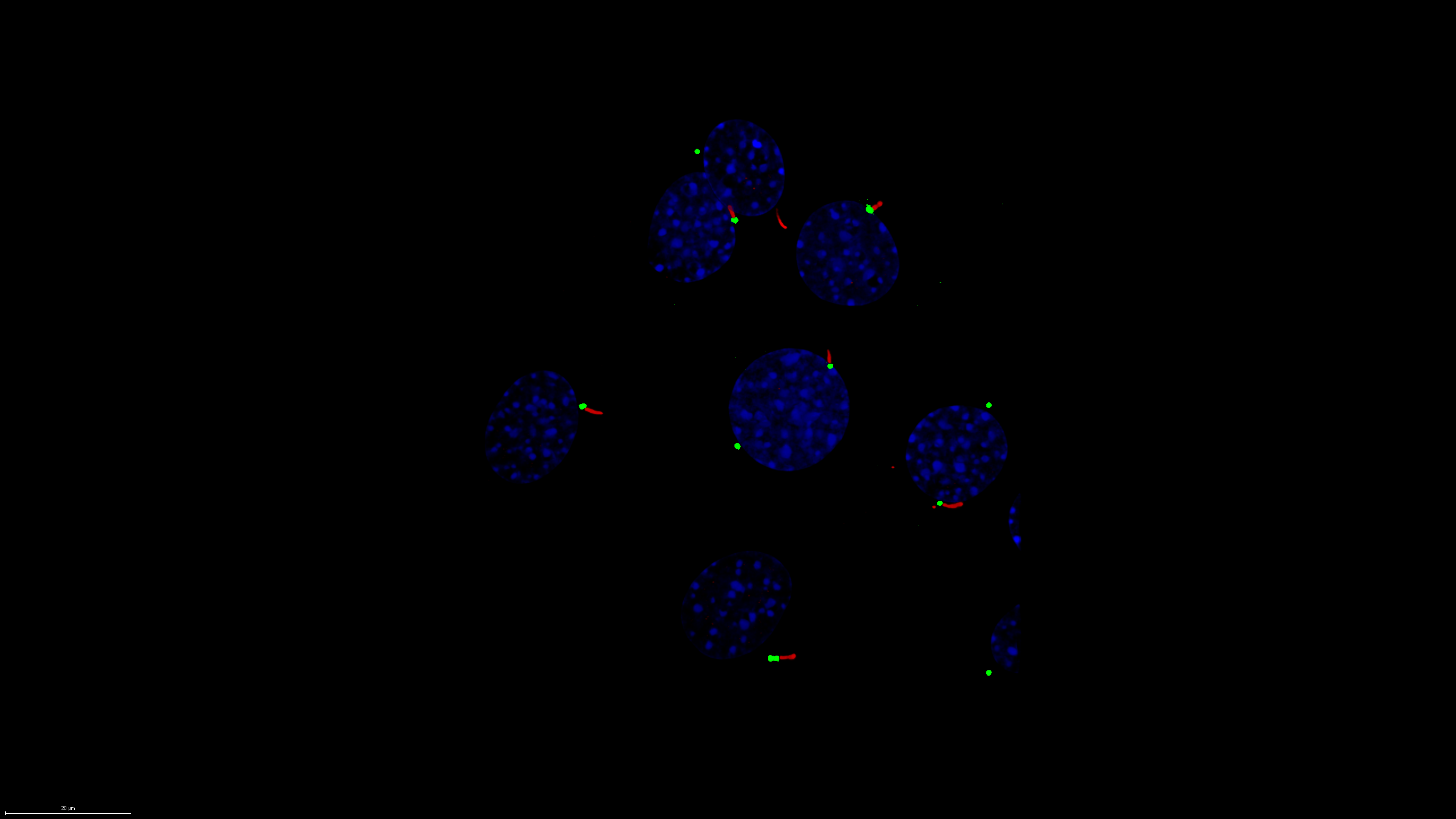

Supplement: Supplementary file 9 — Source Data for Figure 2 [file EMBR-24-e56870-s016.zip › Figure 2/2H/WT 32 h.tif]

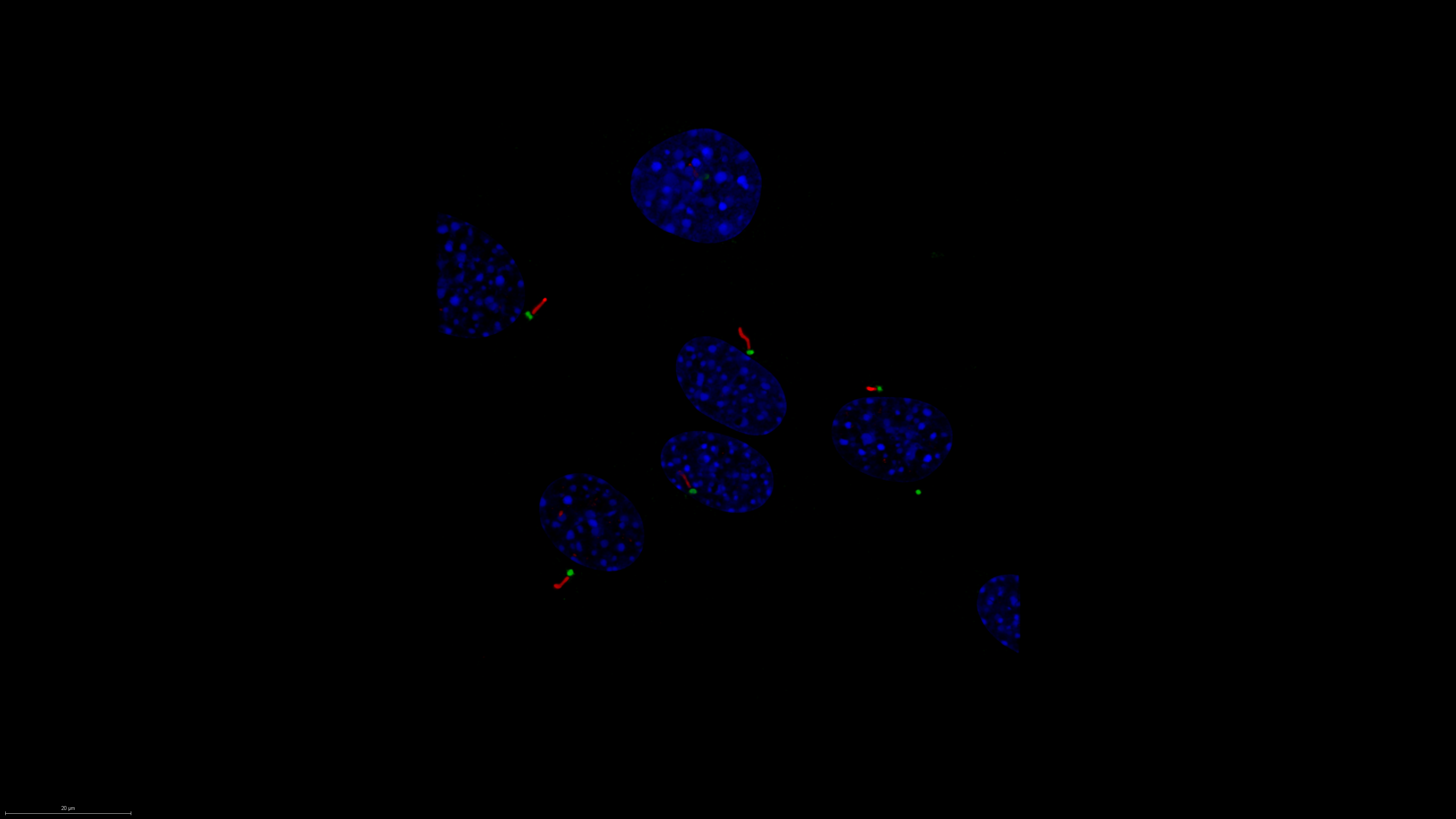

Supplement: Supplementary file 9 — Source Data for Figure 2 [file EMBR-24-e56870-s016.zip › Figure 2/2H/Bmal1 KO 32 h.tif]

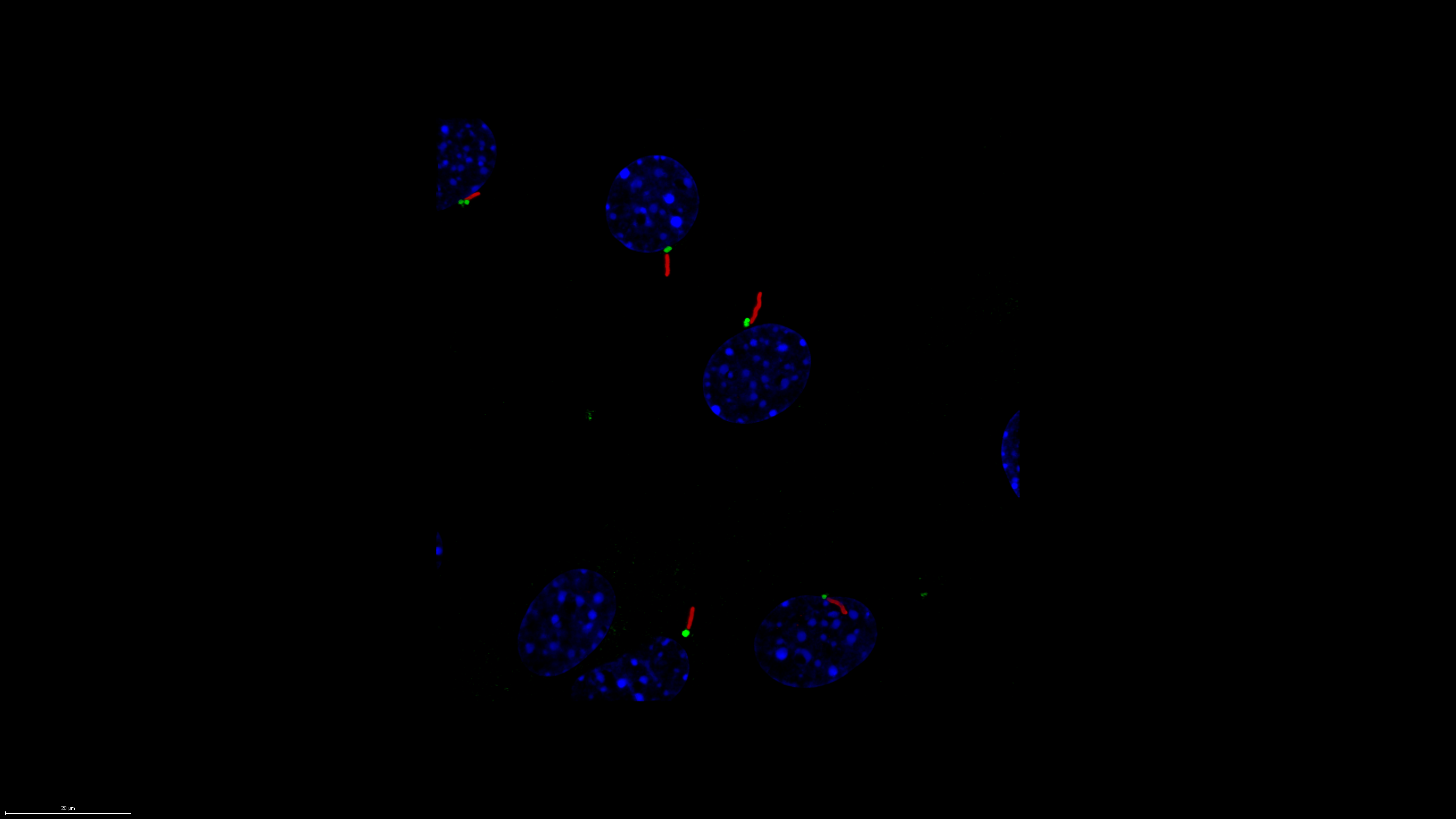

Supplement: Supplementary file 9 — Source Data for Figure 2 [file EMBR-24-e56870-s016.zip › Figure 2/2H/WT 44 h.tif]

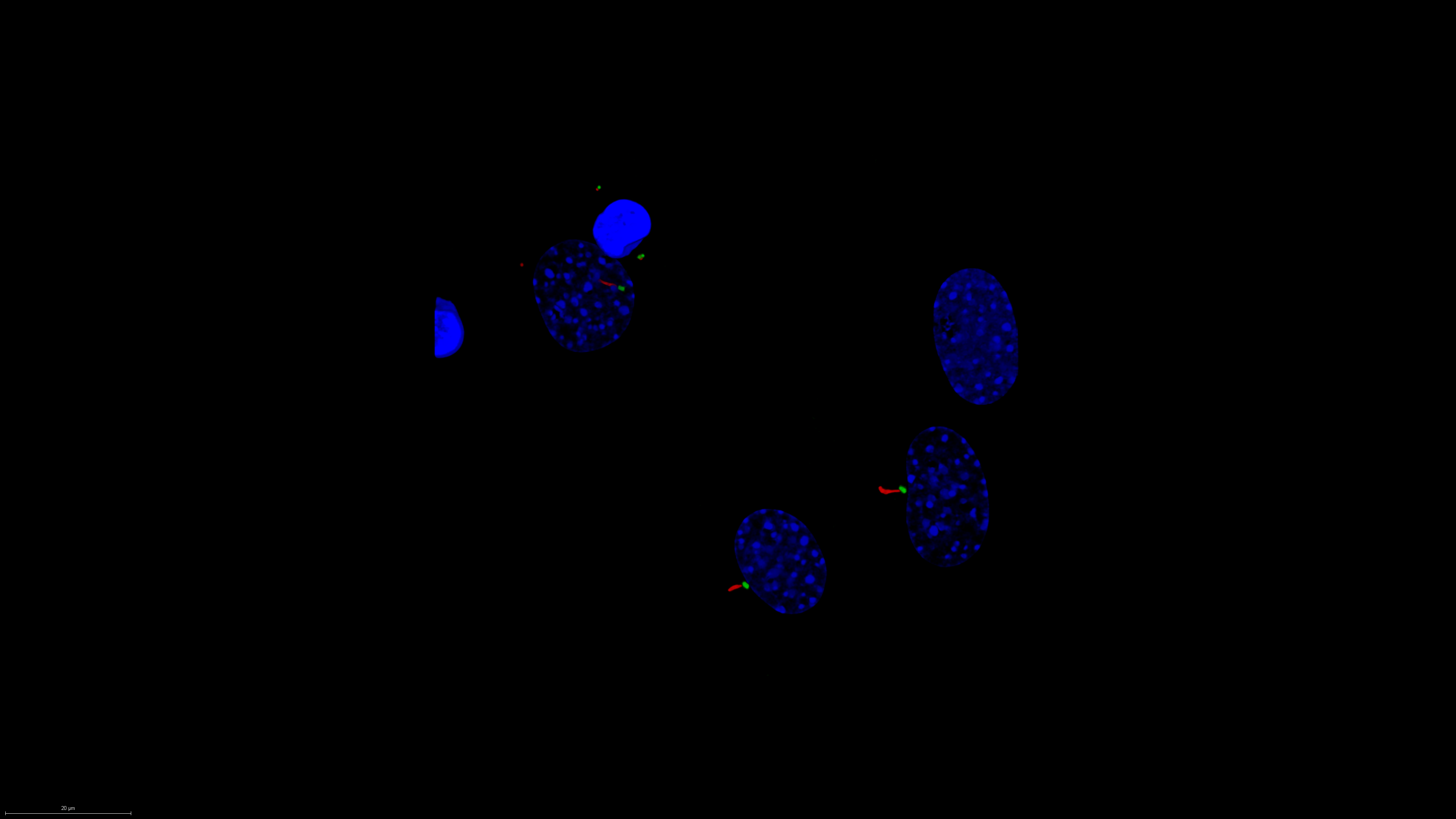

Supplement: Supplementary file 9 — Source Data for Figure 2 [file EMBR-24-e56870-s016.zip › Figure 2/2H/Bmal1 KO 44 h.tif]

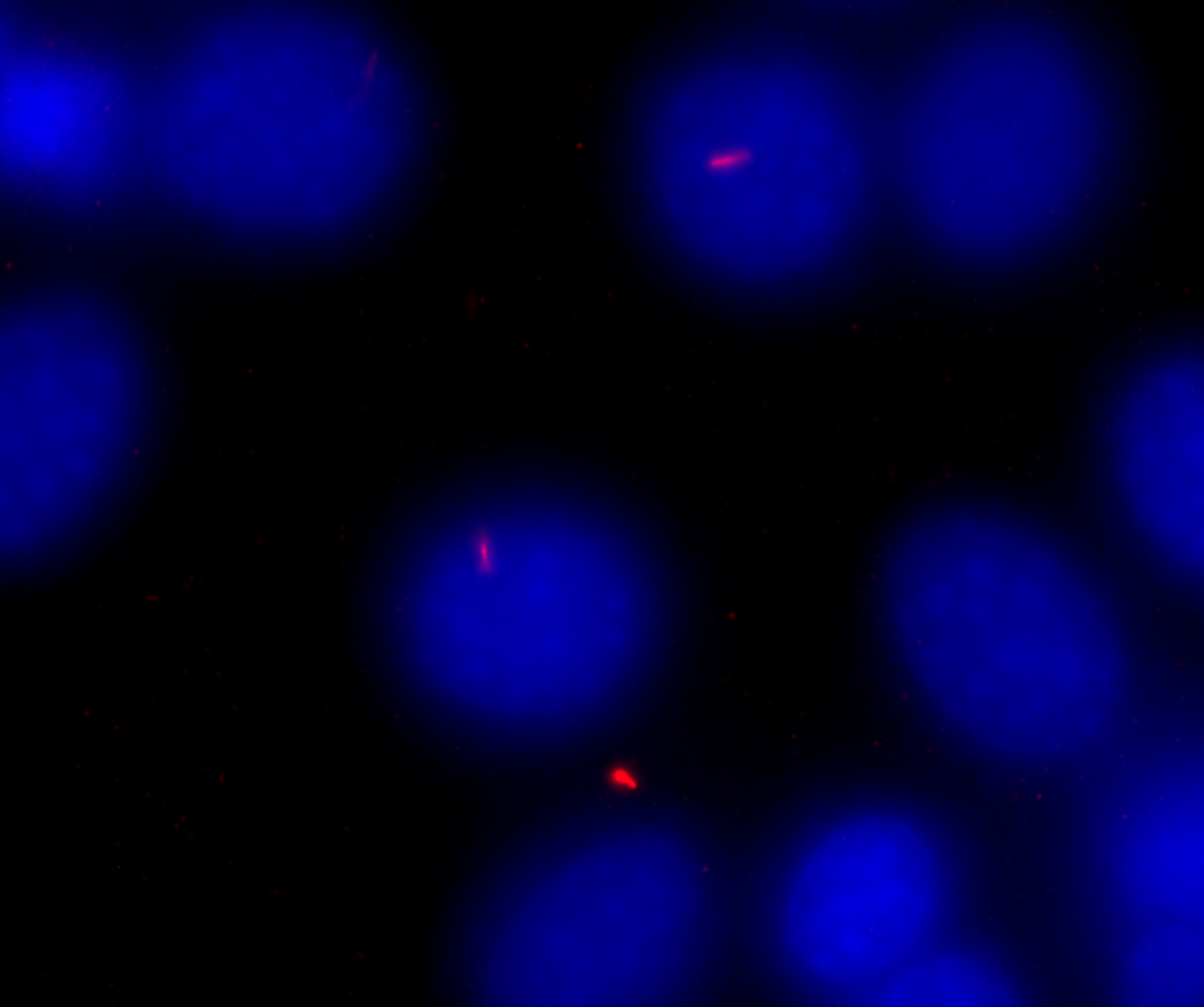

Supplement: Supplementary file 9 — Source Data for Figure 2 [file EMBR-24-e56870-s016.zip › Figure 2/2F/DEX 30 h.tif]

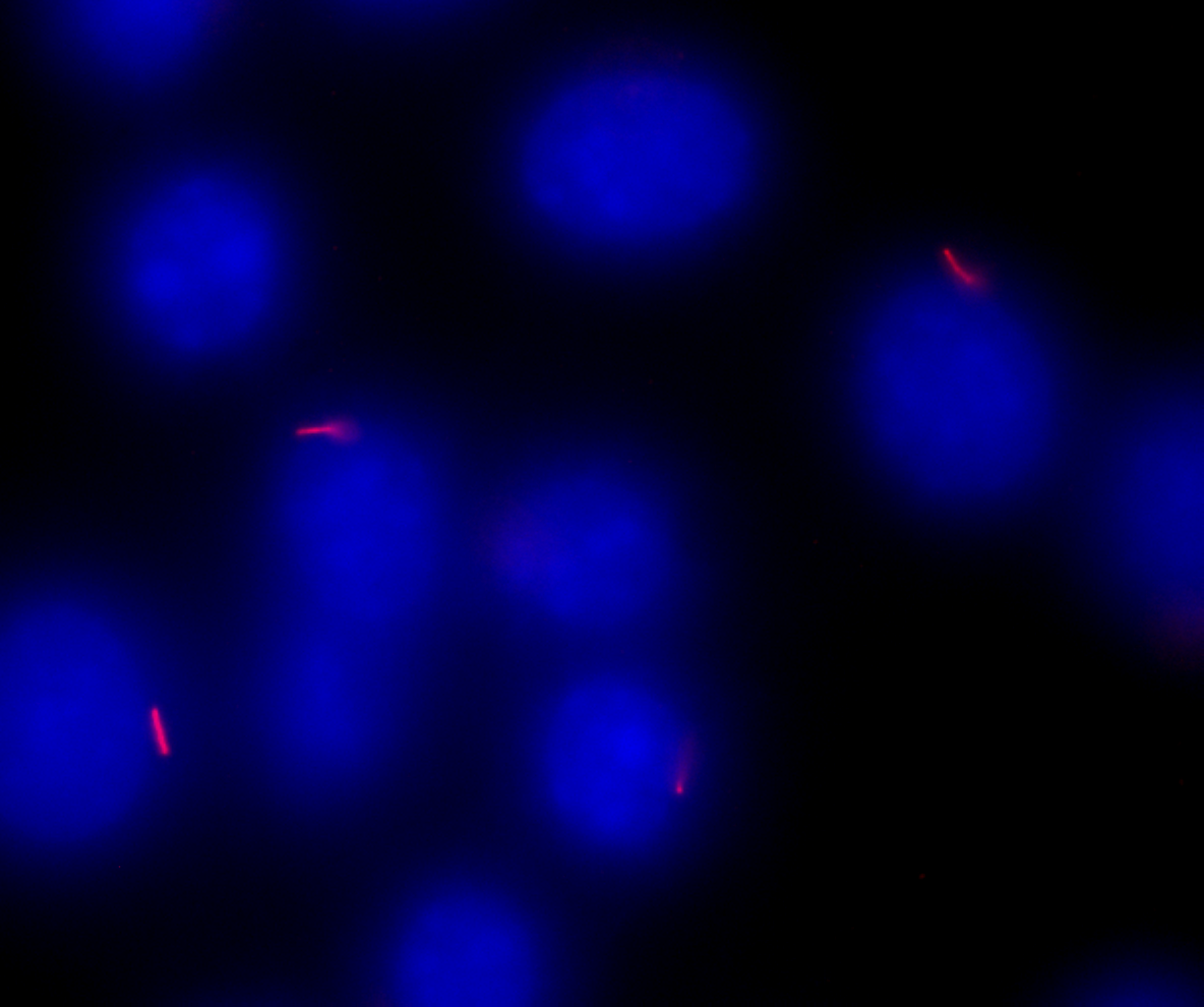

Supplement: Supplementary file 9 — Source Data for Figure 2 [file EMBR-24-e56870-s016.zip › Figure 2/2F/DEX+SR9011 30 h.tif]

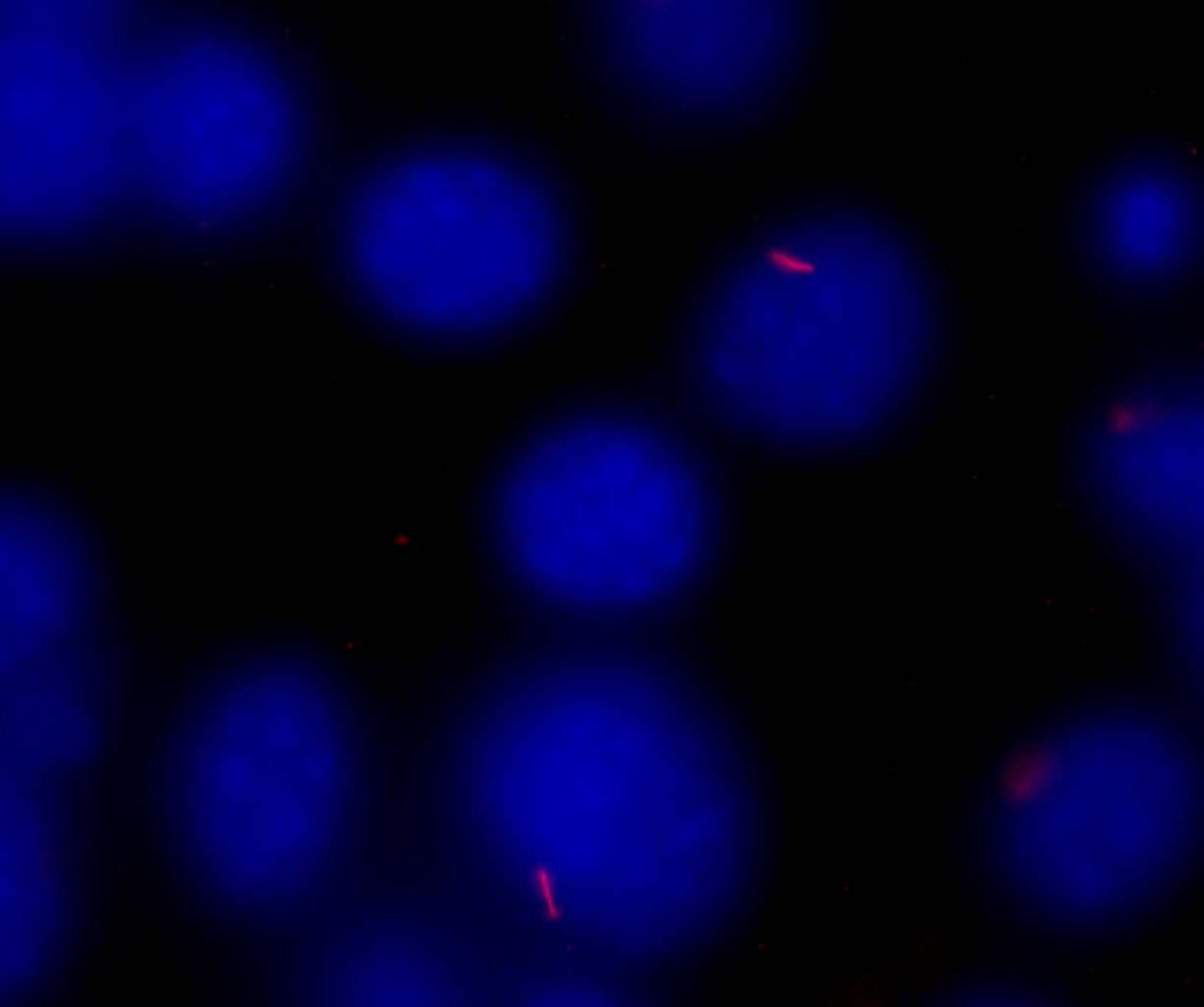

Supplement: Supplementary file 9 — Source Data for Figure 2 [file EMBR-24-e56870-s016.zip › Figure 2/2F/DEX+SR9011 42 h.tif]

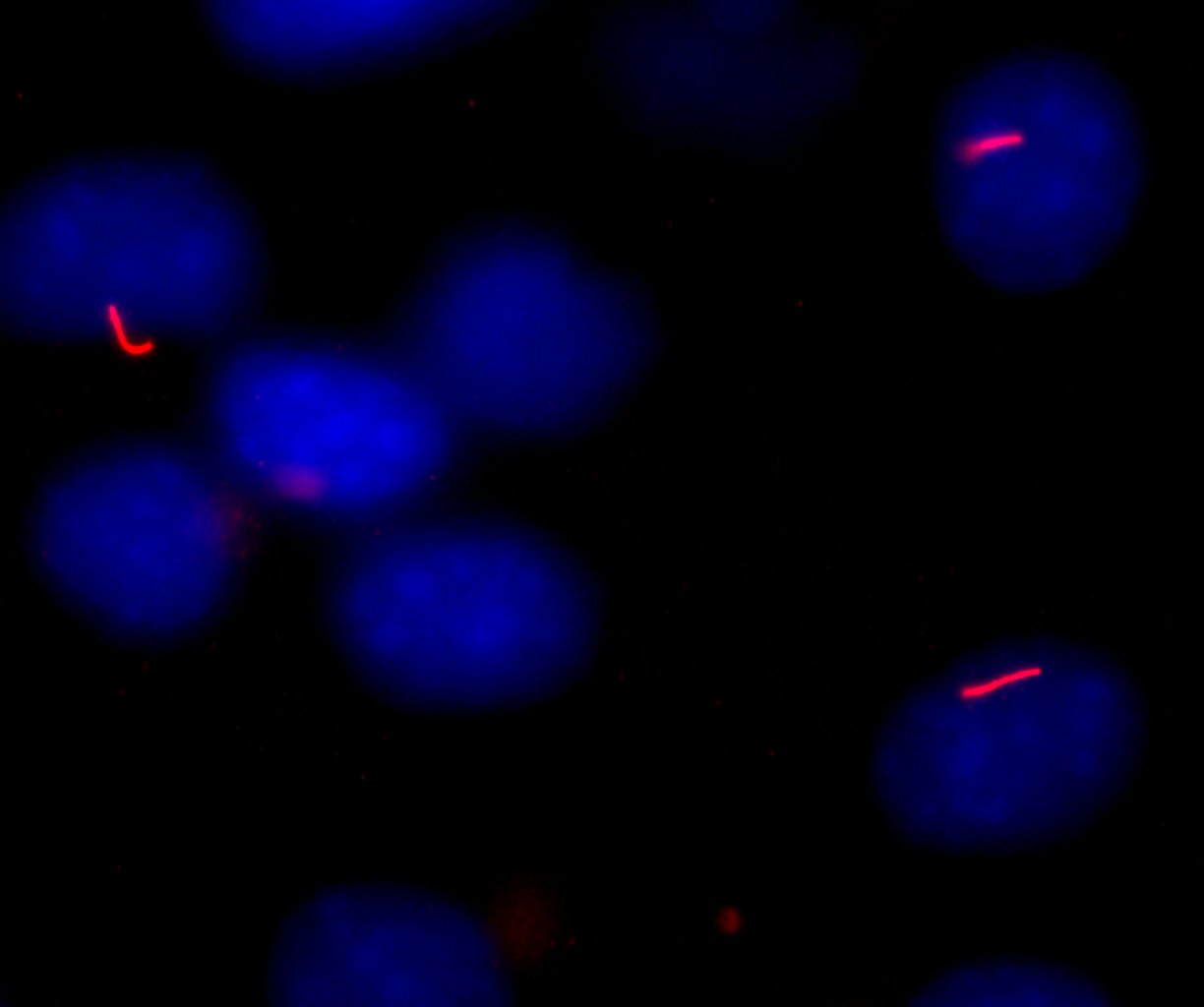

Supplement: Supplementary file 9 — Source Data for Figure 2 [file EMBR-24-e56870-s016.zip › Figure 2/2F/DEX 42 h.tif]

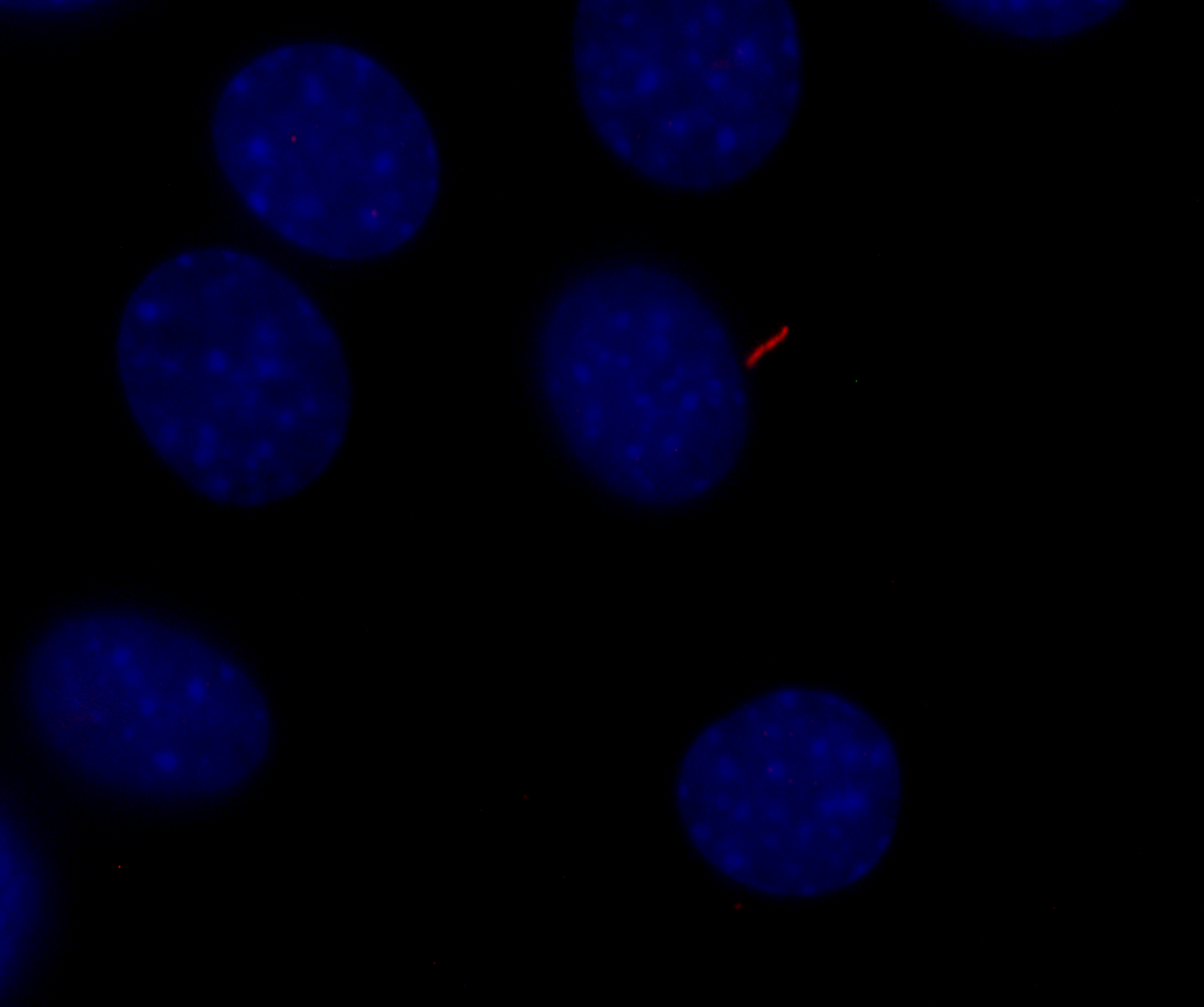

Supplement: Supplementary file 9 — Source Data for Figure 2 [file EMBR-24-e56870-s016.zip › Figure 2/2B/Control.tif]

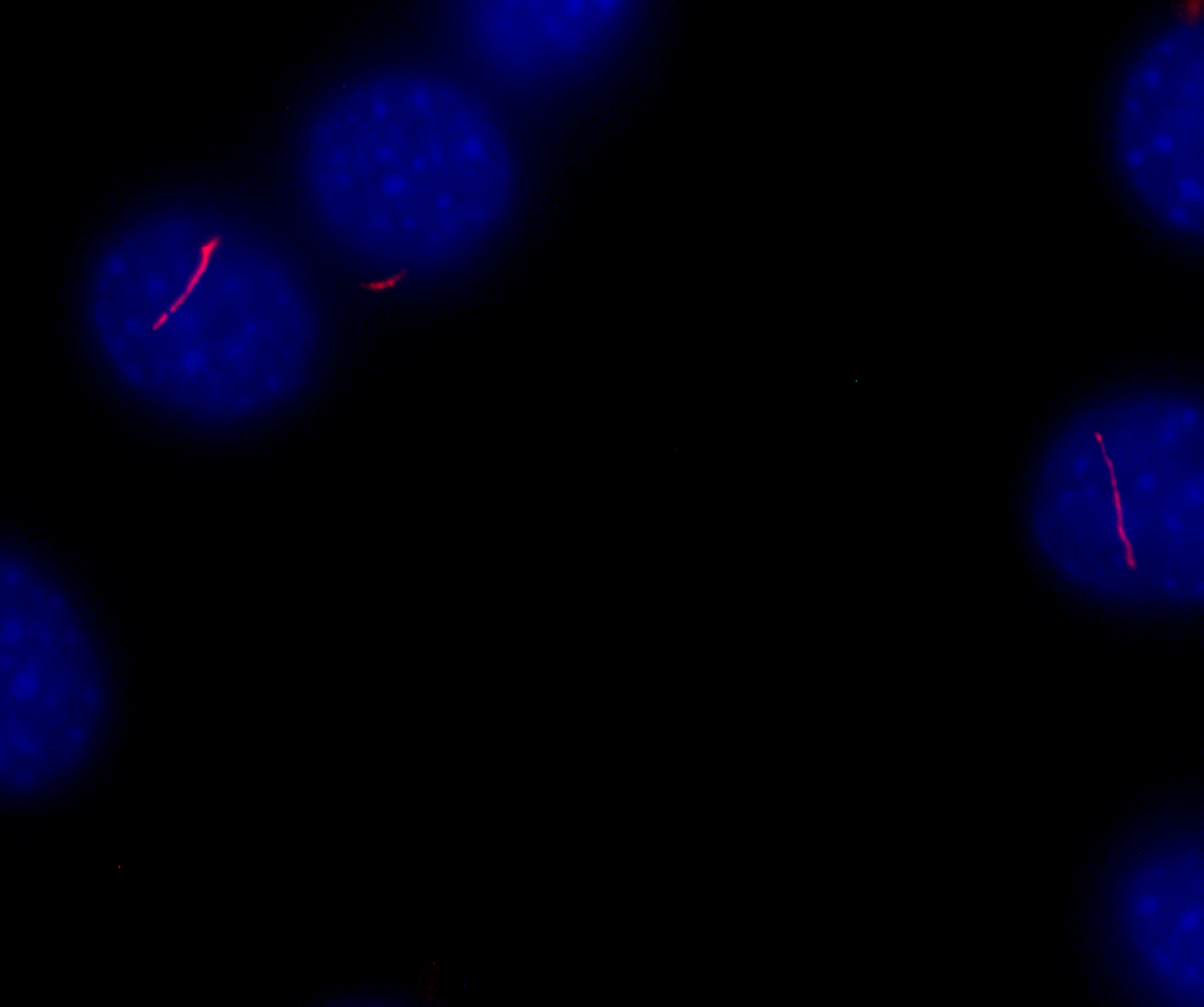

Supplement: Supplementary file 9 — Source Data for Figure 2 [file EMBR-24-e56870-s016.zip › Figure 2/2B/SR9011.tif]

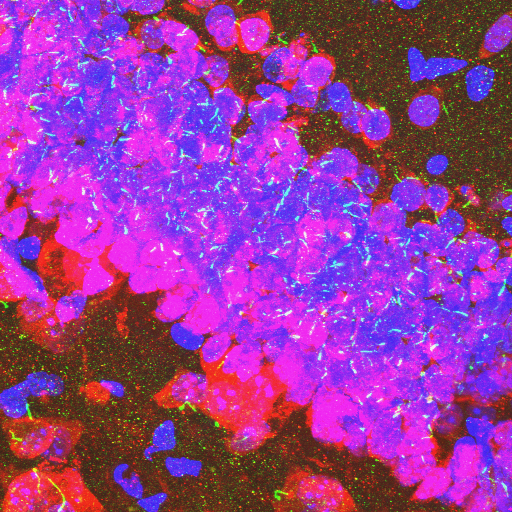

Supplement: Supplementary file 10 — Source Data for Figure 3 [file EMBR-24-e56870-s014.zip › Figure 3/3B/ZT17.tif]

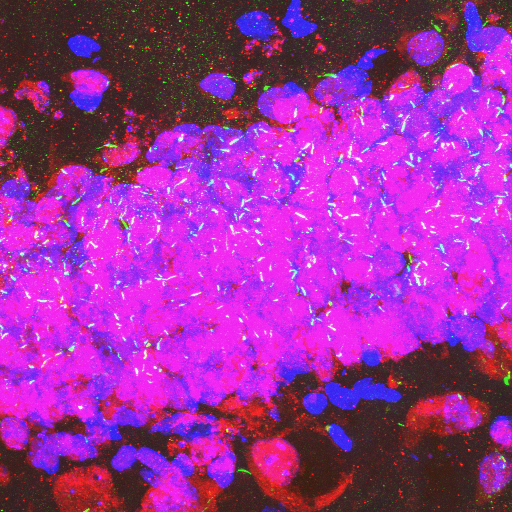

Supplement: Supplementary file 10 — Source Data for Figure 3 [file EMBR-24-e56870-s014.zip › Figure 3/3B/ZT5.tif]

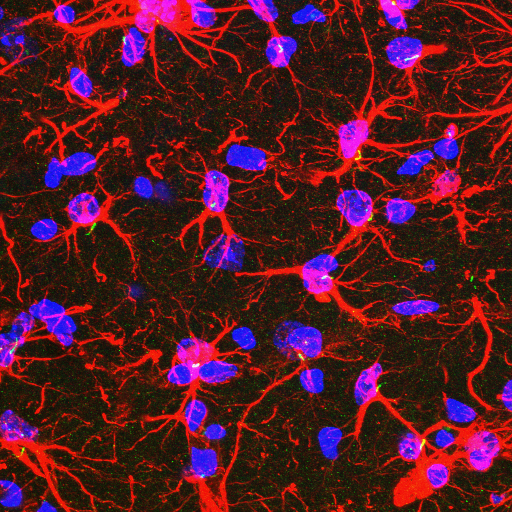

Supplement: Supplementary file 10 — Source Data for Figure 3 [file EMBR-24-e56870-s014.zip › Figure 3/3D/ZT5 GFAP.tif]

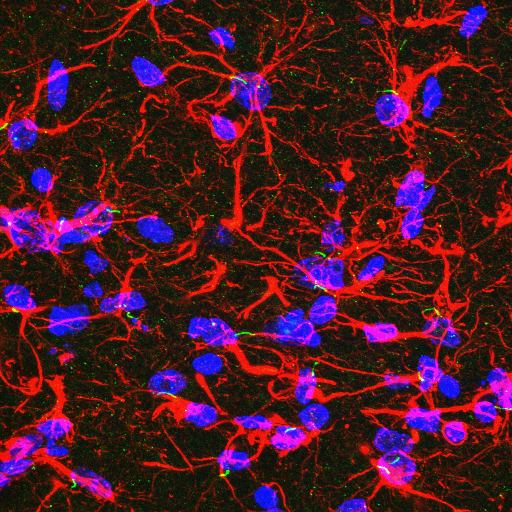

Supplement: Supplementary file 10 — Source Data for Figure 3 [file EMBR-24-e56870-s014.zip › Figure 3/3D/ZT17 GFAP.tif]

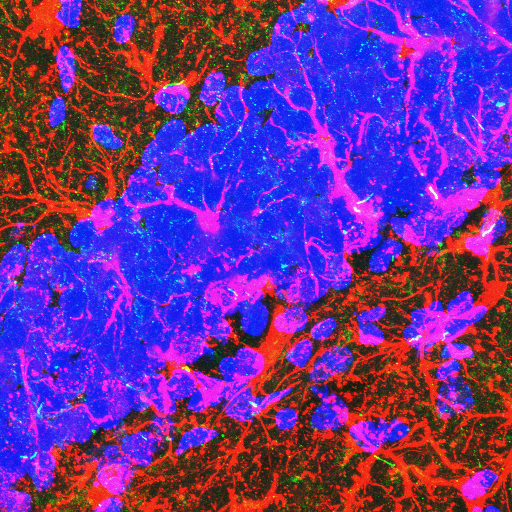

Supplement: Supplementary file 10 — Source Data for Figure 3 [file EMBR-24-e56870-s014.zip › Figure 3/3A/GFAP Arl13b.tif]

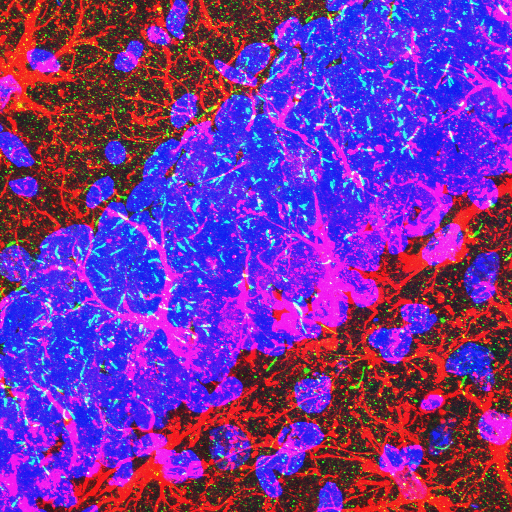

Supplement: Supplementary file 10 — Source Data for Figure 3 [file EMBR-24-e56870-s014.zip › Figure 3/3A/GFAP AC3.tif]

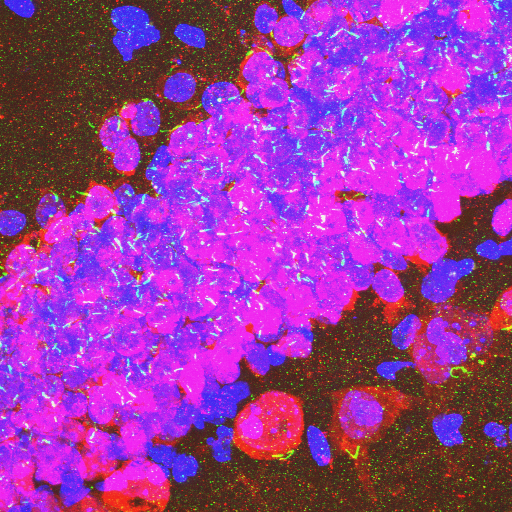

Supplement: Supplementary file 10 — Source Data for Figure 3 [file EMBR-24-e56870-s014.zip › Figure 3/3A/NeuN AC3 DAPI.tif]

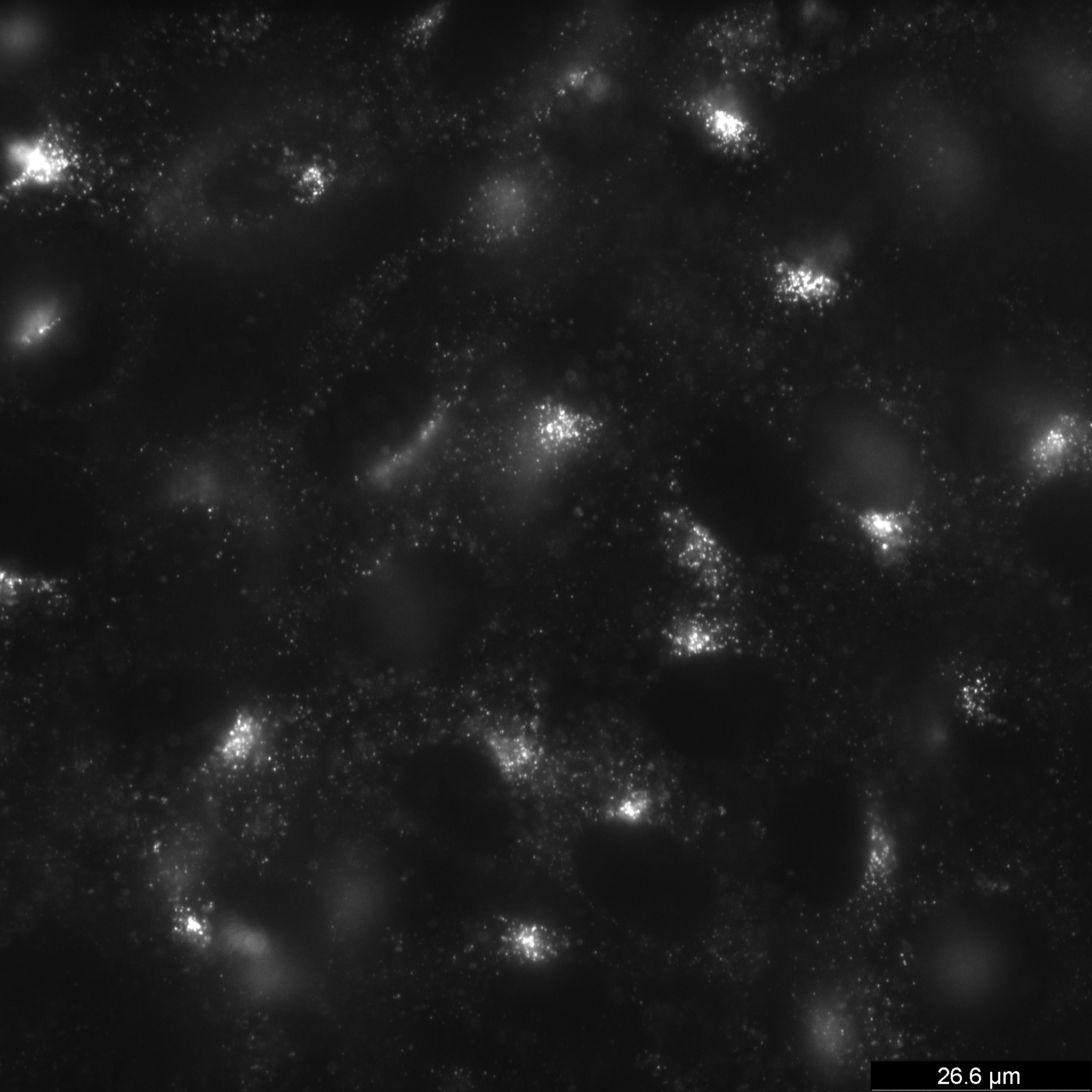

Supplement: Supplementary file 11 — Source Data for Figure 4 [file EMBR-24-e56870-s012.zip › Figure 4/4E/DEX 32 h.tif]

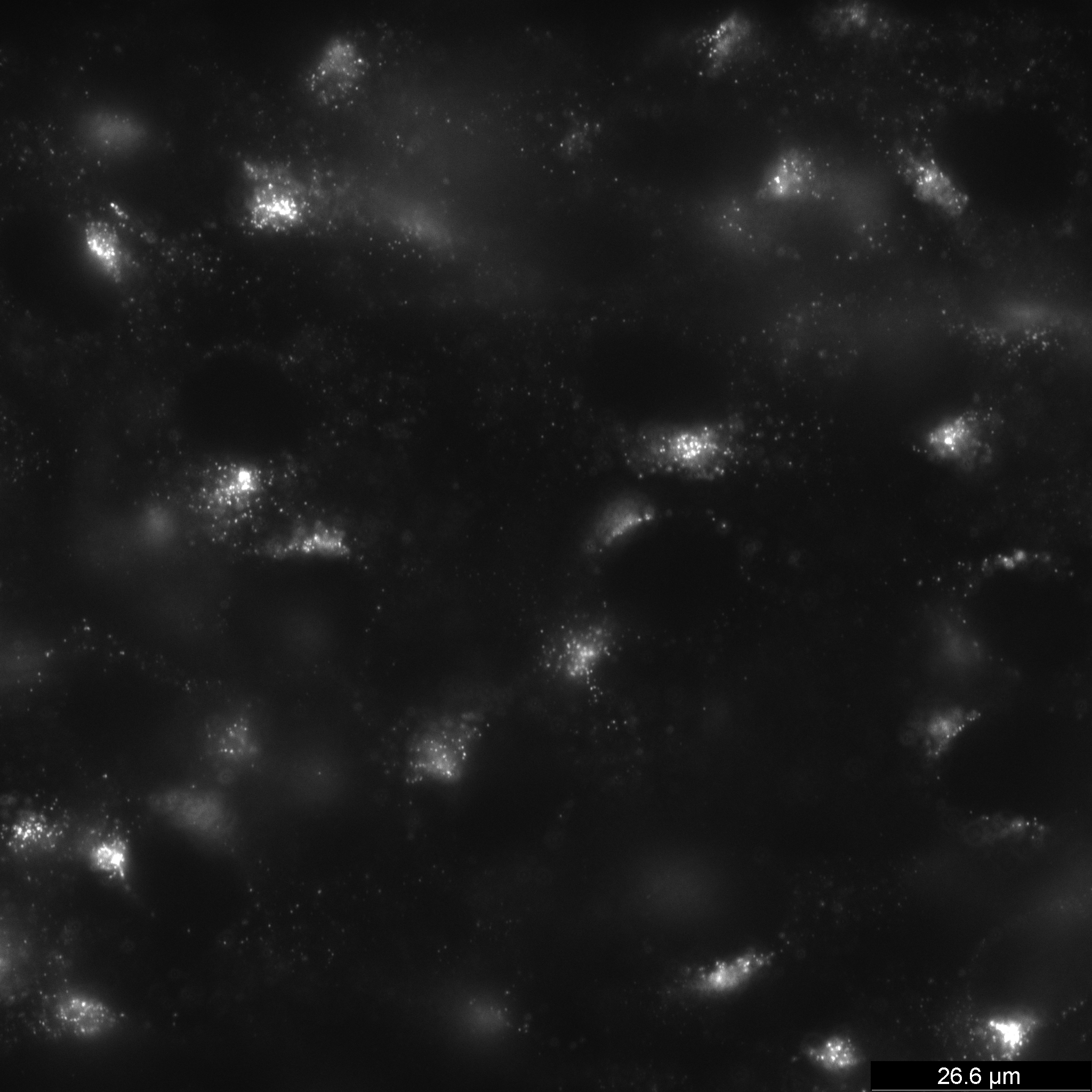

Supplement: Supplementary file 11 — Source Data for Figure 4 [file EMBR-24-e56870-s012.zip › Figure 4/4E/DEX 48 h.tif]

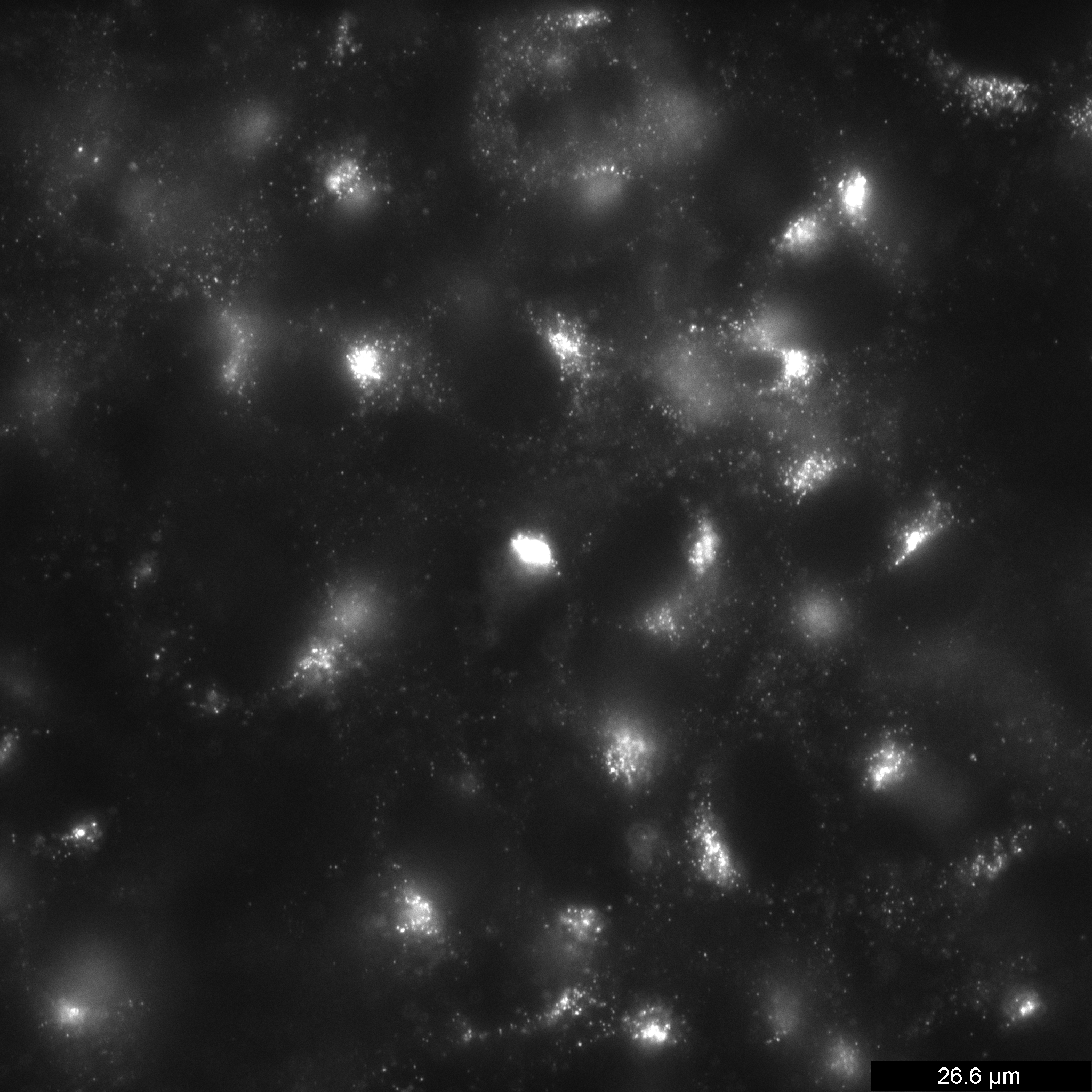

Supplement: Supplementary file 11 — Source Data for Figure 4 [file EMBR-24-e56870-s012.zip › Figure 4/4E/DEX+SR9011 28 h.tif]

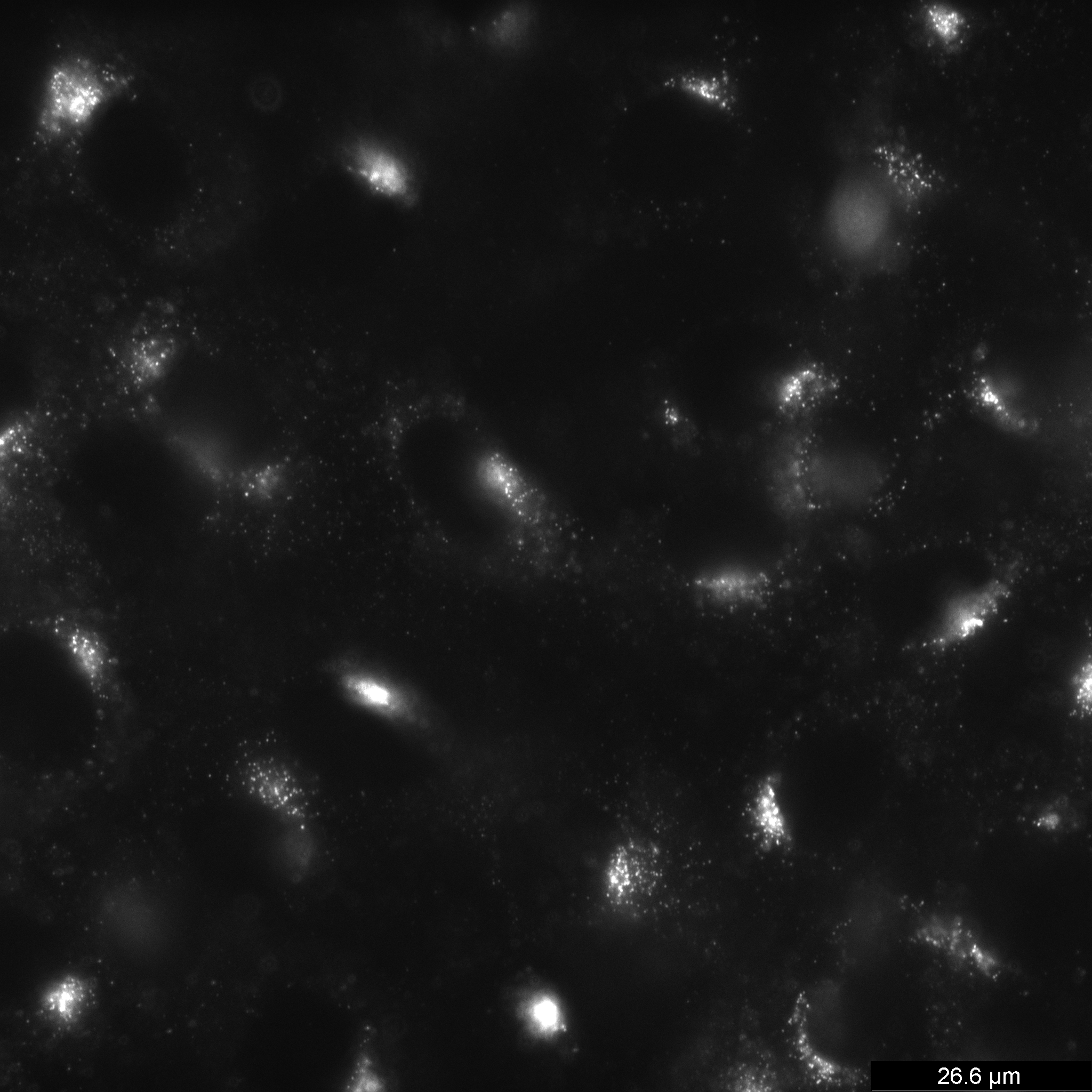

Supplement: Supplementary file 11 — Source Data for Figure 4 [file EMBR-24-e56870-s012.zip › Figure 4/4E/DEX 36 h.tif]

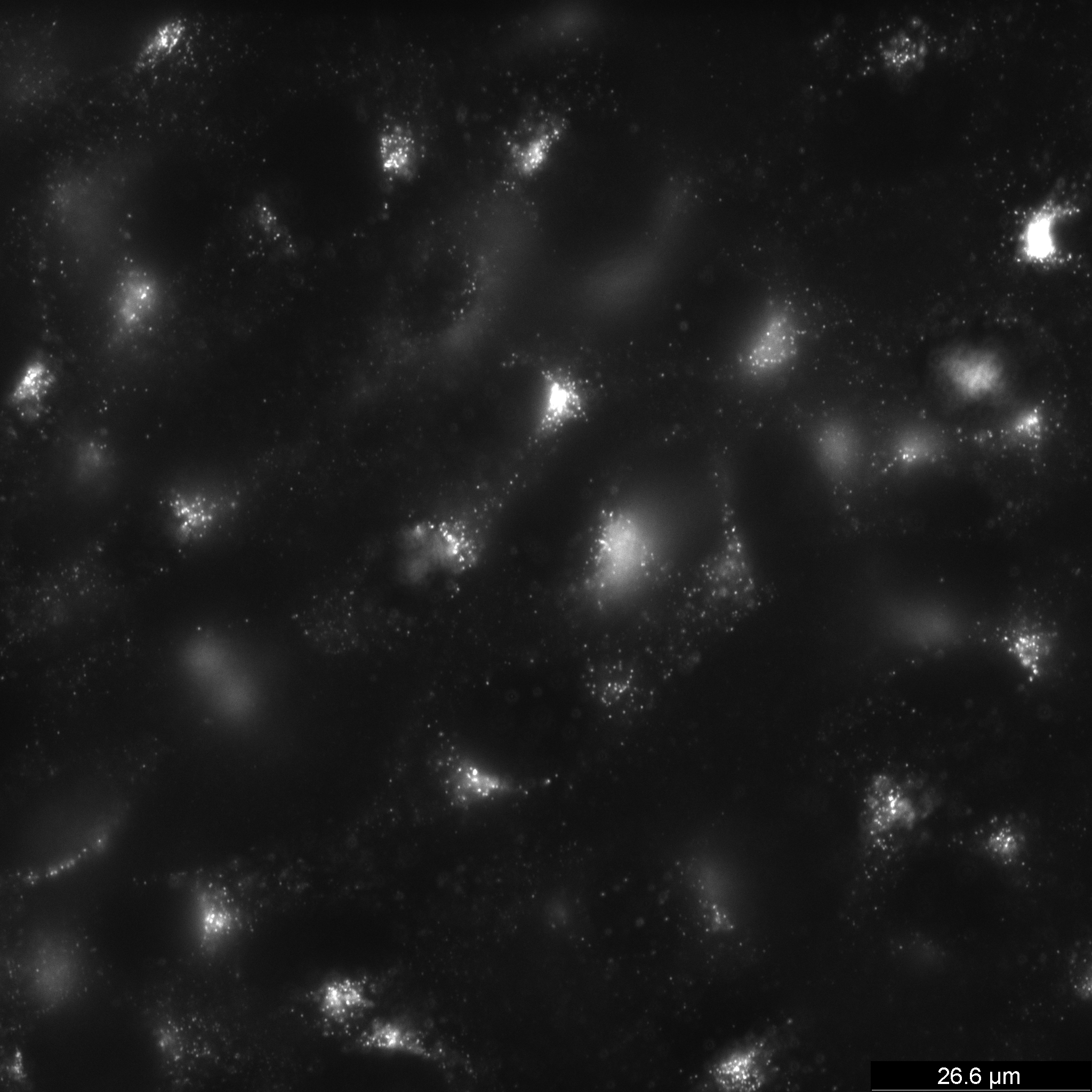

Supplement: Supplementary file 11 — Source Data for Figure 4 [file EMBR-24-e56870-s012.zip › Figure 4/4E/DEX 28 h.tif]

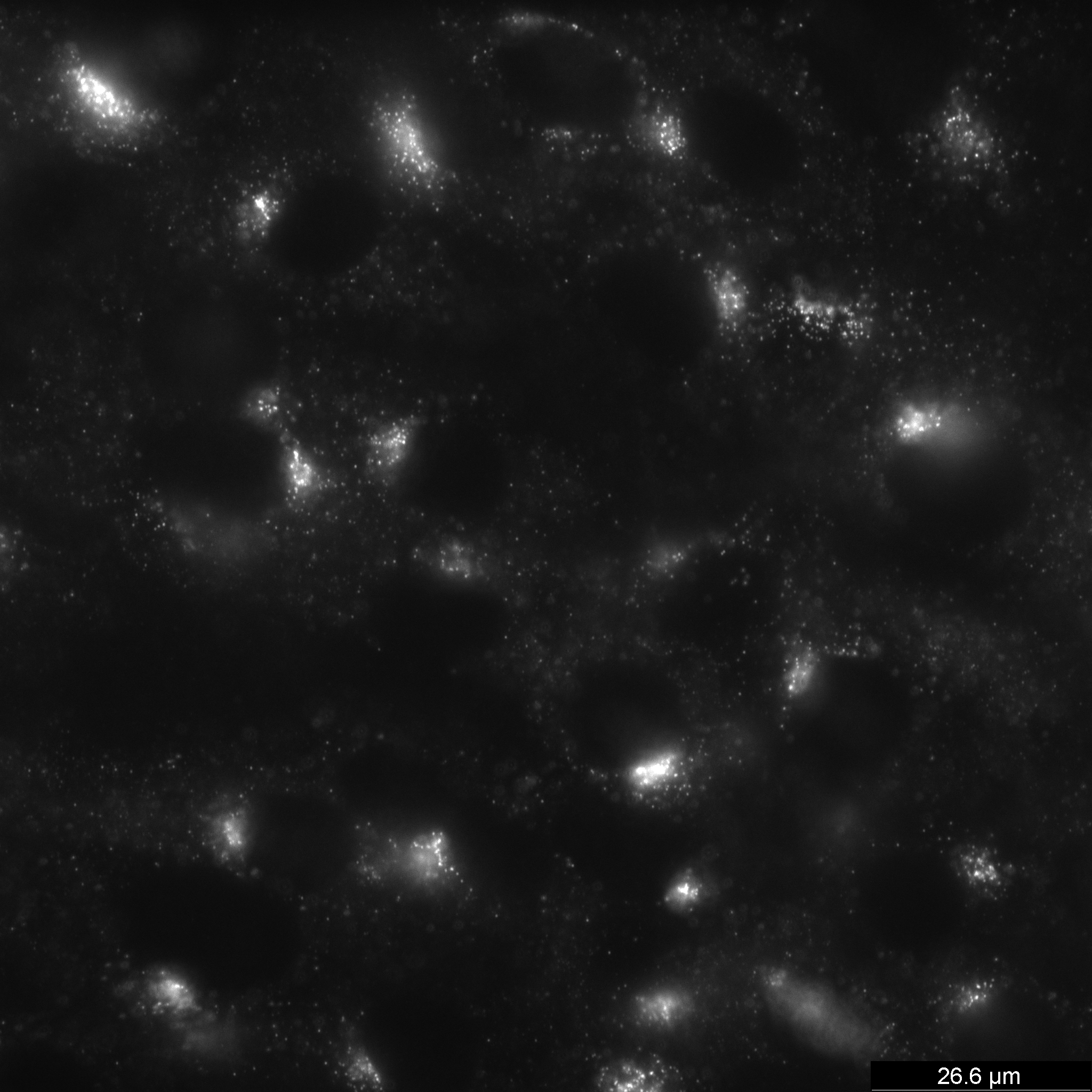

Supplement: Supplementary file 11 — Source Data for Figure 4 [file EMBR-24-e56870-s012.zip › Figure 4/4E/DEX+SR9011 36 h.tif]

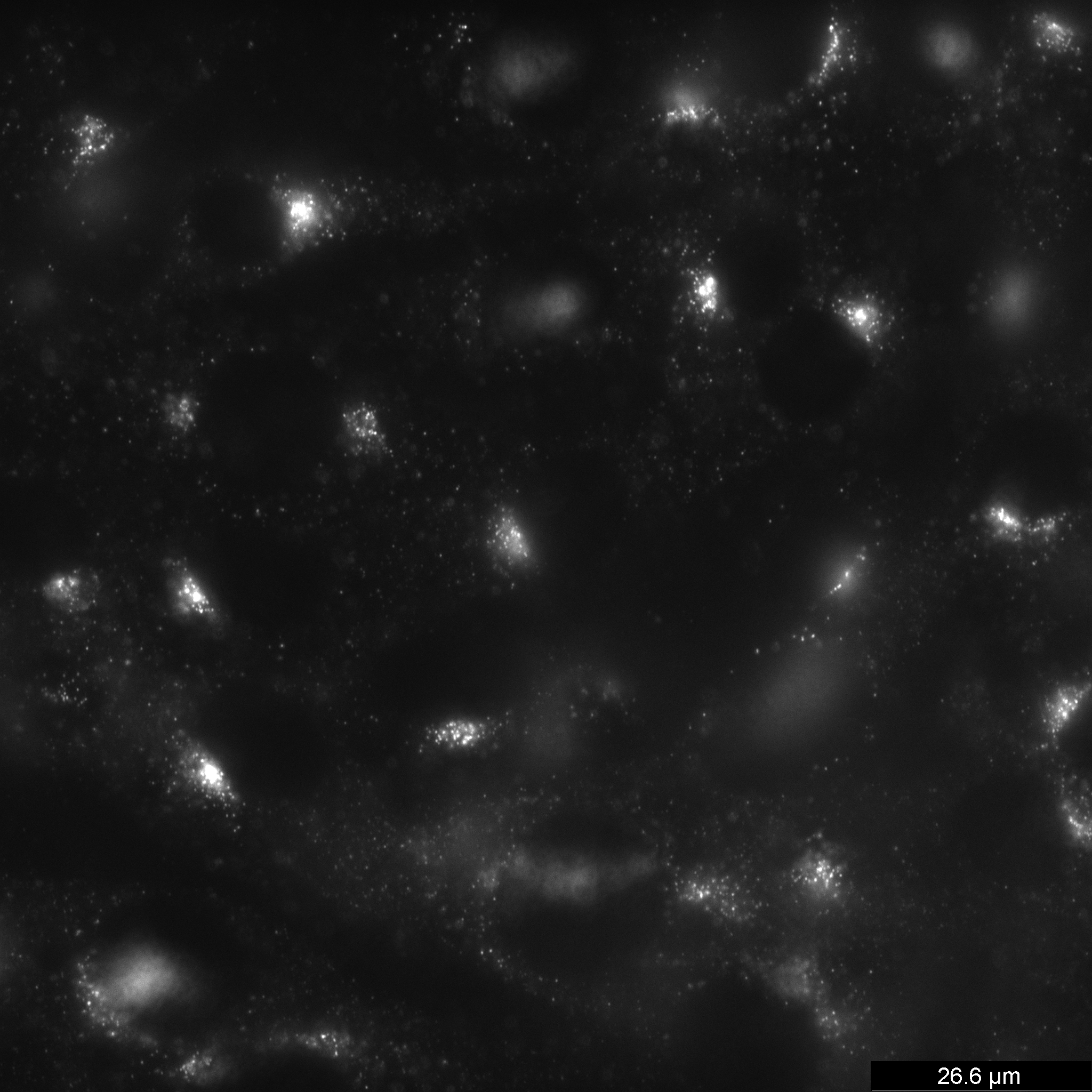

Supplement: Supplementary file 11 — Source Data for Figure 4 [file EMBR-24-e56870-s012.zip › Figure 4/4E/DEX+SR9011 32 h.tif]

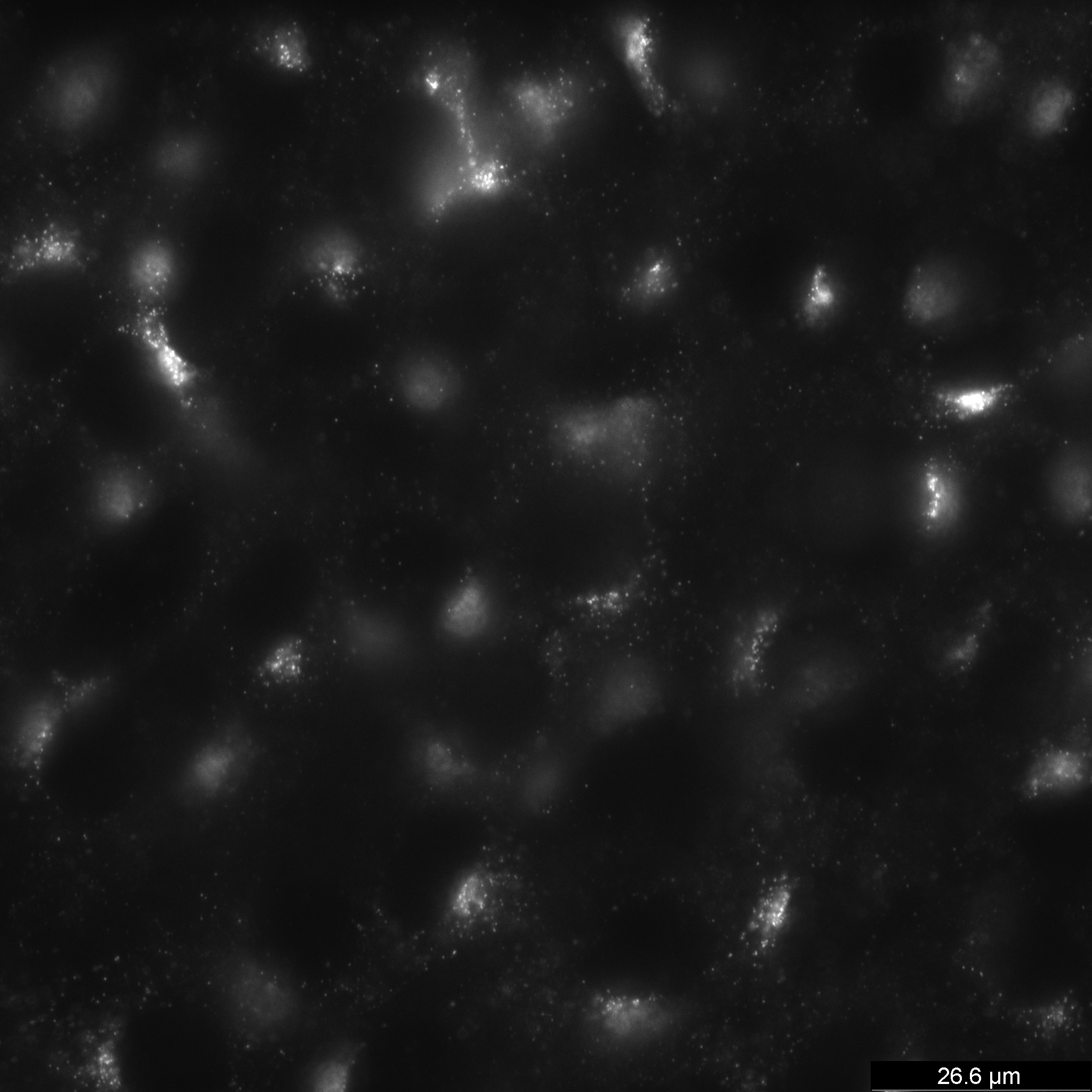

Supplement: Supplementary file 11 — Source Data for Figure 4 [file EMBR-24-e56870-s012.zip › Figure 4/4E/DEX+SR9011 48 h.tif]

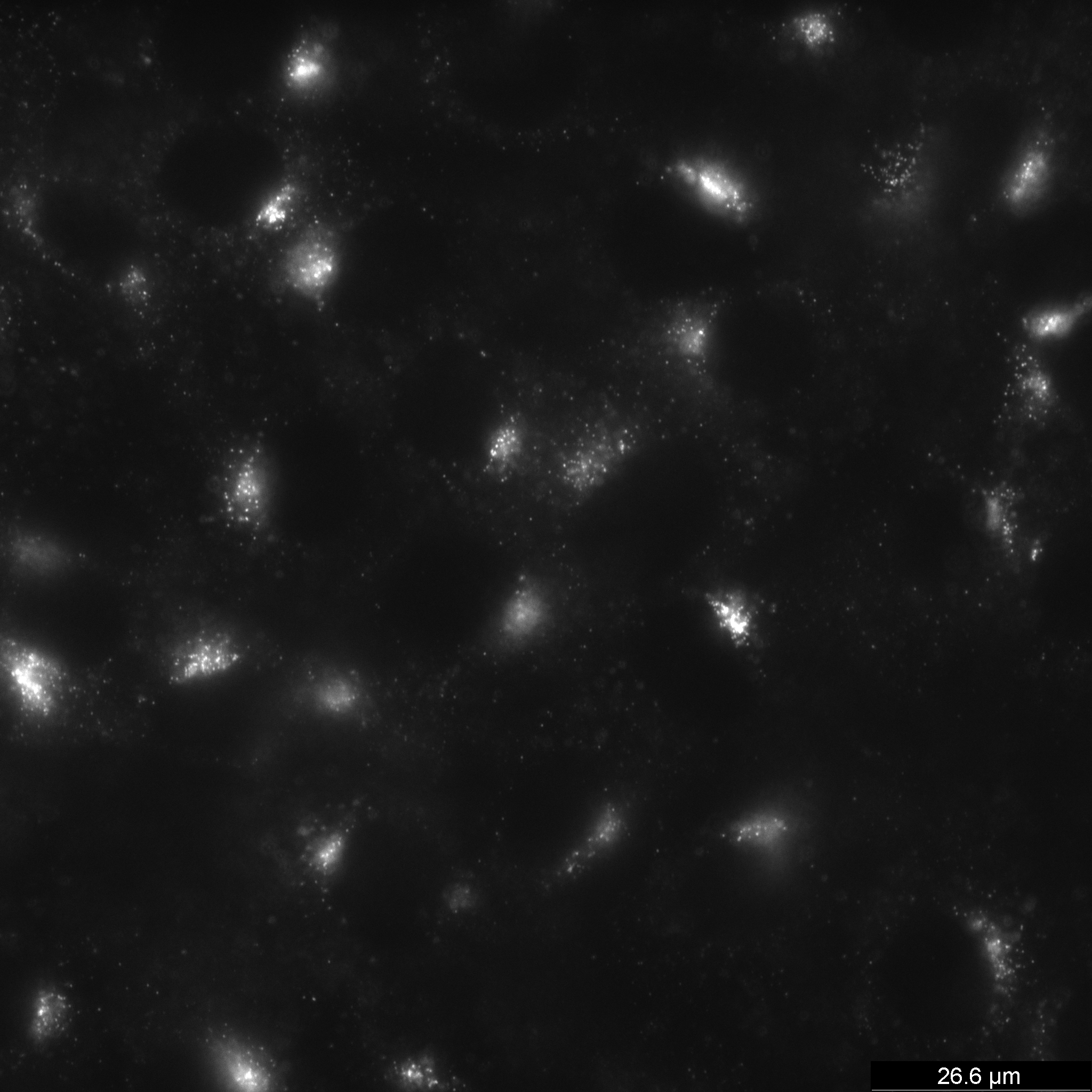

Supplement: Supplementary file 11 — Source Data for Figure 4 [file EMBR-24-e56870-s012.zip › Figure 4/4E/DEX+SR9011 44 h.tif]

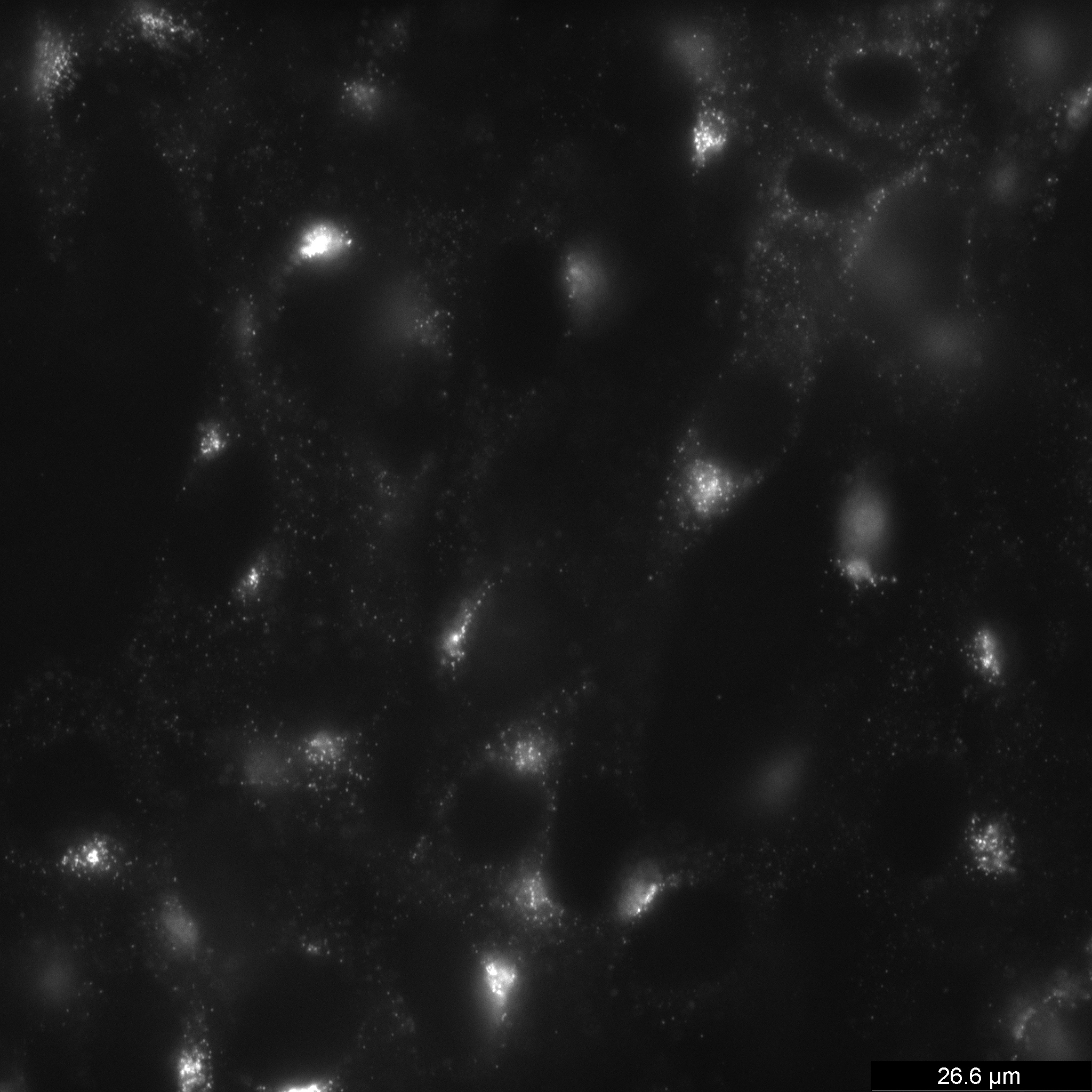

Supplement: Supplementary file 11 — Source Data for Figure 4 [file EMBR-24-e56870-s012.zip › Figure 4/4E/DEX 24 h.tif]

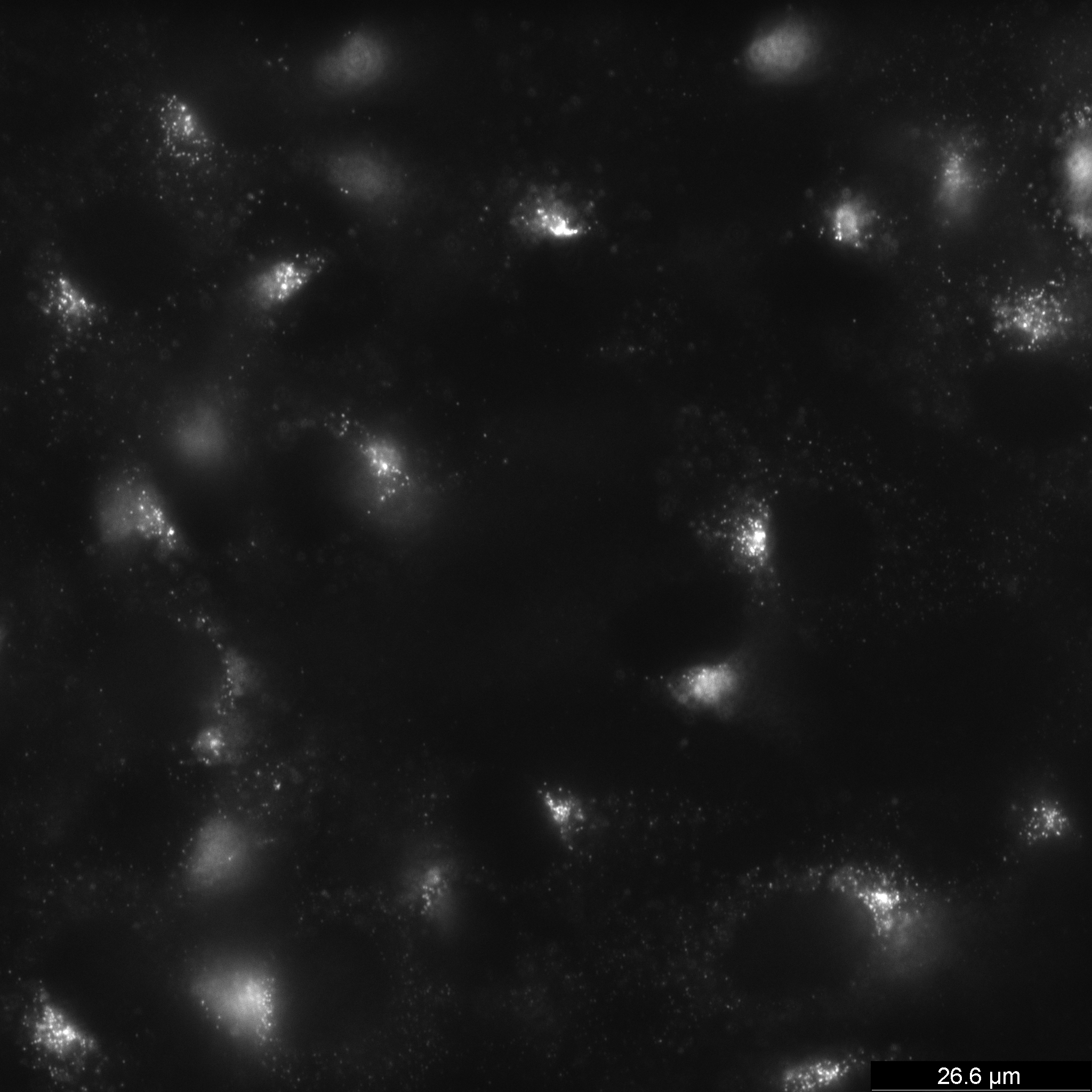

Supplement: Supplementary file 11 — Source Data for Figure 4 [file EMBR-24-e56870-s012.zip › Figure 4/4E/DEX+SR9011 40 h.tif]

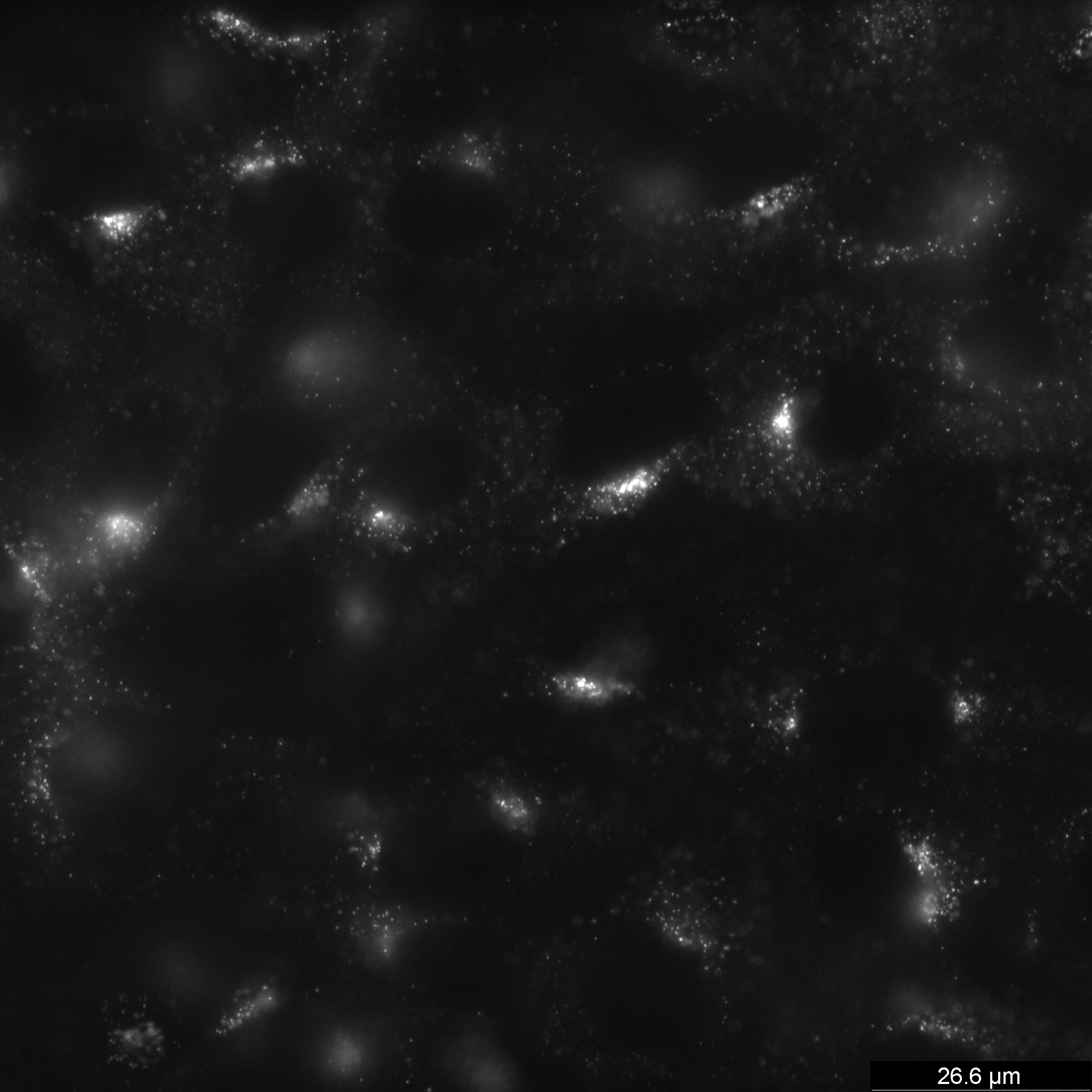

Supplement: Supplementary file 11 — Source Data for Figure 4 [file EMBR-24-e56870-s012.zip › Figure 4/4E/DEX 40 h.tif]

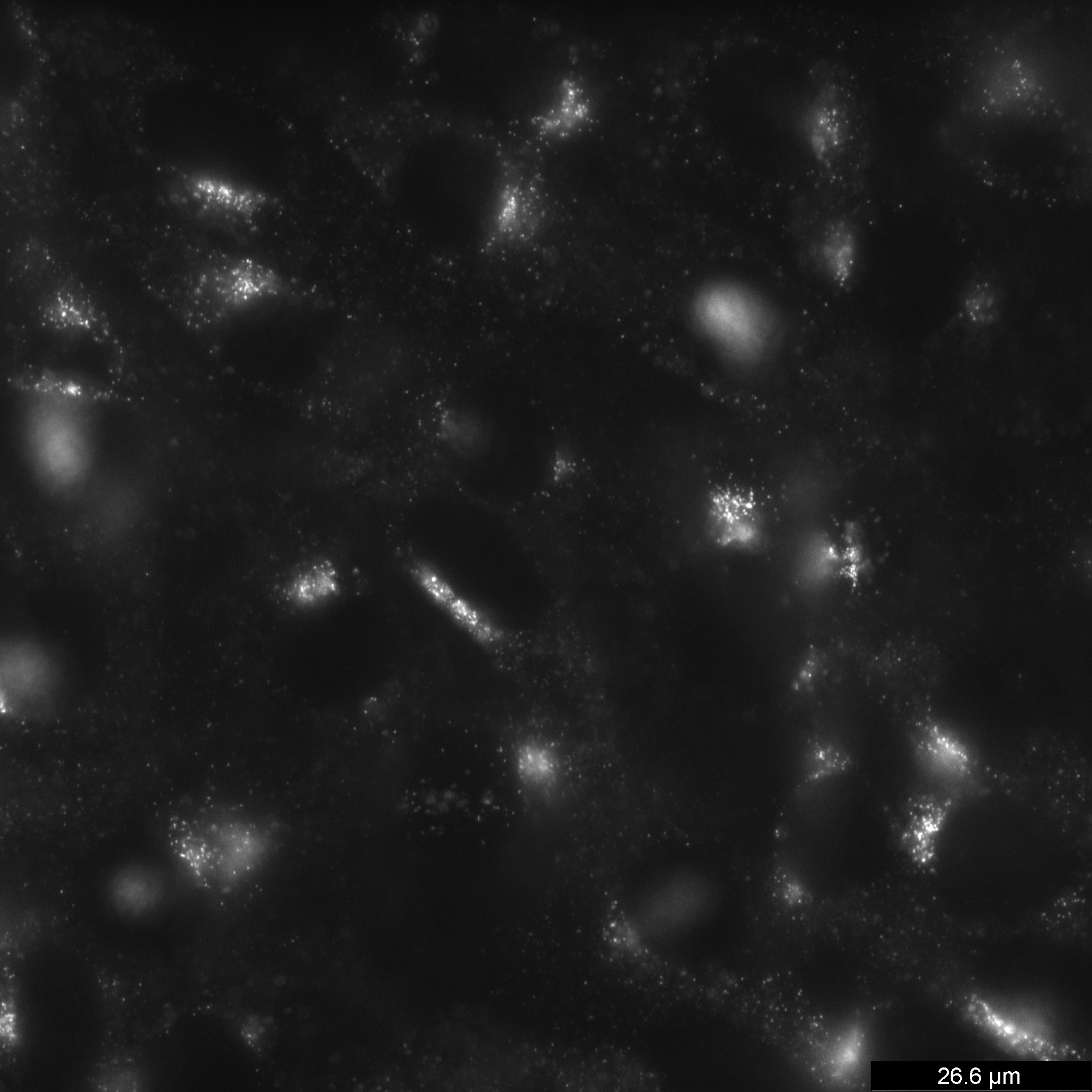

Supplement: Supplementary file 11 — Source Data for Figure 4 [file EMBR-24-e56870-s012.zip › Figure 4/4E/DEX+SR9011 24 h.tif]

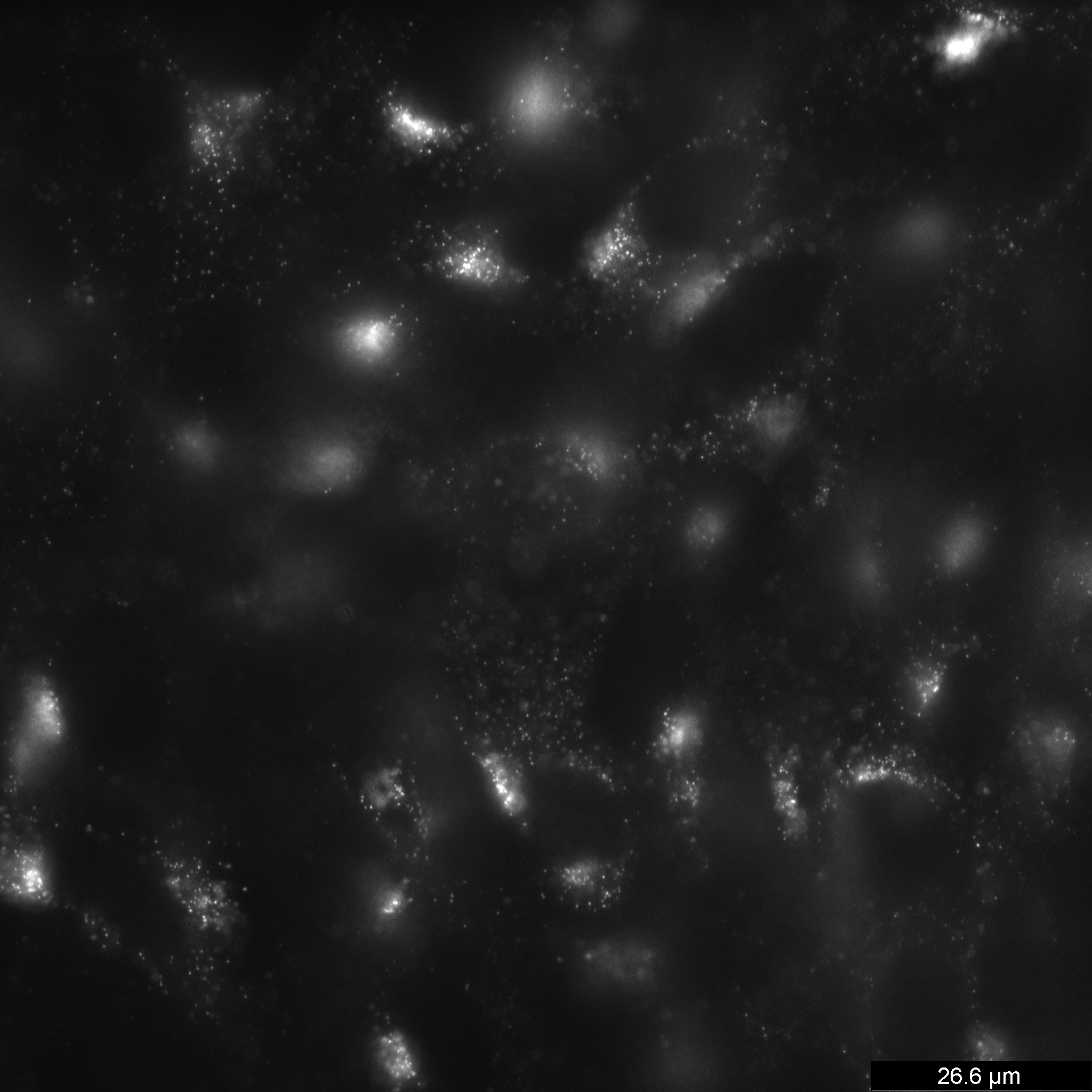

Supplement: Supplementary file 11 — Source Data for Figure 4 [file EMBR-24-e56870-s012.zip › Figure 4/4E/DEX 44 h.tif]

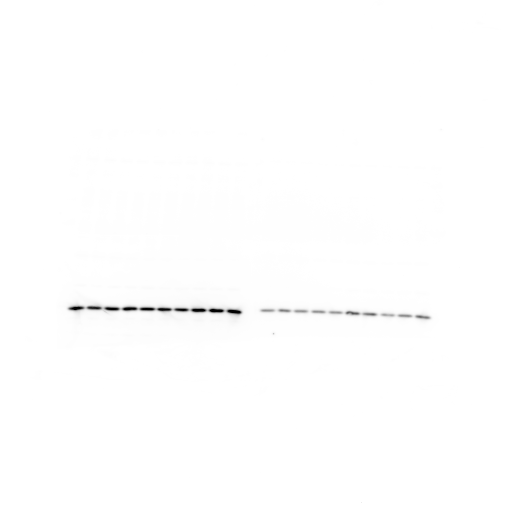

Supplement: Supplementary file 11 — Source Data for Figure 4 [file EMBR-24-e56870-s012.zip › Figure 4/4C/Gapdh raw data.tif]

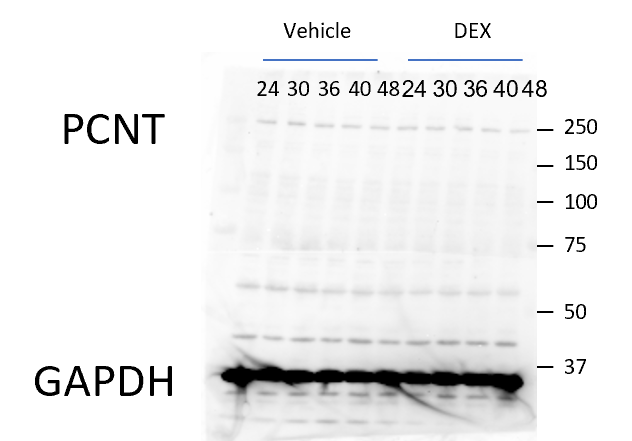

Supplement: Supplementary file 11 — Source Data for Figure 4 [file EMBR-24-e56870-s012.zip › Figure 4/4C/PCNT GAPDH.tif]

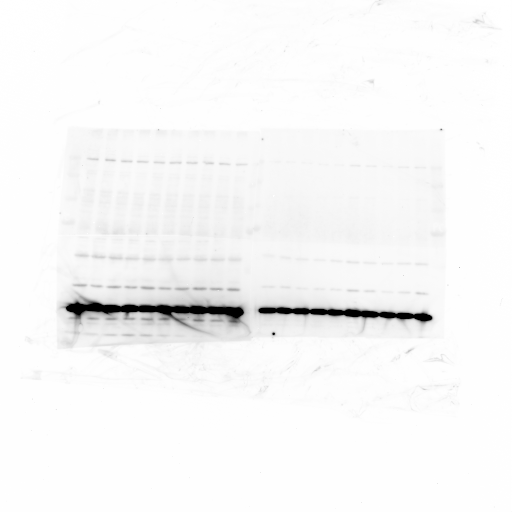

Supplement: Supplementary file 11 — Source Data for Figure 4 [file EMBR-24-e56870-s012.zip › Figure 4/4C/PCNT GAPDH raw data.tif]

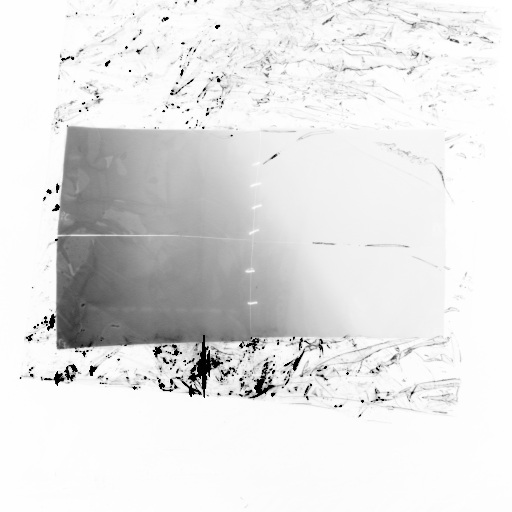

Supplement: Supplementary file 11 — Source Data for Figure 4 [file EMBR-24-e56870-s012.zip › Figure 4/4C/marker.tif]

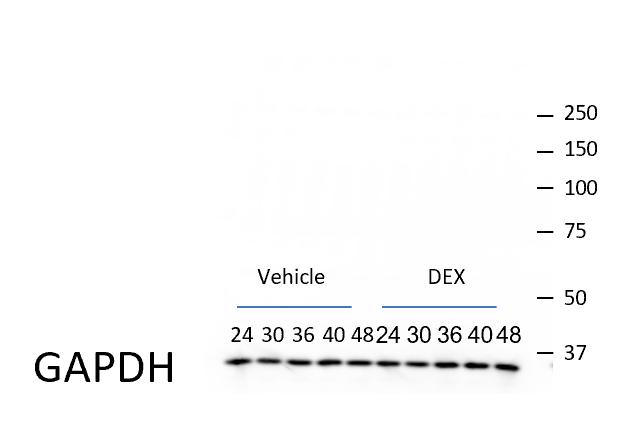

Supplement: Supplementary file 11 — Source Data for Figure 4 [file EMBR-24-e56870-s012.zip › Figure 4/4C/GAPDH.tif]

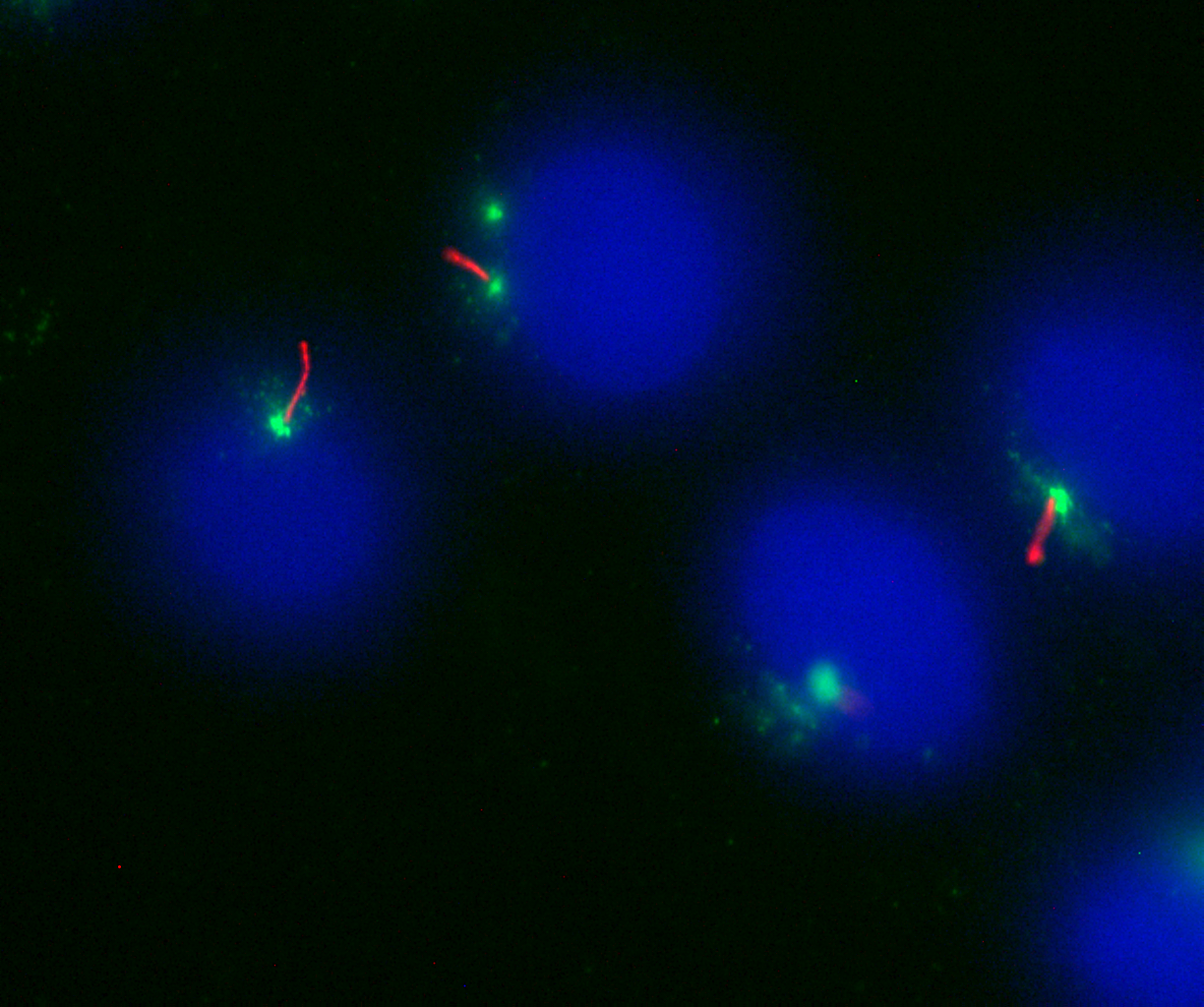

Supplement: Supplementary file 11 — Source Data for Figure 4 [file EMBR-24-e56870-s012.zip › Figure 4/4A/42 h.tif]

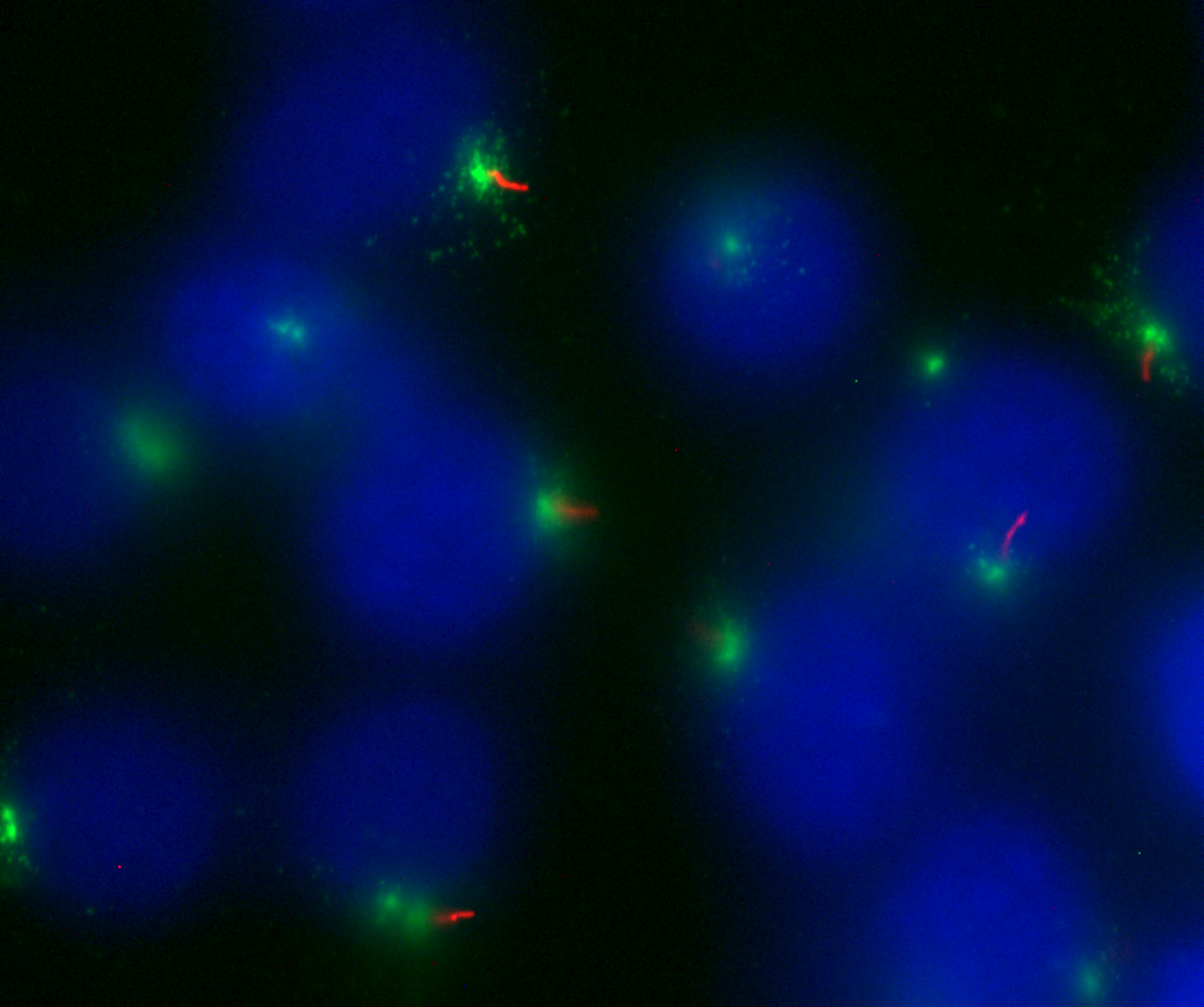

Supplement: Supplementary file 11 — Source Data for Figure 4 [file EMBR-24-e56870-s012.zip › Figure 4/4A/30 h.tif]

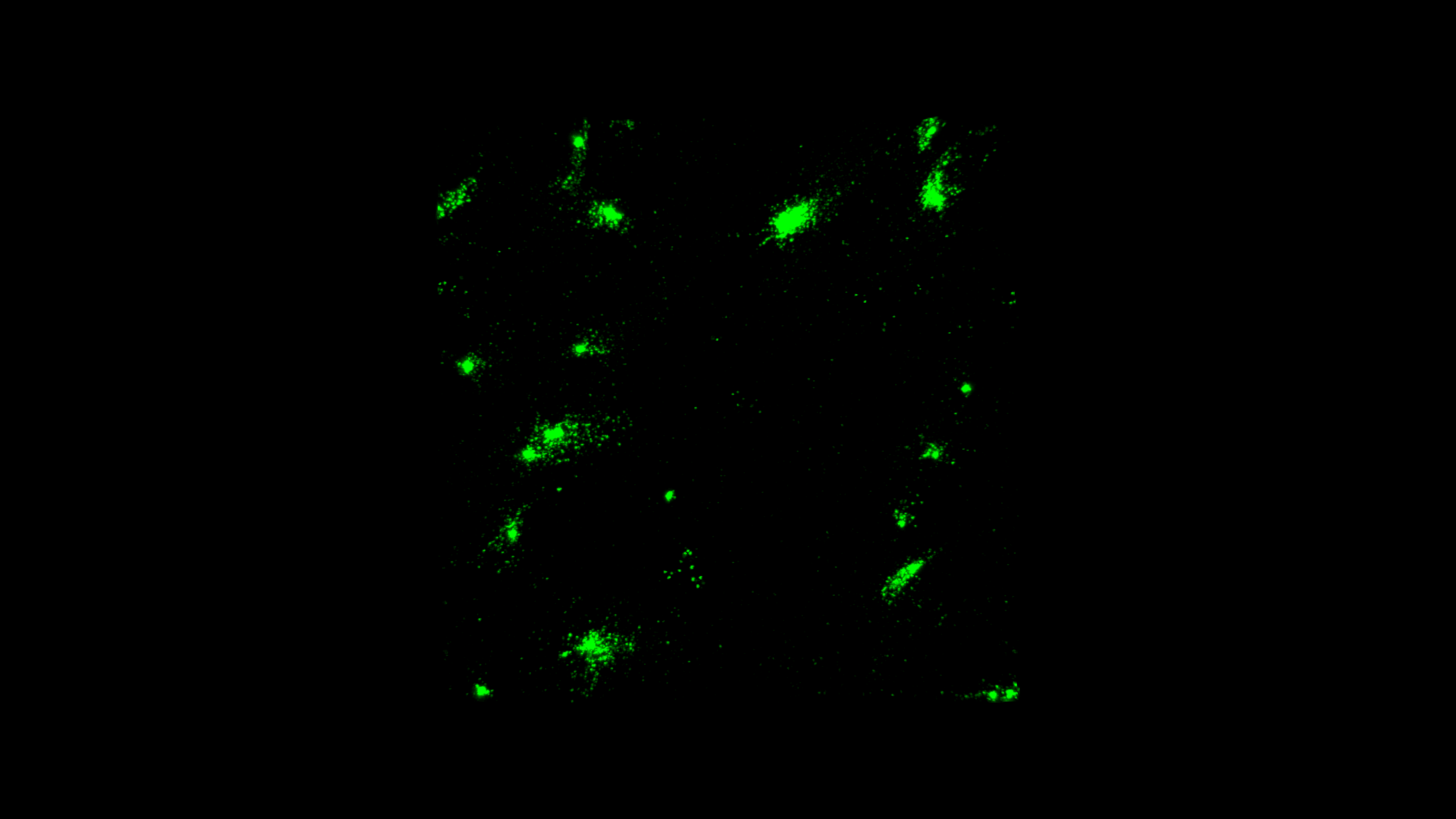

Supplement: Supplementary file 11 — Source Data for Figure 4 [file EMBR-24-e56870-s012.zip › Figure 4/4D/DEX/3.tif]

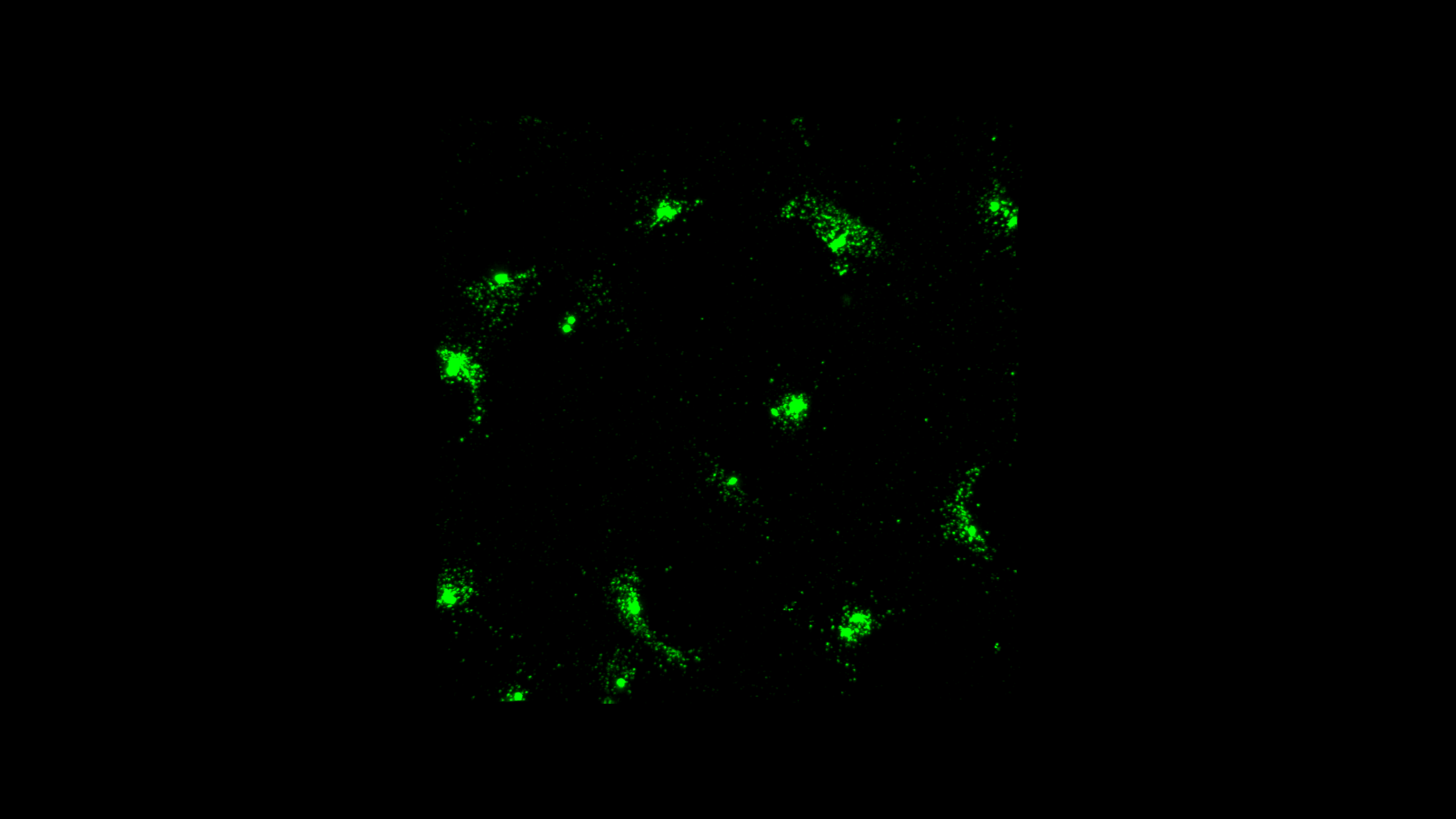

Supplement: Supplementary file 11 — Source Data for Figure 4 [file EMBR-24-e56870-s012.zip › Figure 4/4D/DEX/2.tif]

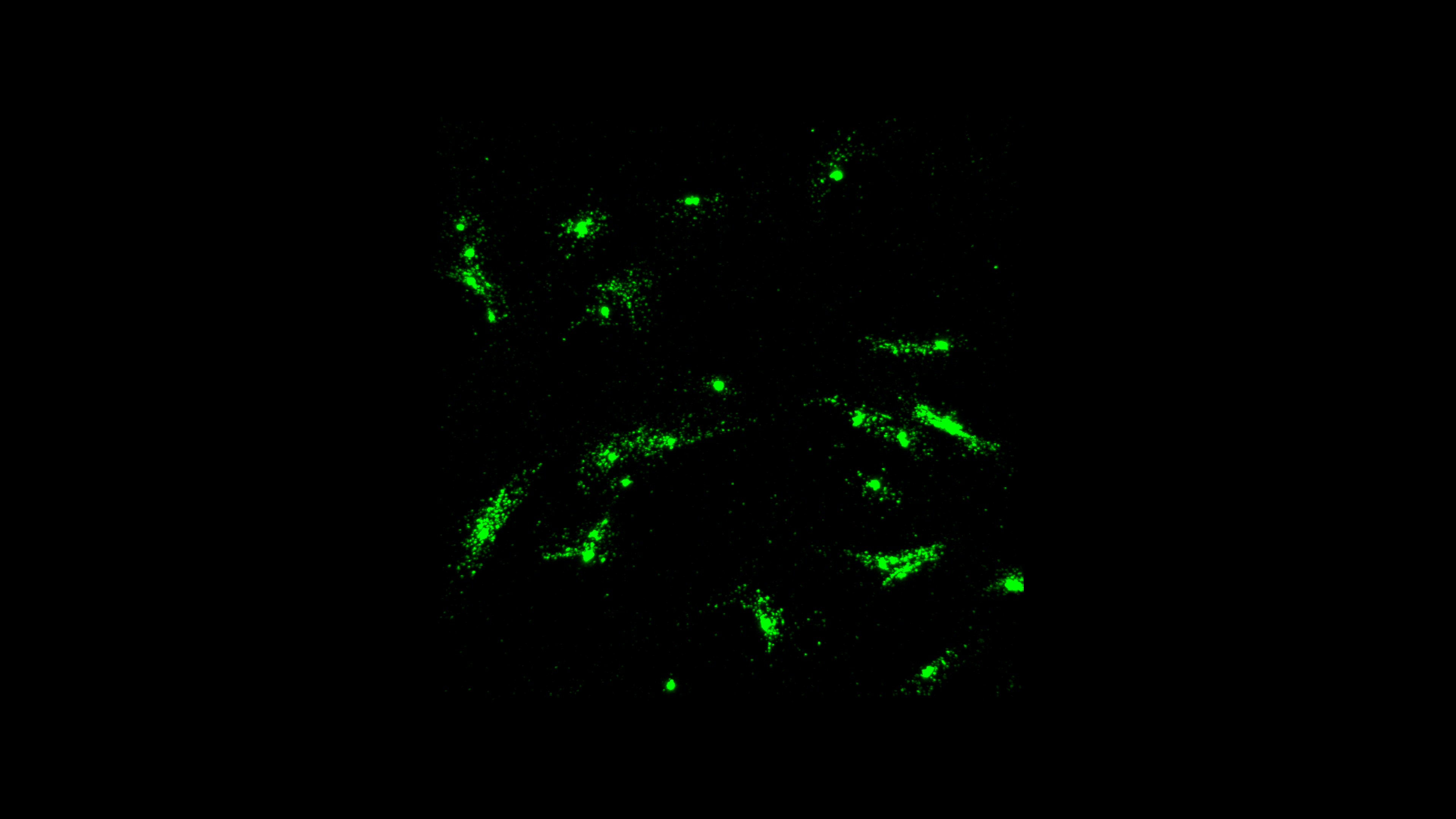

Supplement: Supplementary file 11 — Source Data for Figure 4 [file EMBR-24-e56870-s012.zip › Figure 4/4D/DEX/1.tif]

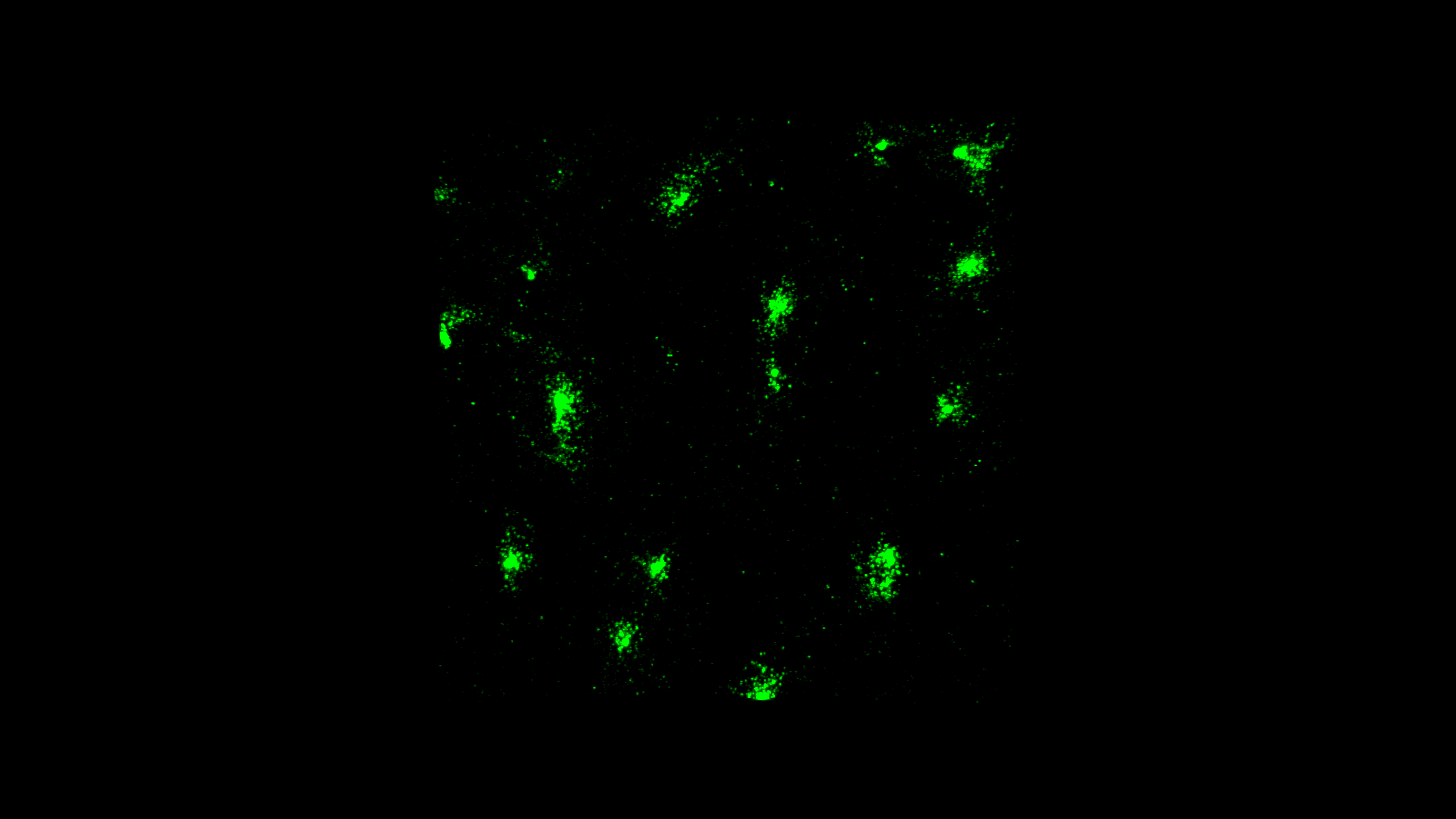

Supplement: Supplementary file 11 — Source Data for Figure 4 [file EMBR-24-e56870-s012.zip › Figure 4/4D/DEX/4.tif]

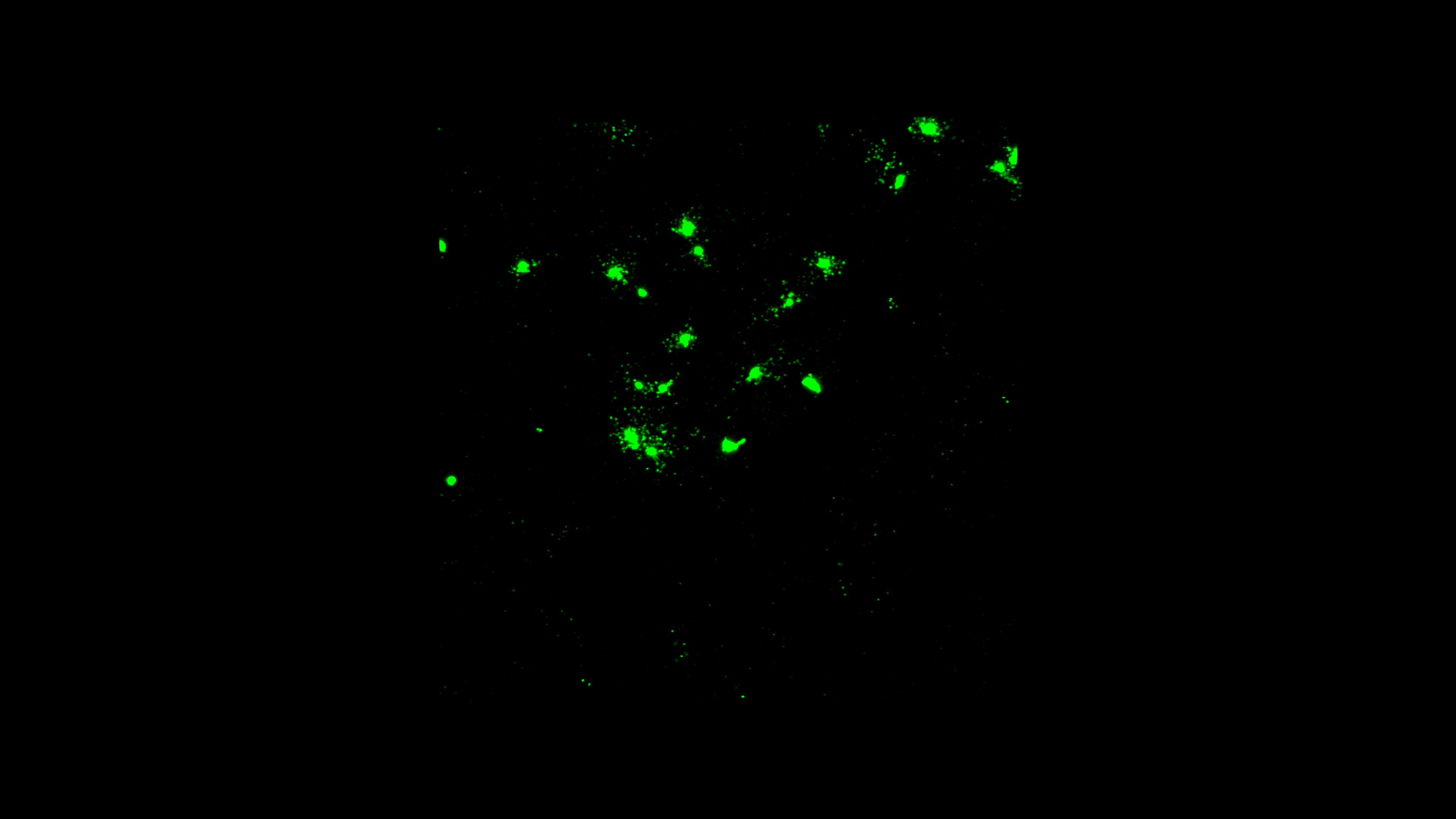

Supplement: Supplementary file 11 — Source Data for Figure 4 [file EMBR-24-e56870-s012.zip › Figure 4/4D/DEX+SR/3.tif]

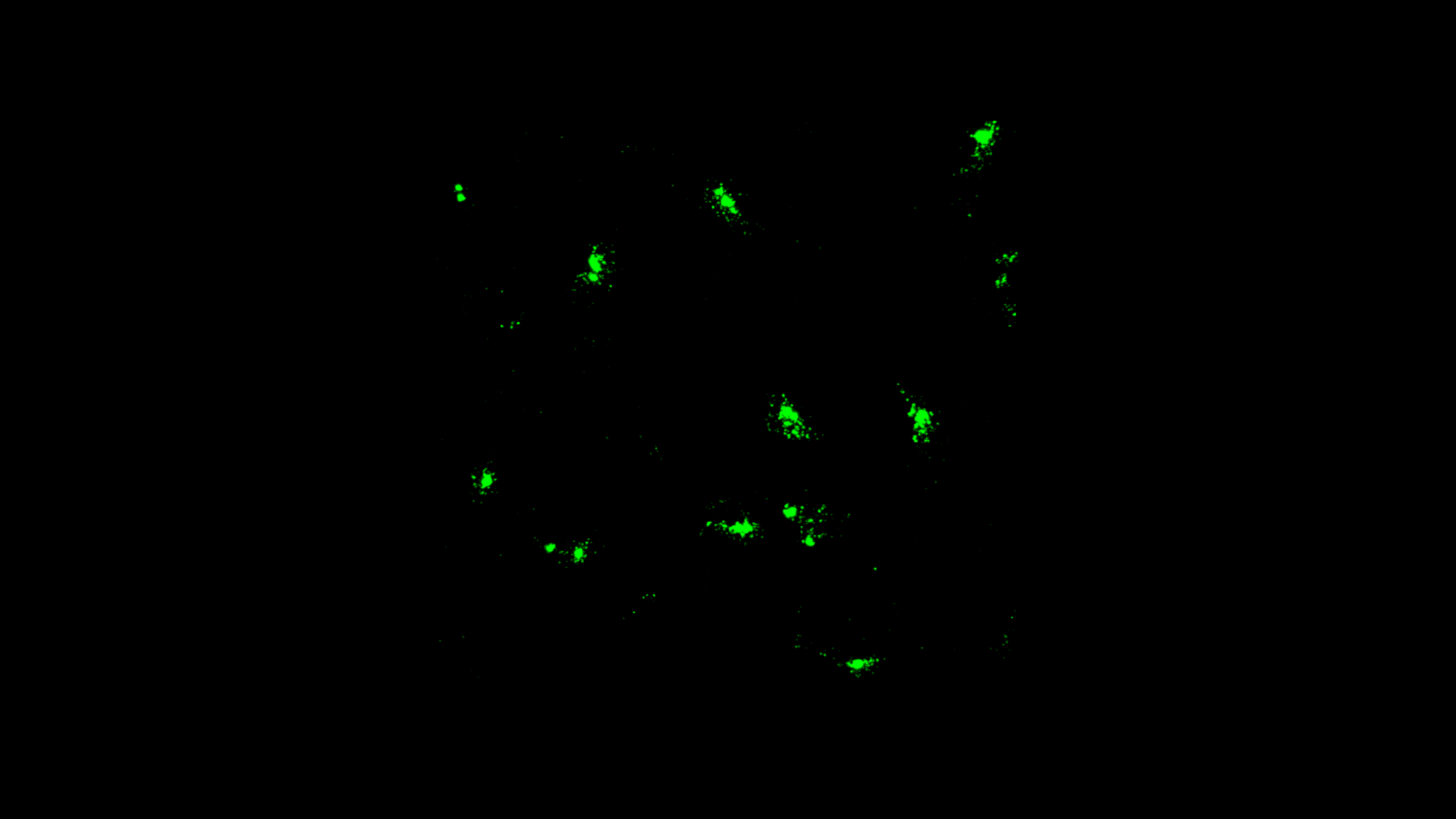

Supplement: Supplementary file 11 — Source Data for Figure 4 [file EMBR-24-e56870-s012.zip › Figure 4/4D/DEX+SR/2.tif]

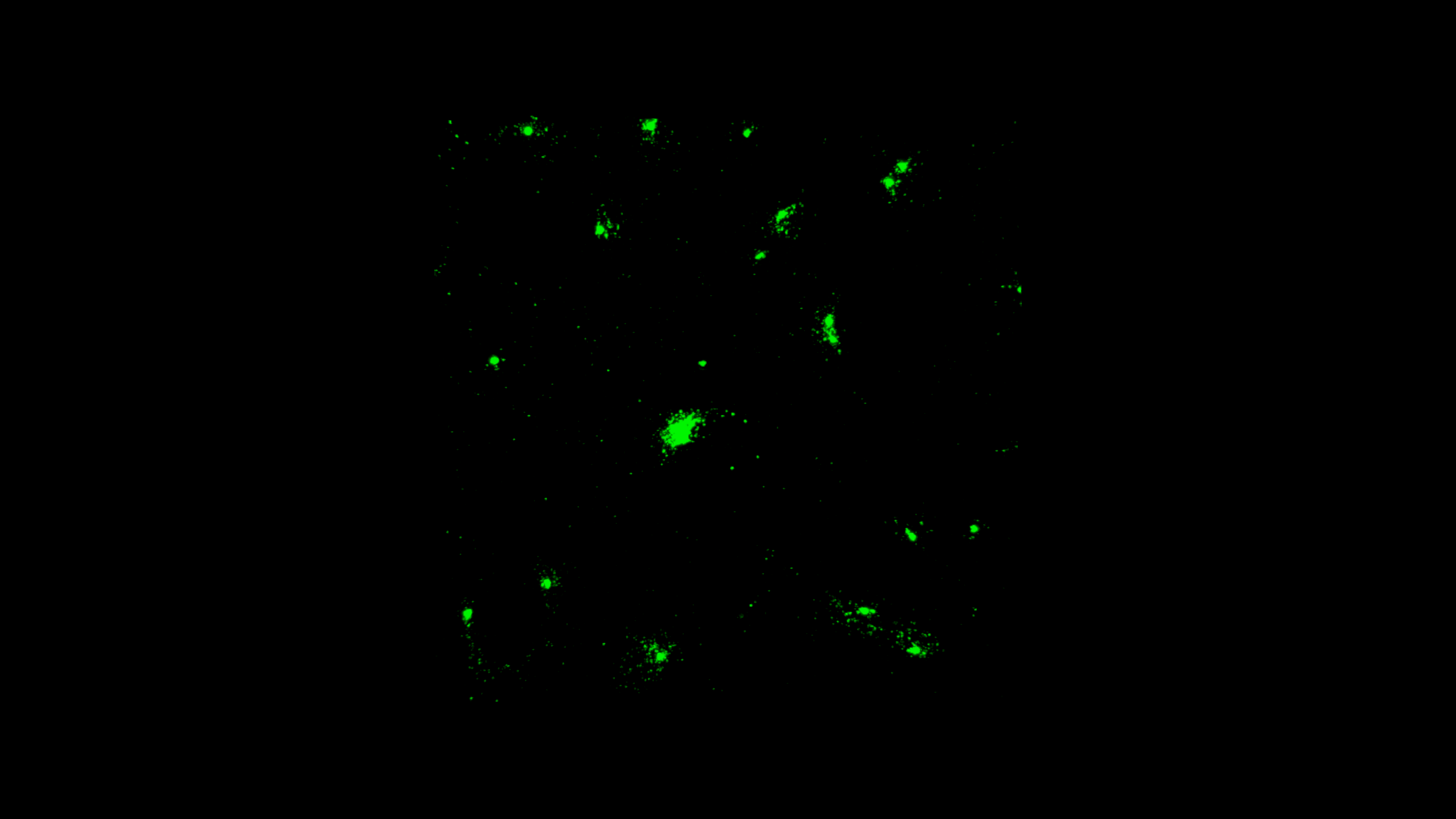

Supplement: Supplementary file 11 — Source Data for Figure 4 [file EMBR-24-e56870-s012.zip › Figure 4/4D/DEX+SR/1.tif]

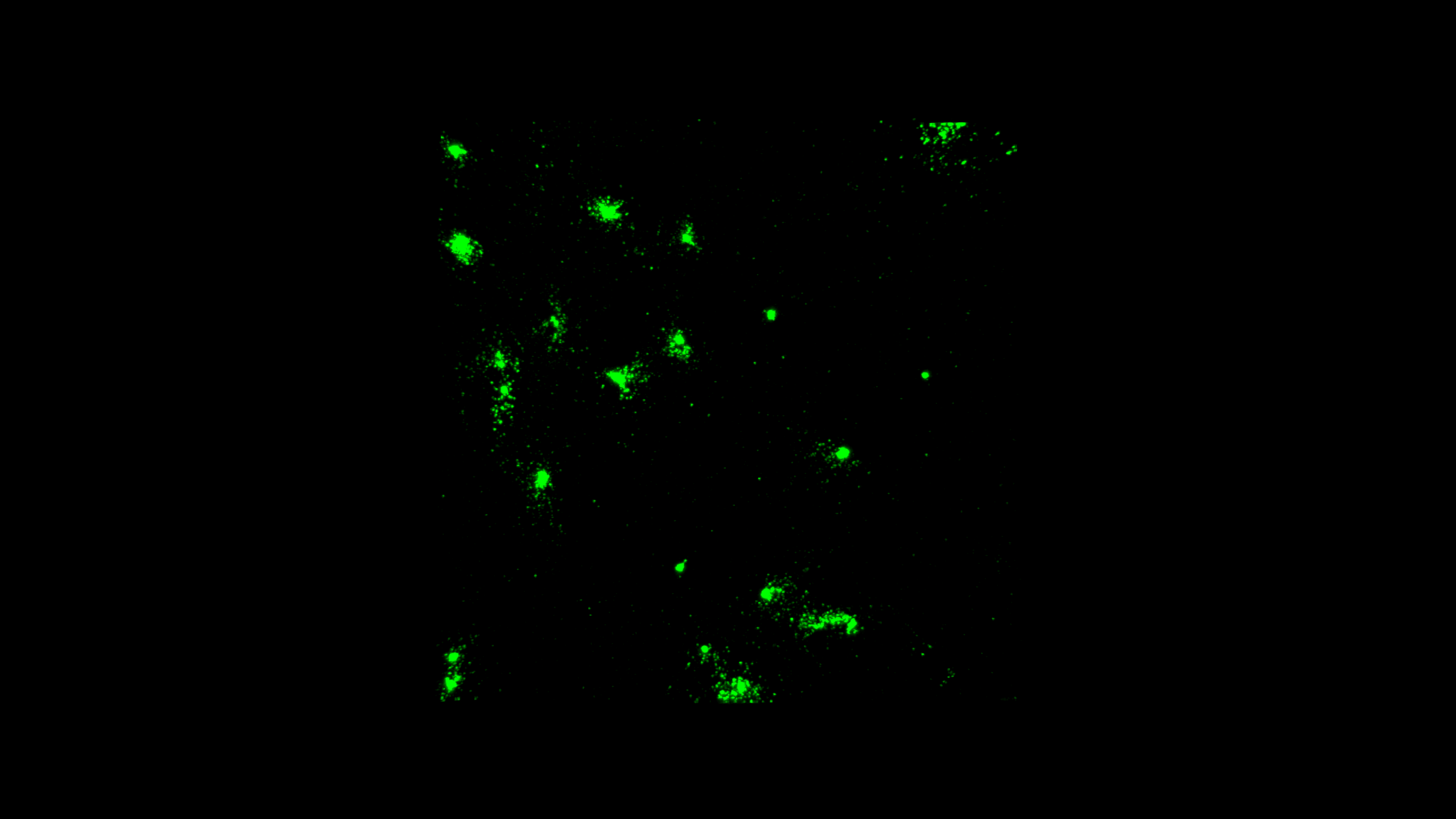

Supplement: Supplementary file 11 — Source Data for Figure 4 [file EMBR-24-e56870-s012.zip › Figure 4/4D/DEX+SR/4.tif]

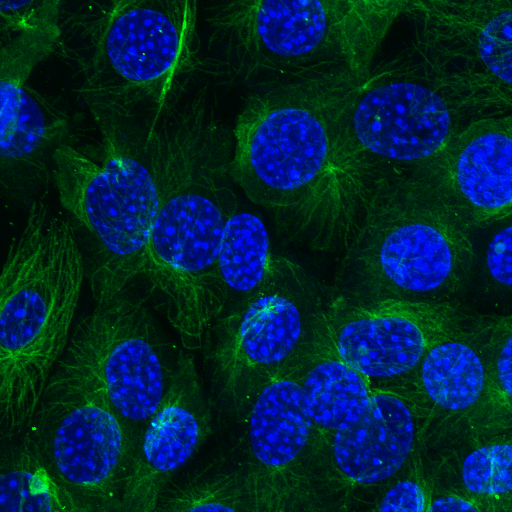

Supplement: Supplementary file 12 — Source Data for Figure 5 [file EMBR-24-e56870-s004.zip › Figure 5/5A/200 nM Colchicine.tif]

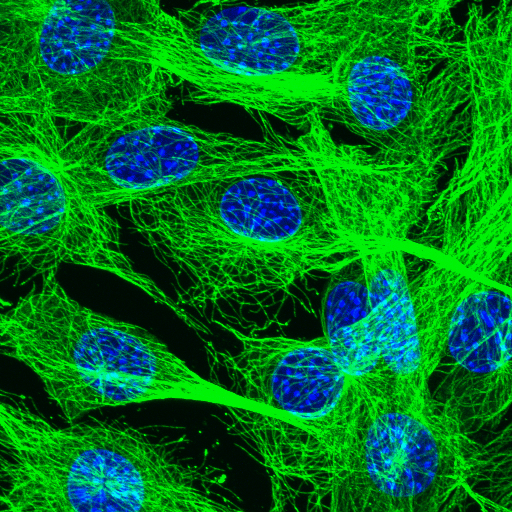

Supplement: Supplementary file 12 — Source Data for Figure 5 [file EMBR-24-e56870-s004.zip › Figure 5/5A/Control.tif]

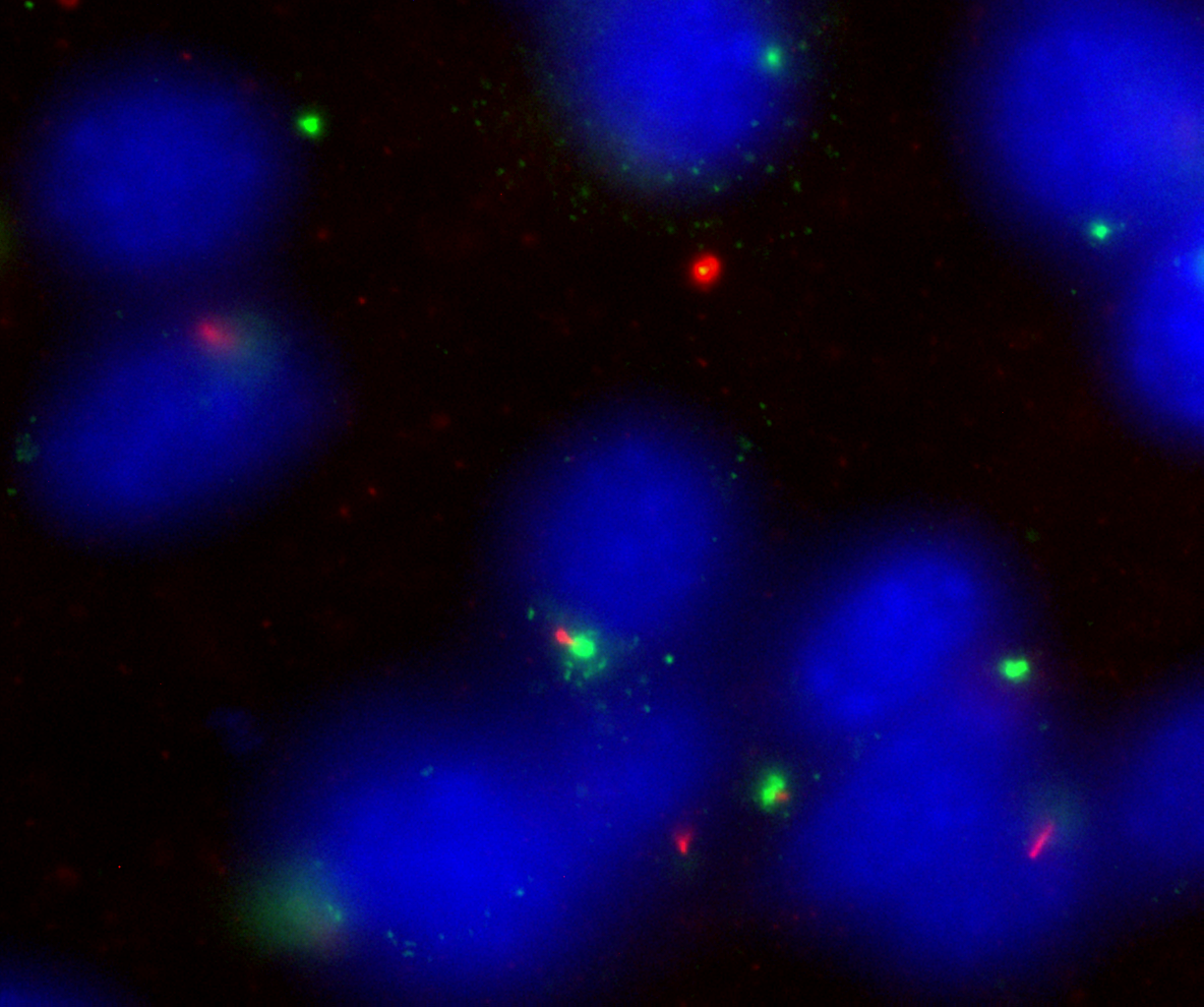

Supplement: Supplementary file 12 — Source Data for Figure 5 [file EMBR-24-e56870-s004.zip › Figure 5/5B/200 nM Colchicine.tif]

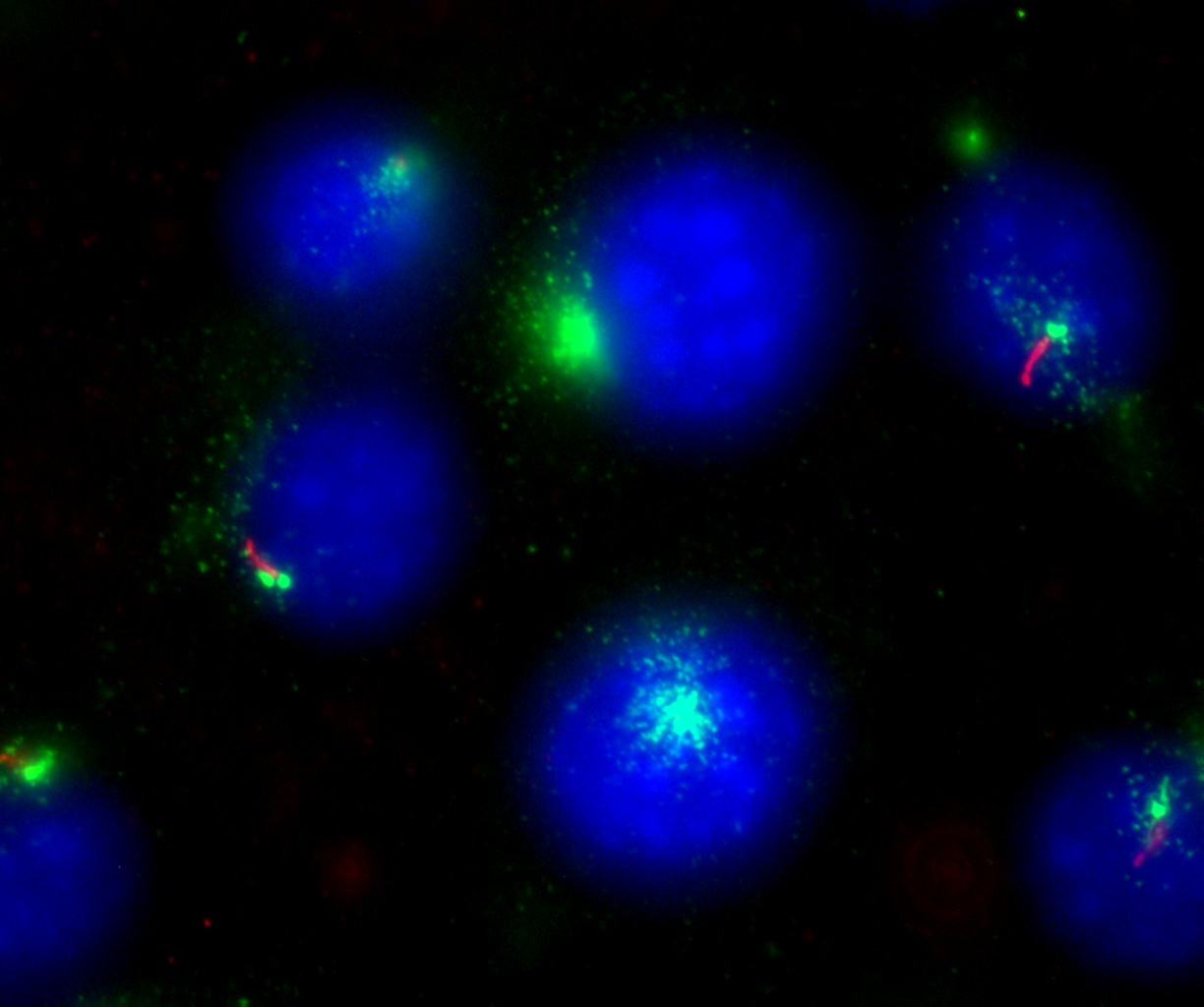

Supplement: Supplementary file 12 — Source Data for Figure 5 [file EMBR-24-e56870-s004.zip › Figure 5/5B/Control.tif]

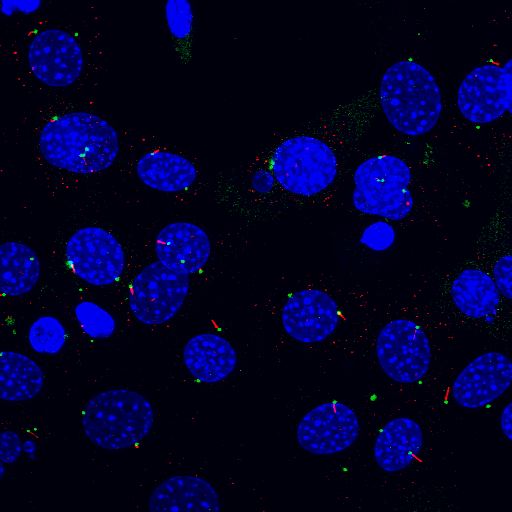

Supplement: Supplementary file 12 — Source Data for Figure 5 [file EMBR-24-e56870-s004.zip › Figure 5/5E/200 nM colchicine.tif]

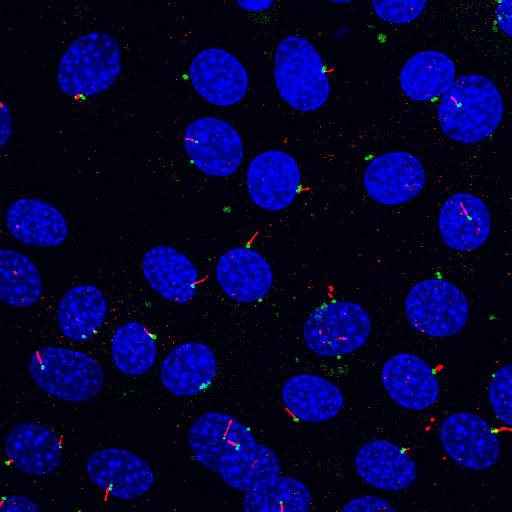

Supplement: Supplementary file 12 — Source Data for Figure 5 [file EMBR-24-e56870-s004.zip › Figure 5/5E/Control.tif]

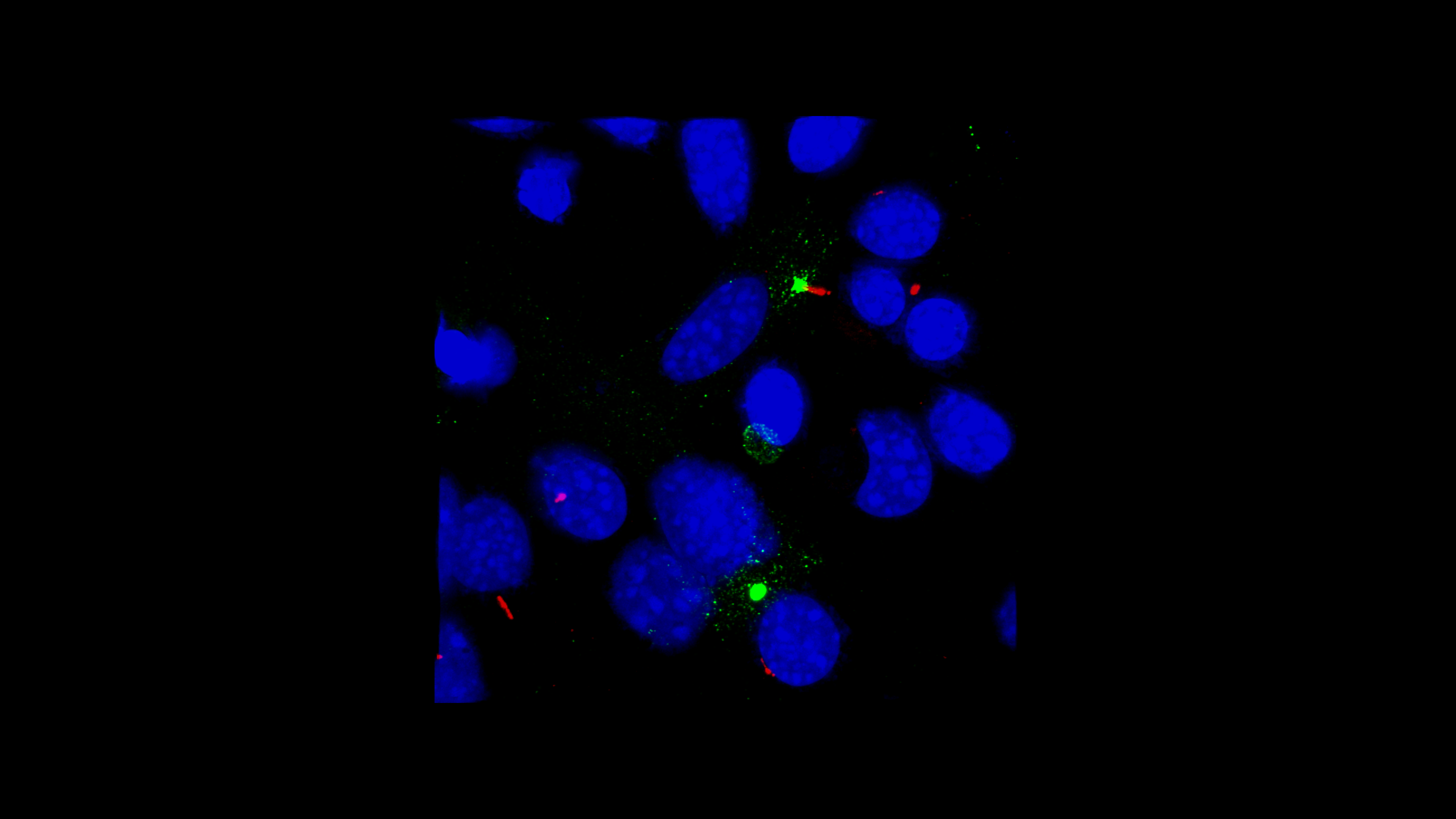

Supplement: Supplementary file 12 — Source Data for Figure 5 [file EMBR-24-e56870-s004.zip › Figure 5/5H/BICD2-FRB.tif]

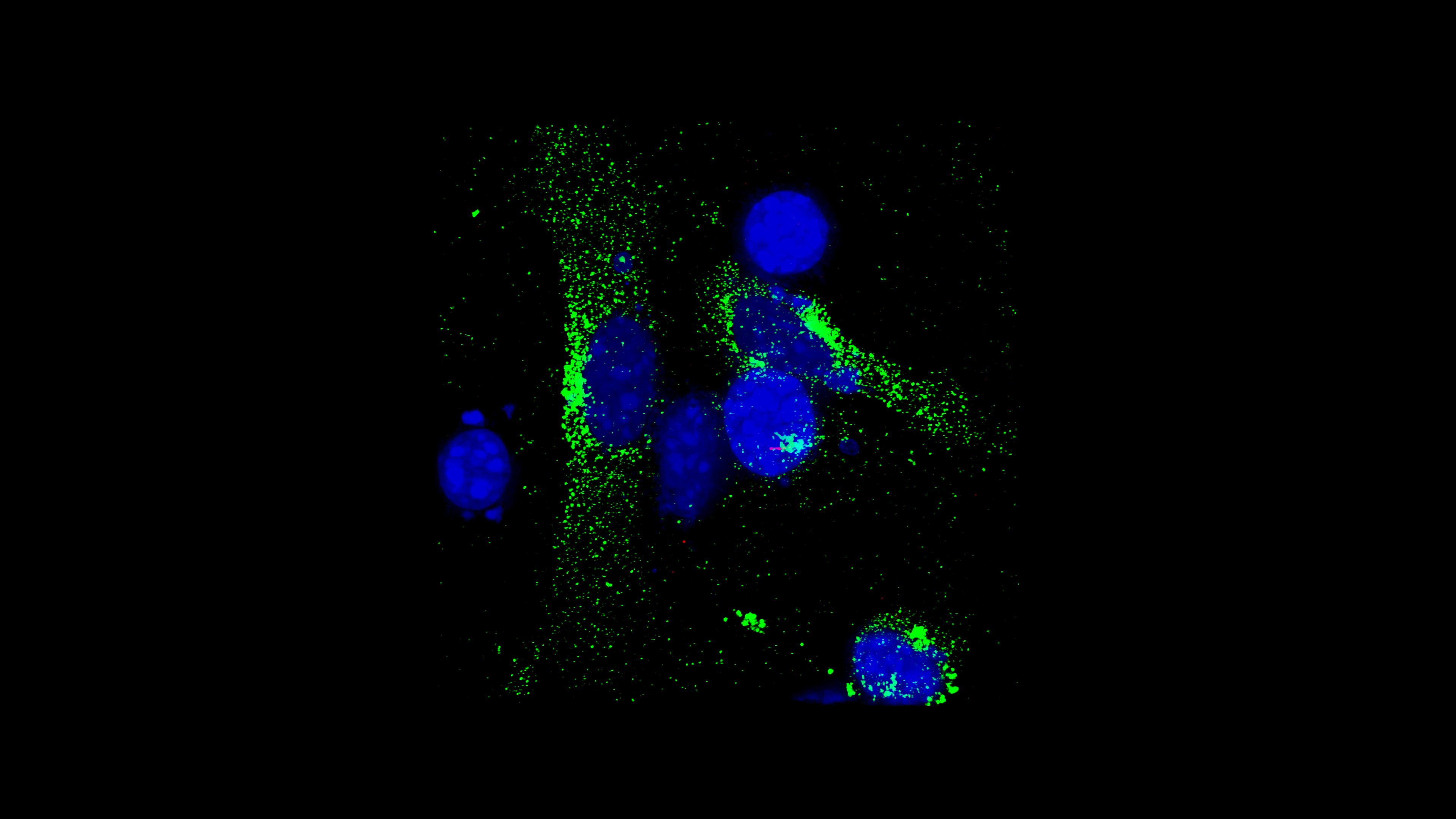

Supplement: Supplementary file 12 — Source Data for Figure 5 [file EMBR-24-e56870-s004.zip › Figure 5/5H/Control.tif]

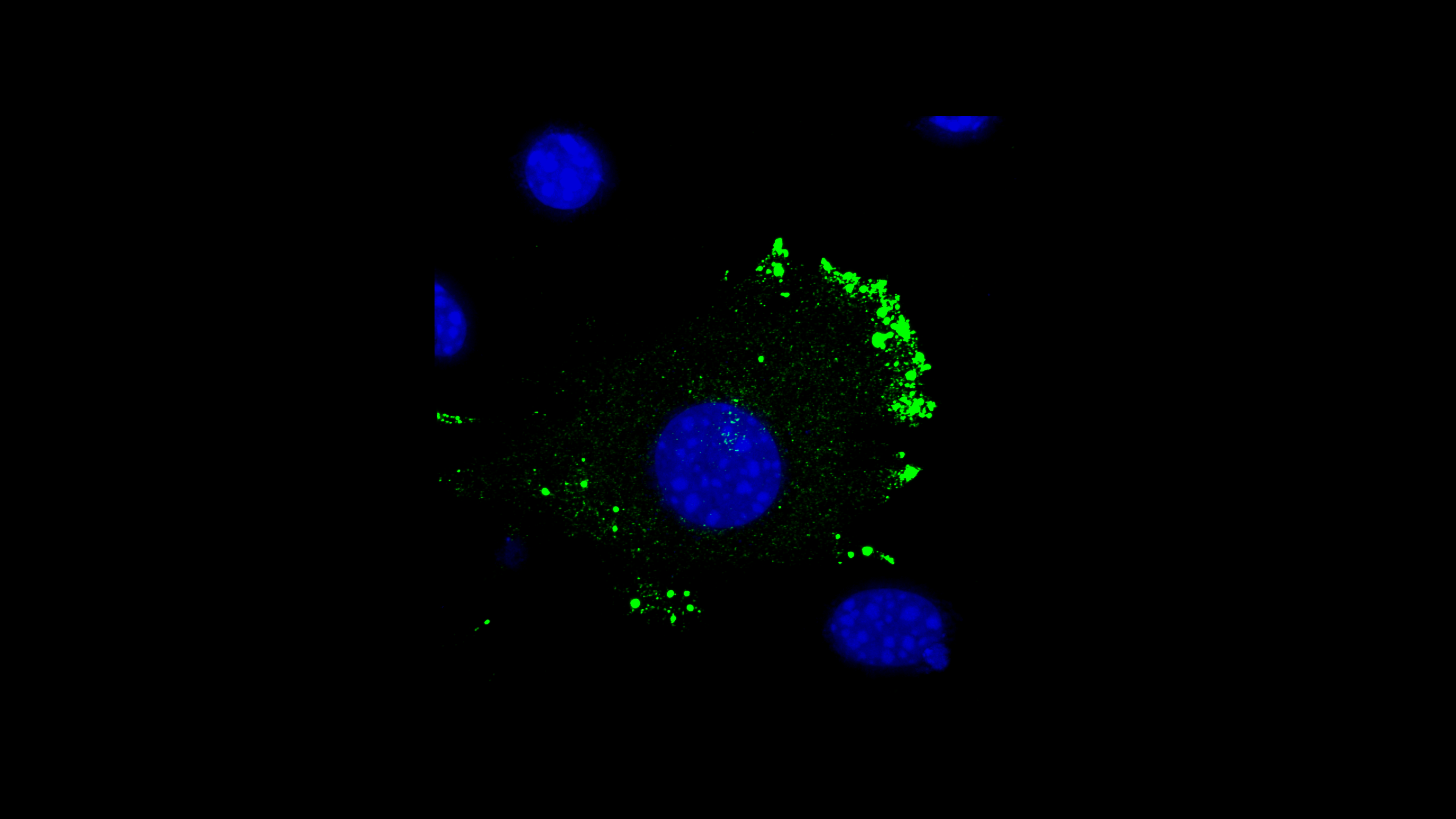

Supplement: Supplementary file 12 — Source Data for Figure 5 [file EMBR-24-e56870-s004.zip › Figure 5/5H/Kif5b-FRB.tif]

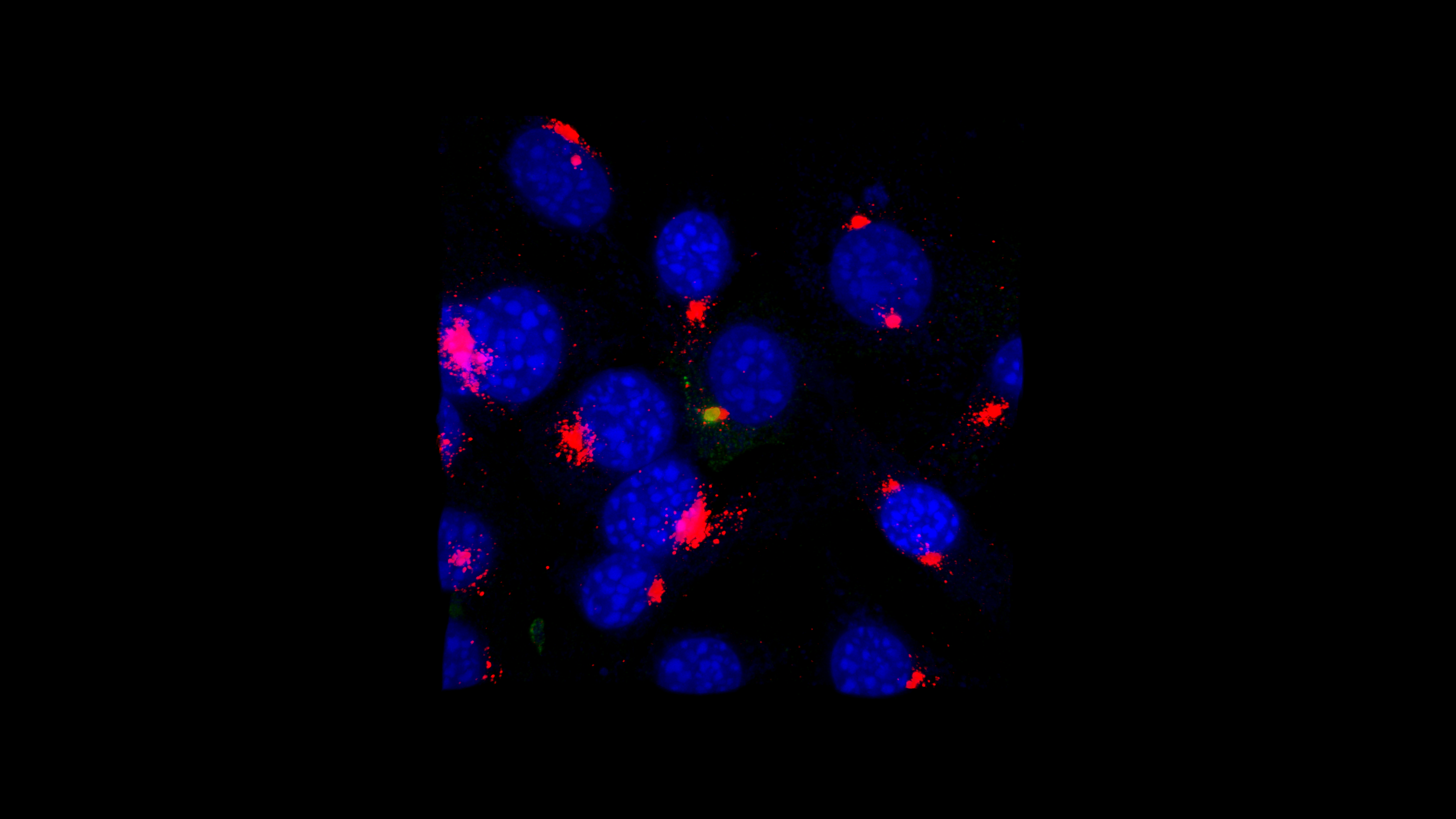

Supplement: Supplementary file 12 — Source Data for Figure 5 [file EMBR-24-e56870-s004.zip › Figure 5/5J/BICD2.tif]

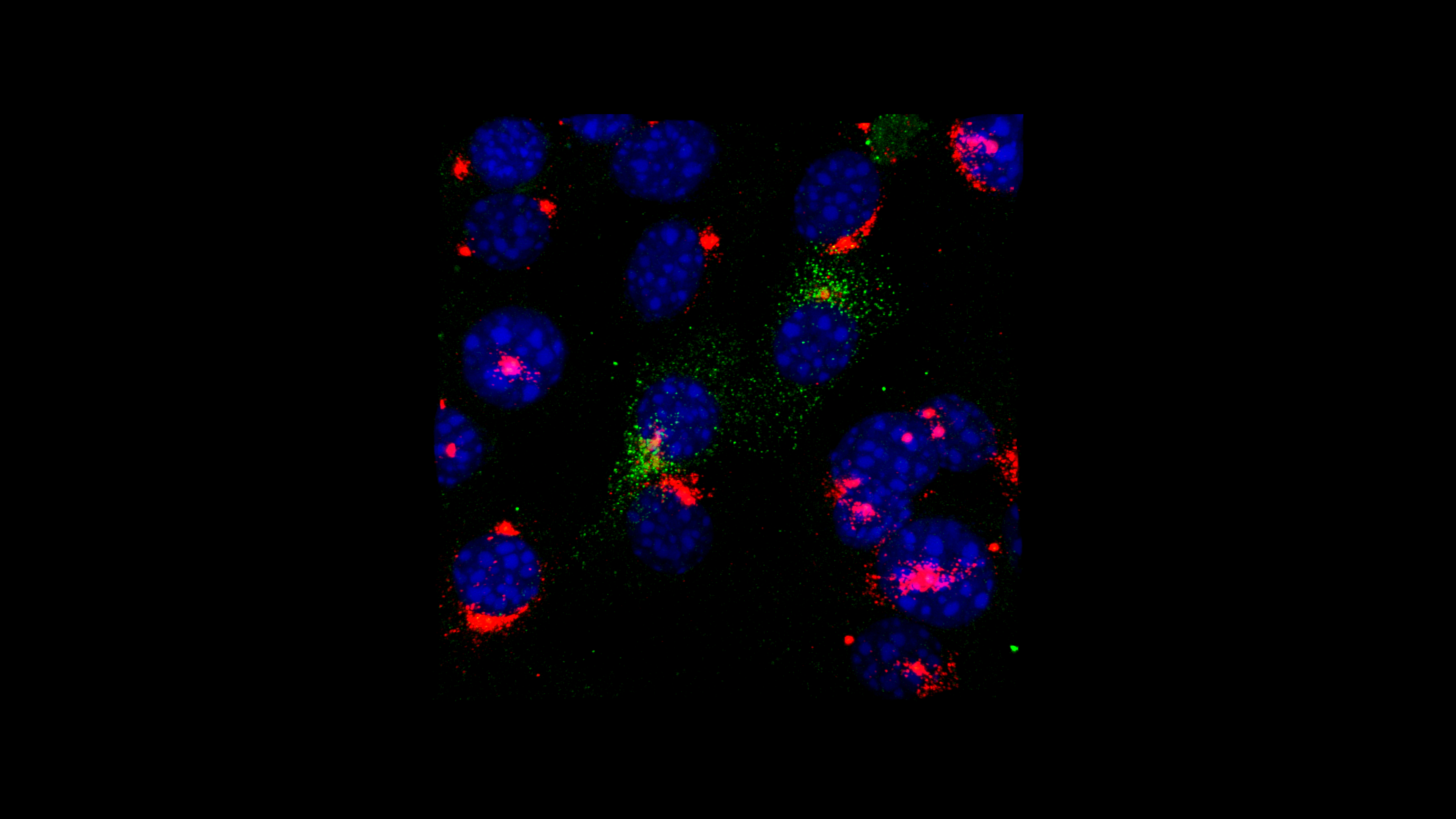

Supplement: Supplementary file 12 — Source Data for Figure 5 [file EMBR-24-e56870-s004.zip › Figure 5/5J/Control.tif]

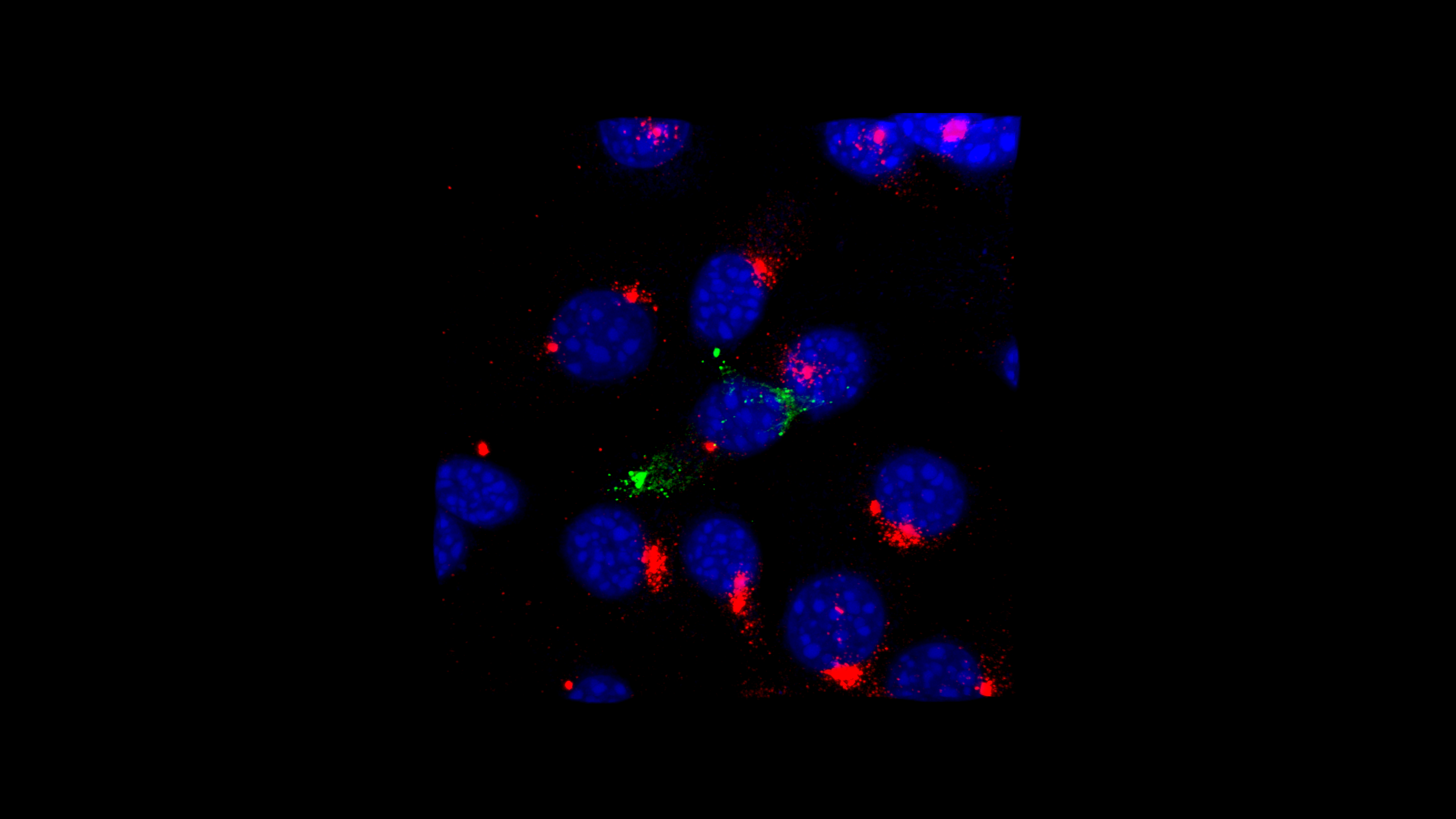

Supplement: Supplementary file 12 — Source Data for Figure 5 [file EMBR-24-e56870-s004.zip › Figure 5/5J/KIF5b.tif]

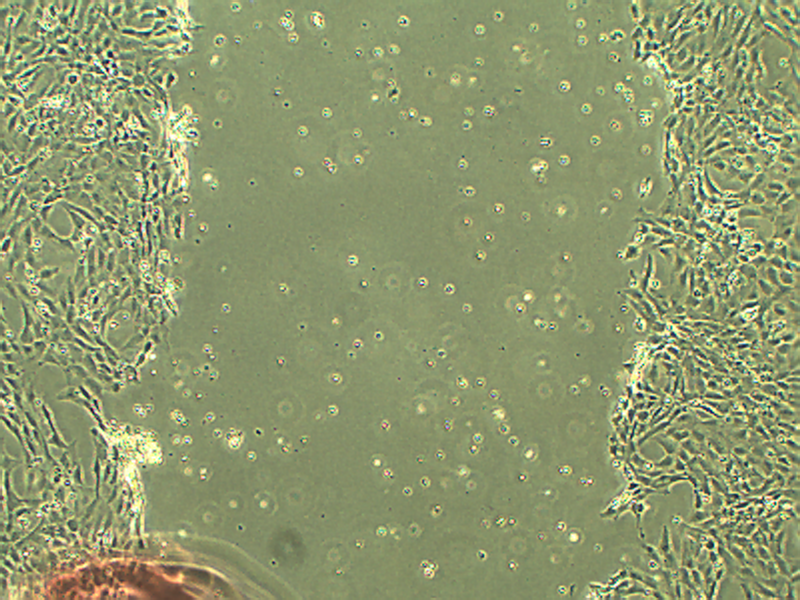

Supplement: Supplementary file 14 — Source Data for Figure 7 [file EMBR-24-e56870-s001.zip › Figure 7/7B/Kif3a KO 18 h 0 h.tif]

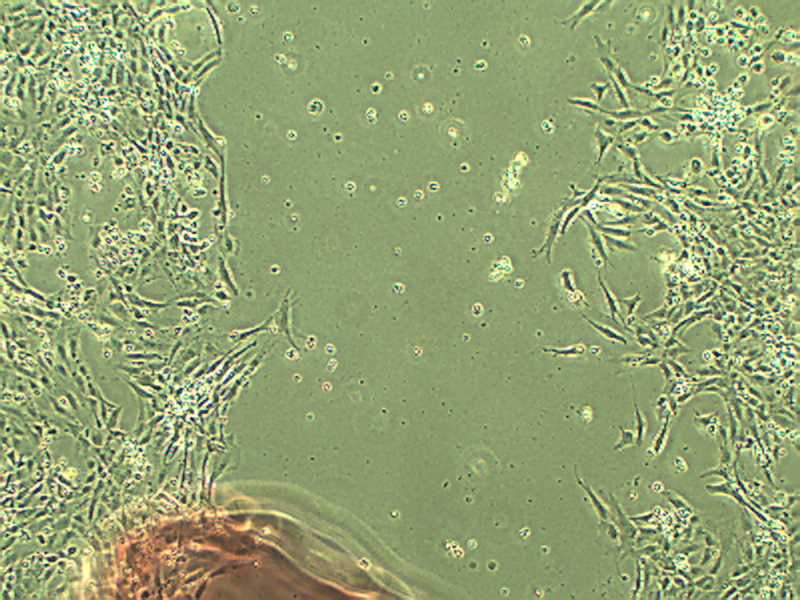

Supplement: Supplementary file 14 — Source Data for Figure 7 [file EMBR-24-e56870-s001.zip › Figure 7/7B/Kif3a KO 18 h 24 h.tif]

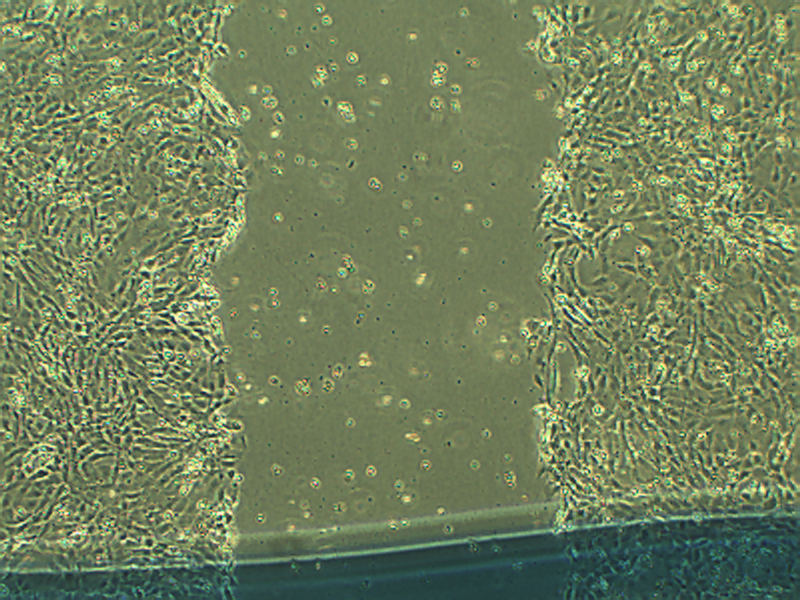

Supplement: Supplementary file 14 — Source Data for Figure 7 [file EMBR-24-e56870-s001.zip › Figure 7/7B/Kif3a KO 30 h 0 h.tif]

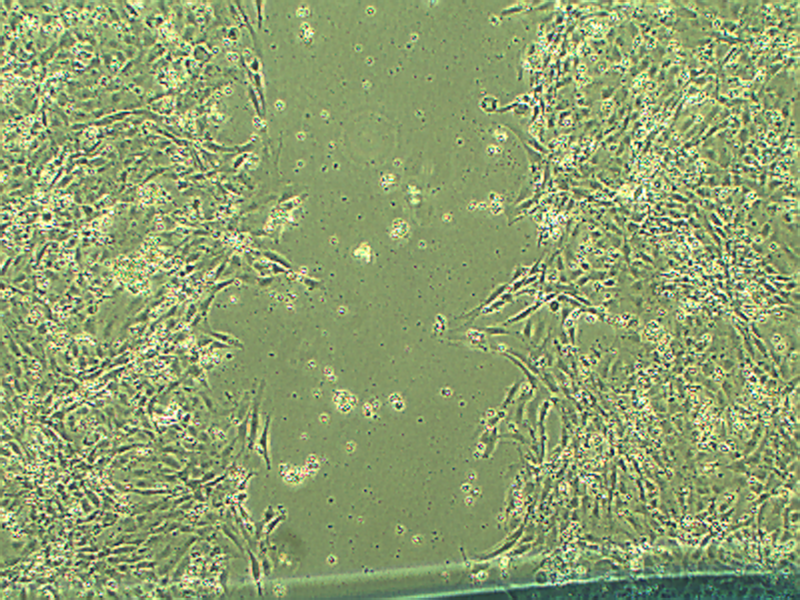

Supplement: Supplementary file 14 — Source Data for Figure 7 [file EMBR-24-e56870-s001.zip › Figure 7/7B/Kif3a KO 30 h 24 h.tif]

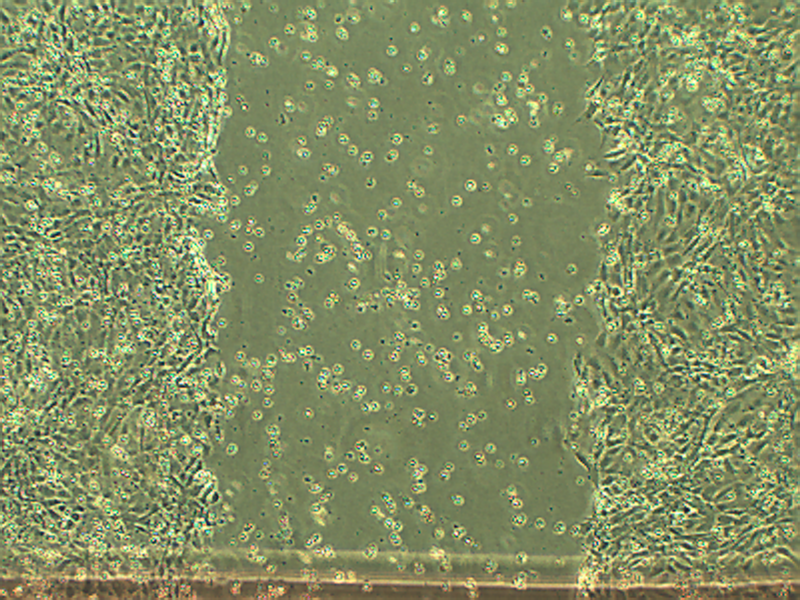

Supplement: Supplementary file 14 — Source Data for Figure 7 [file EMBR-24-e56870-s001.zip › Figure 7/7B/Kif3a KO 42 h 0 h.tif]

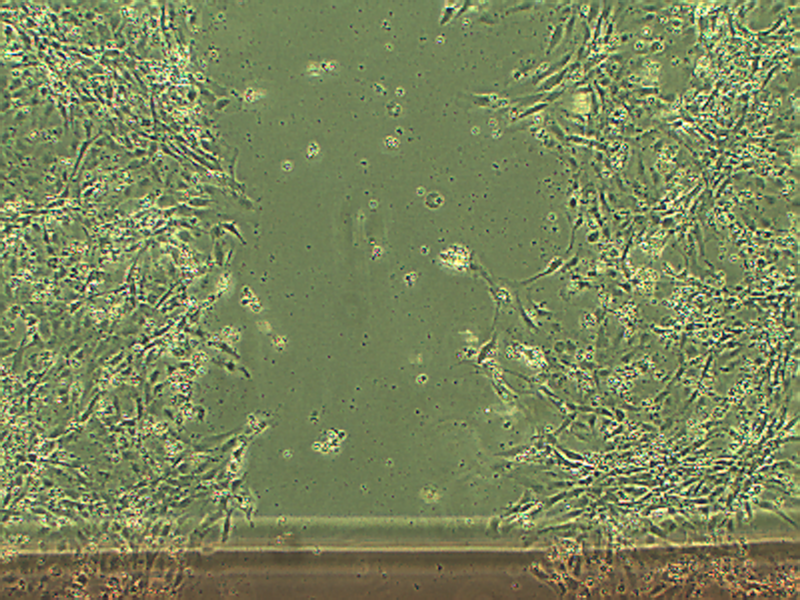

Supplement: Supplementary file 14 — Source Data for Figure 7 [file EMBR-24-e56870-s001.zip › Figure 7/7B/Kif3a KO 42 h 24 h.tif]

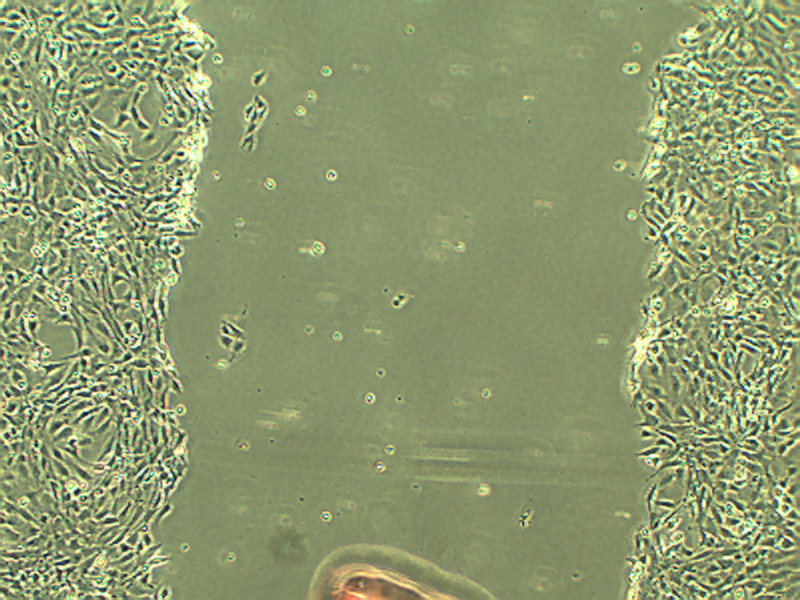

Supplement: Supplementary file 14 — Source Data for Figure 7 [file EMBR-24-e56870-s001.zip › Figure 7/7B/WT 18 h 0 h.tif]

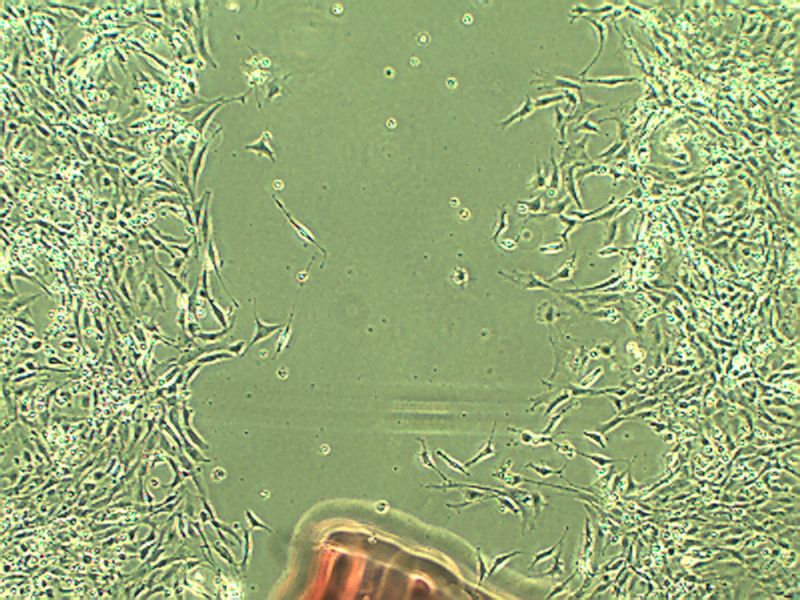

Supplement: Supplementary file 14 — Source Data for Figure 7 [file EMBR-24-e56870-s001.zip › Figure 7/7B/WT 18 h 24 h.tif]

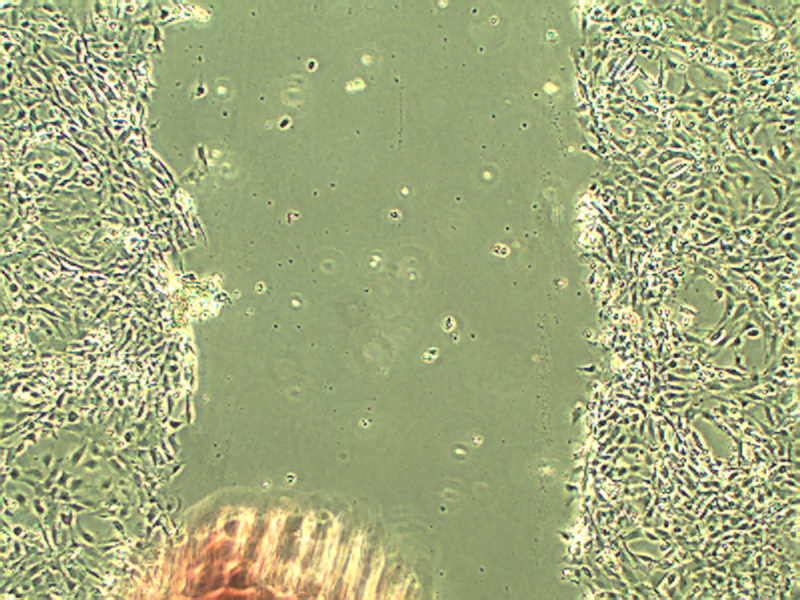

Supplement: Supplementary file 14 — Source Data for Figure 7 [file EMBR-24-e56870-s001.zip › Figure 7/7B/WT 30 h 0 h.tif]

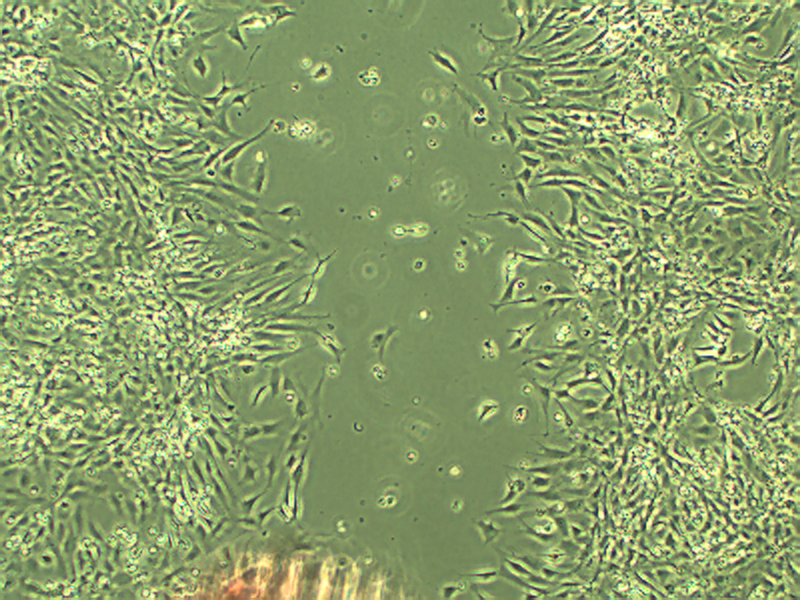

Supplement: Supplementary file 14 — Source Data for Figure 7 [file EMBR-24-e56870-s001.zip › Figure 7/7B/WT 30 h 24 h.tif]

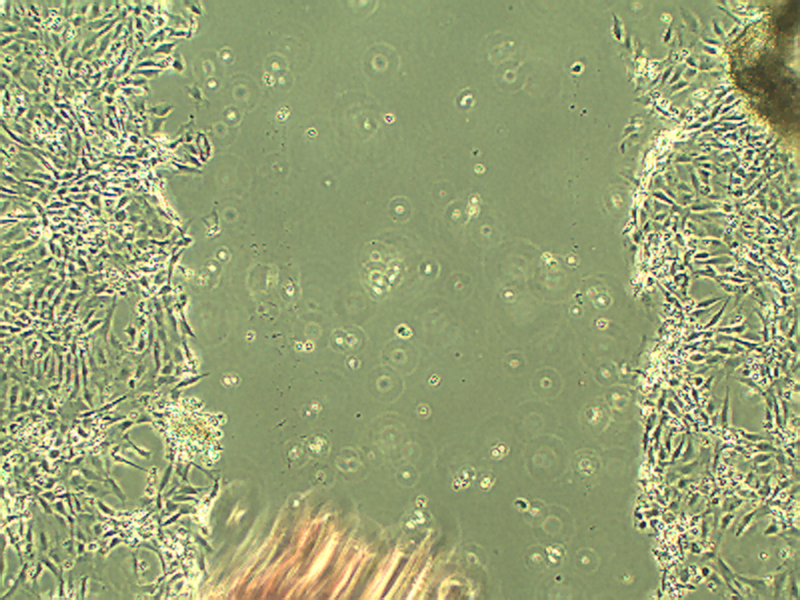

Supplement: Supplementary file 14 — Source Data for Figure 7 [file EMBR-24-e56870-s001.zip › Figure 7/7B/WT 42 h 0 h.tif]

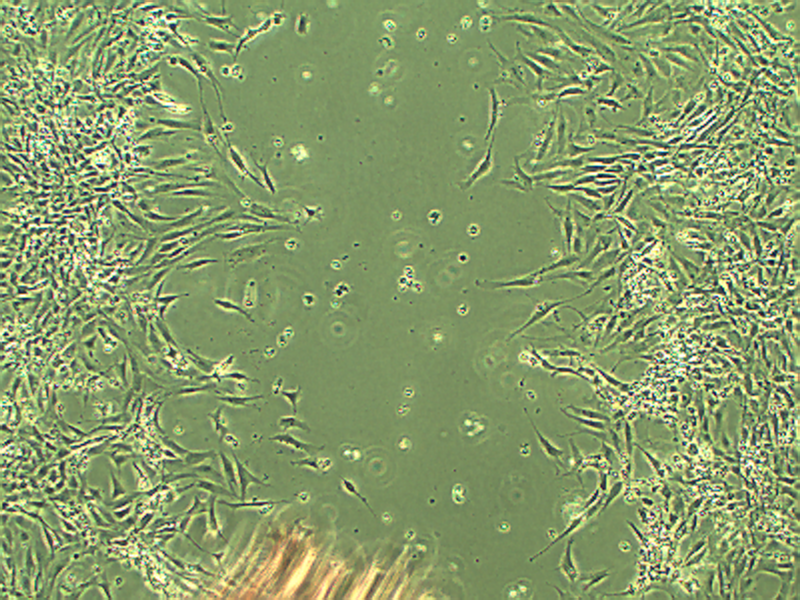

Supplement: Supplementary file 14 — Source Data for Figure 7 [file EMBR-24-e56870-s001.zip › Figure 7/7B/WT 42 h 24 h.tif]

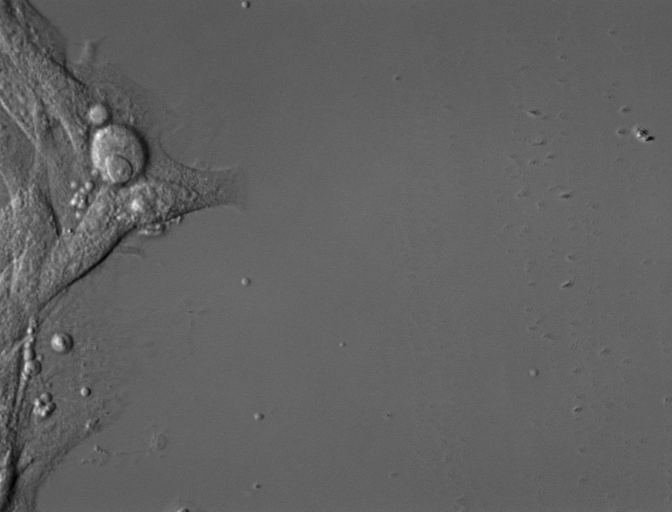

Supplement: Supplementary file 14 — Source Data for Figure 7 [file EMBR-24-e56870-s001.zip › Figure 7/7D/Wound at 18 h/0 h.tif]

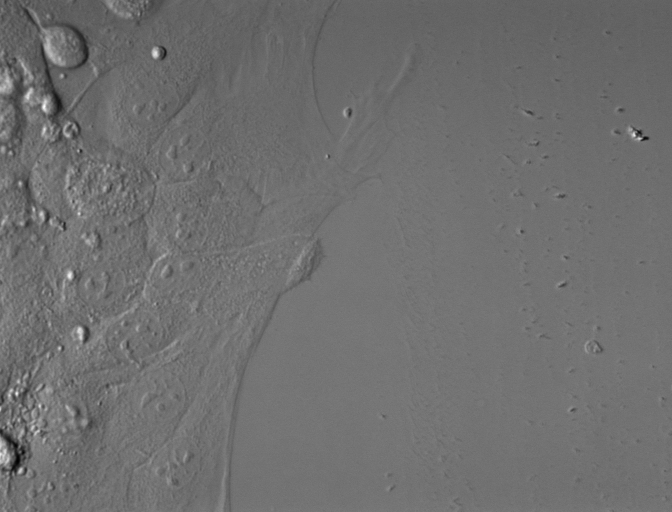

Supplement: Supplementary file 14 — Source Data for Figure 7 [file EMBR-24-e56870-s001.zip › Figure 7/7D/Wound at 18 h/12 h.tif]

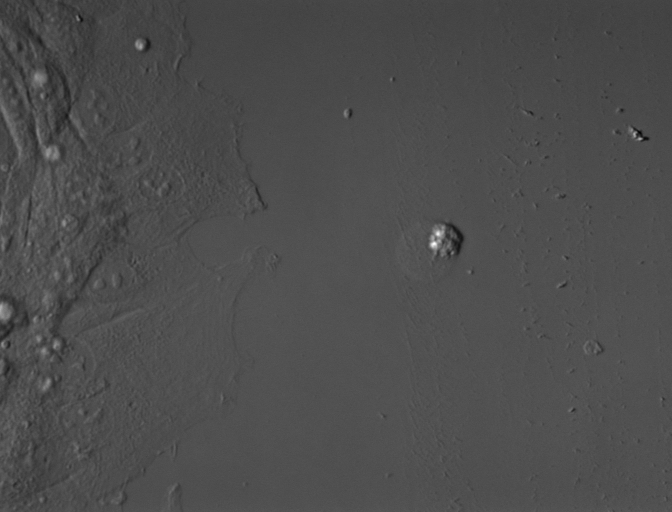

Supplement: Supplementary file 14 — Source Data for Figure 7 [file EMBR-24-e56870-s001.zip › Figure 7/7D/Wound at 18 h/4 h.tif]

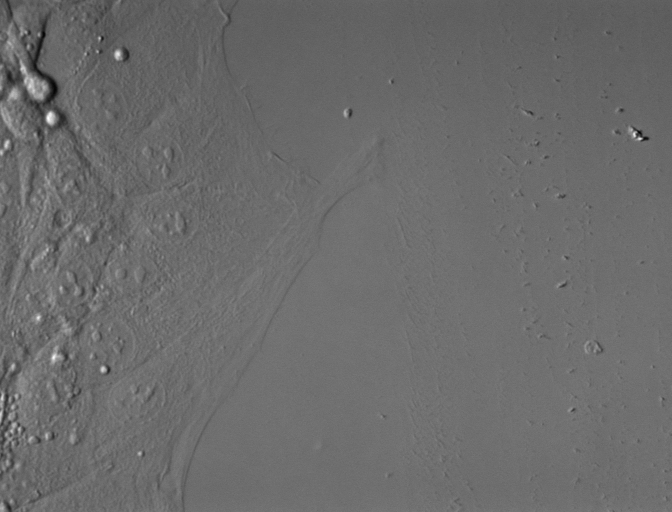

Supplement: Supplementary file 14 — Source Data for Figure 7 [file EMBR-24-e56870-s001.zip › Figure 7/7D/Wound at 18 h/8 h.tif]

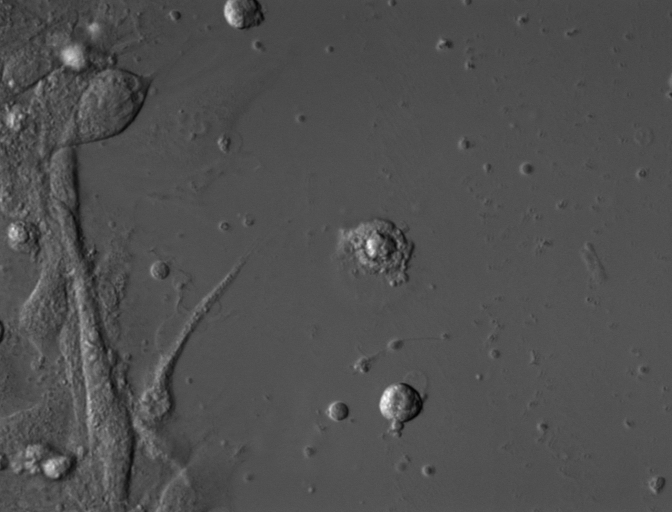

Supplement: Supplementary file 14 — Source Data for Figure 7 [file EMBR-24-e56870-s001.zip › Figure 7/7D/Wound at 30 h/0 h.tif]

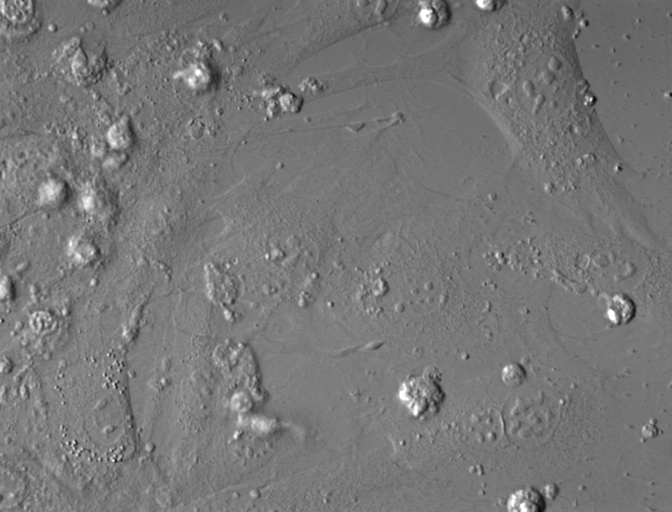

Supplement: Supplementary file 14 — Source Data for Figure 7 [file EMBR-24-e56870-s001.zip › Figure 7/7D/Wound at 30 h/12 h.tif]

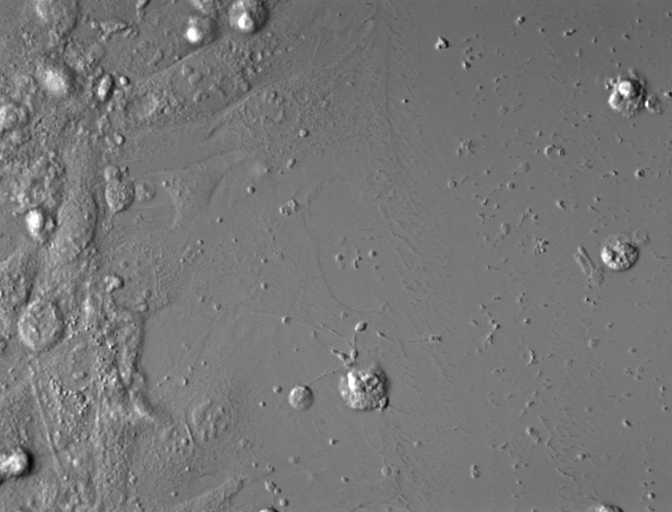

Supplement: Supplementary file 14 — Source Data for Figure 7 [file EMBR-24-e56870-s001.zip › Figure 7/7D/Wound at 30 h/4 h.tif]

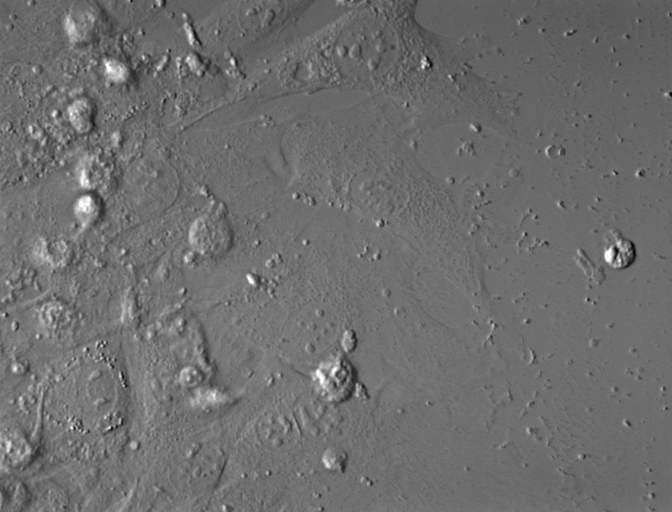

Supplement: Supplementary file 14 — Source Data for Figure 7 [file EMBR-24-e56870-s001.zip › Figure 7/7D/Wound at 30 h/8 h.tif]

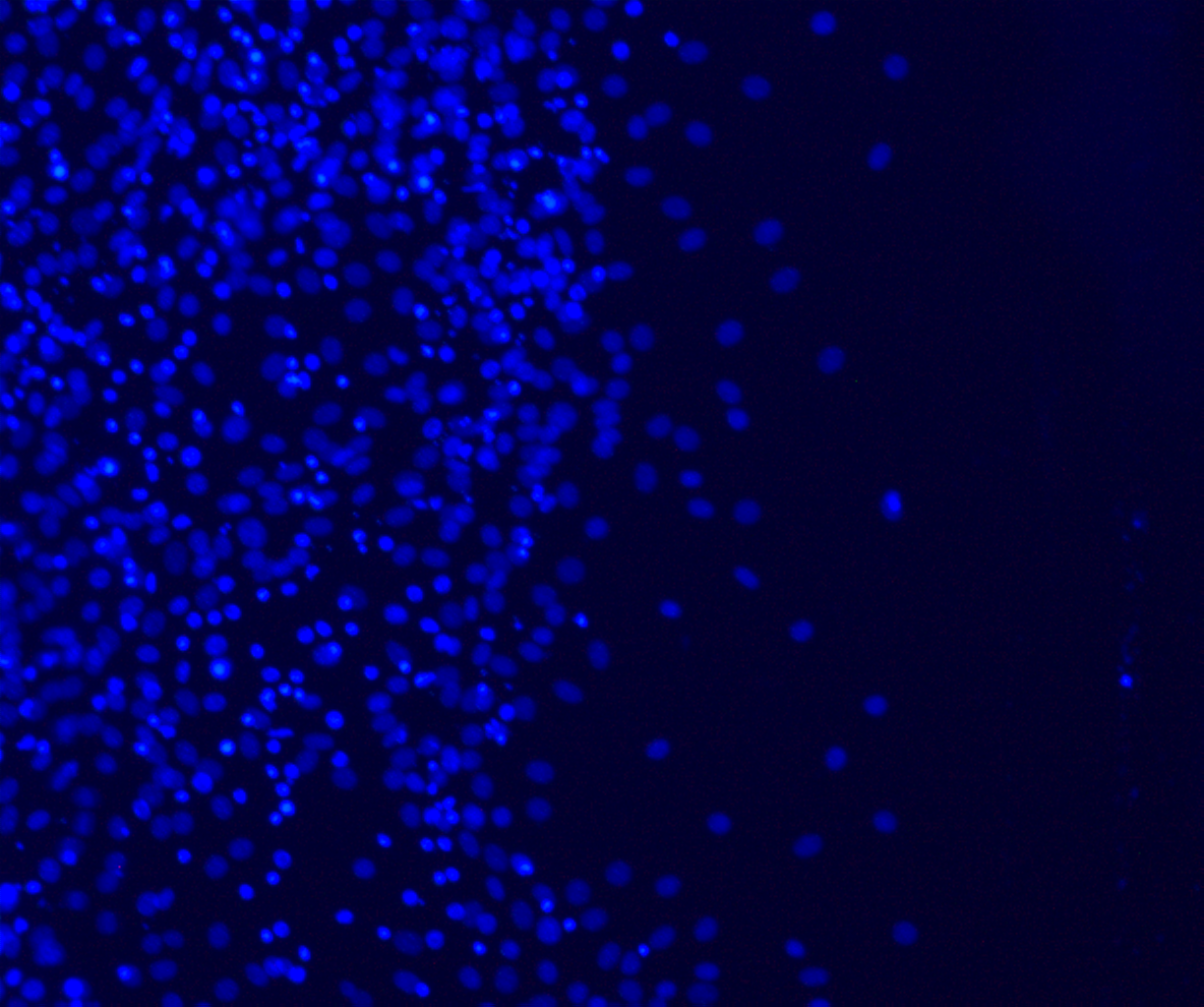

Supplement: Supplementary file 15 — Source Data for Figure 8 [file EMBR-24-e56870-s007.zip › Figure 8/8B/12 h after wound.tif]
