# Supplementary figures and images for: Circadian oscillation in primary cilium length by clock genes regulates fibroblast cell migration (part 2 of 2)
Source: EMBO Rep. 2023 Nov 16;24(12):e56870. doi: 10.15252/embr.202356870 (PMC10702818; doi:10.15252/embr.202356870)

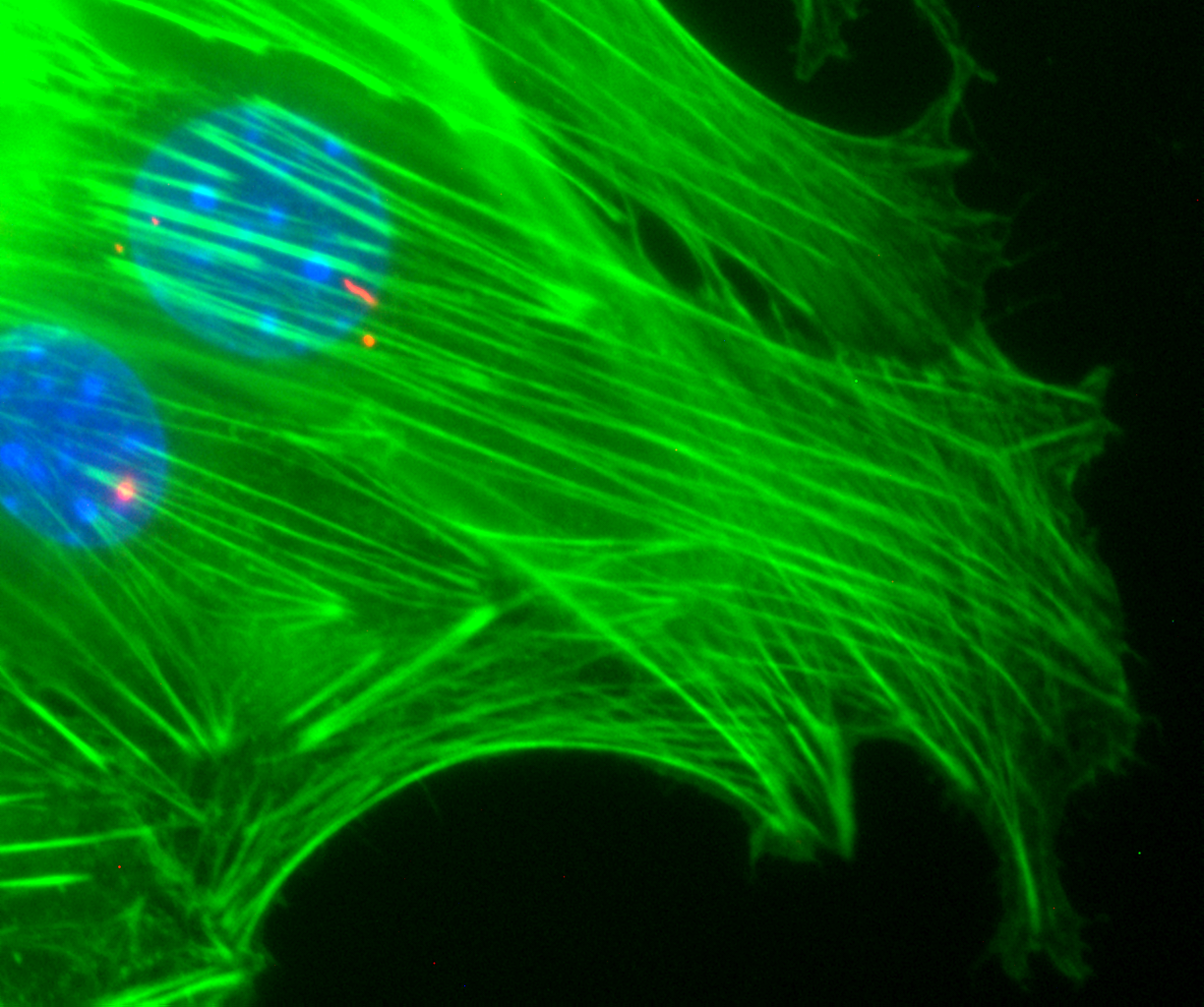

Supplement: Supplementary file 15 — Source Data for Figure 8 [file EMBR-24-e56870-s007.zip › Figure 8/8E/Wound at 30 h.tif]

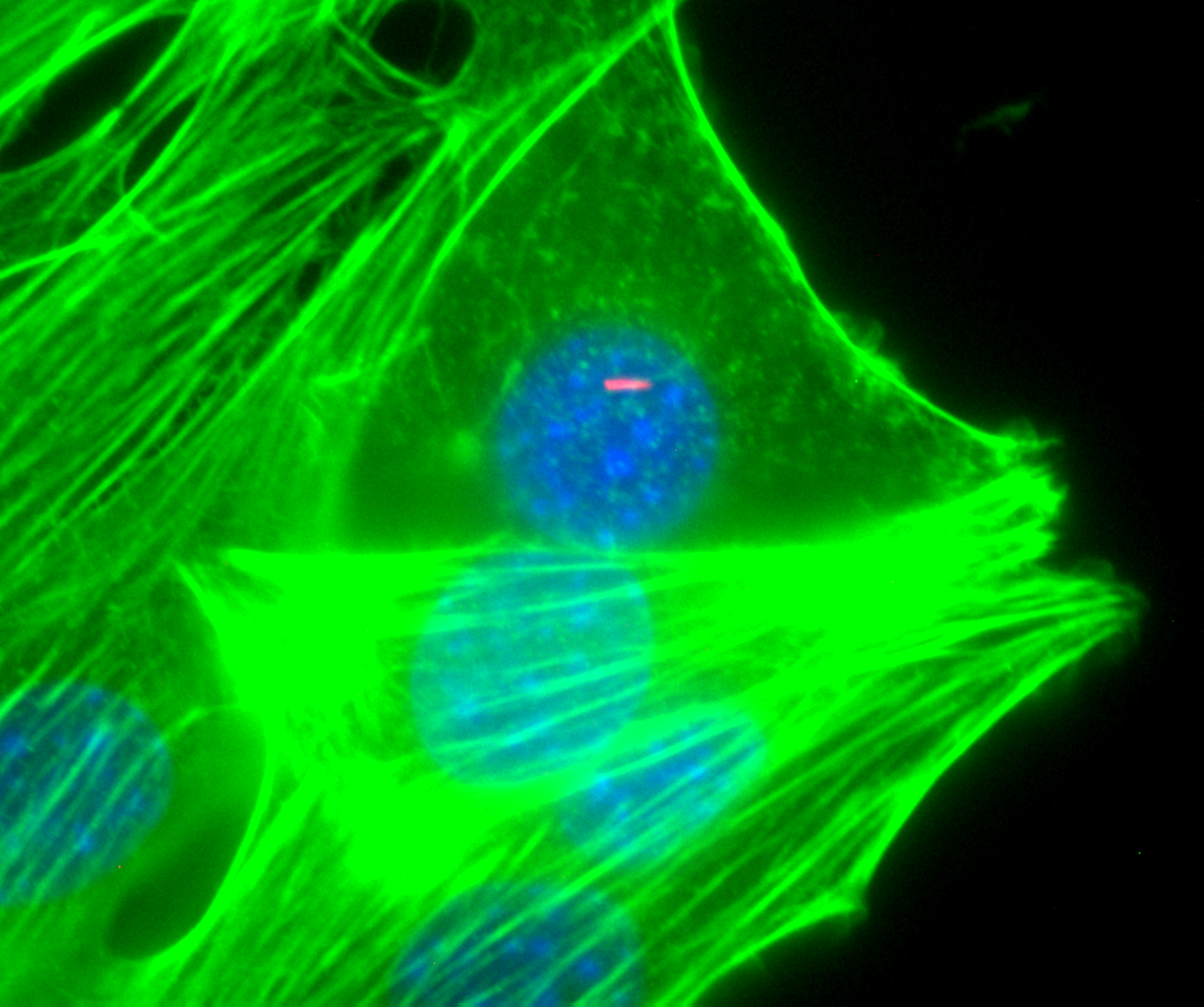

Supplement: Supplementary file 15 — Source Data for Figure 8 [file EMBR-24-e56870-s007.zip › Figure 8/8E/Wound at 18 h.tif]

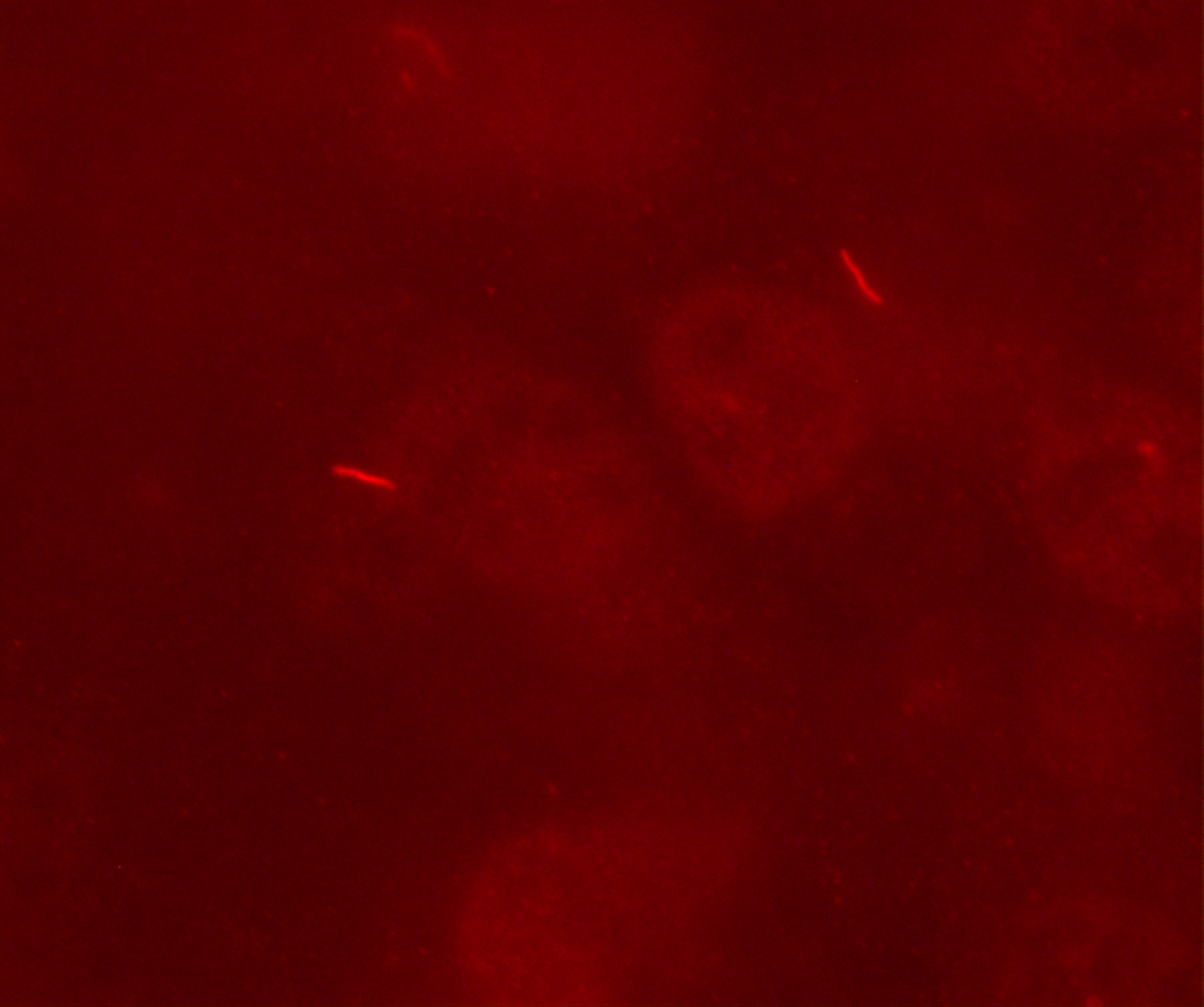

Supplement: Supplementary file 15 — Source Data for Figure 8 [file EMBR-24-e56870-s007.zip › Figure 8/8C/Wound at 18 h/II.tif]

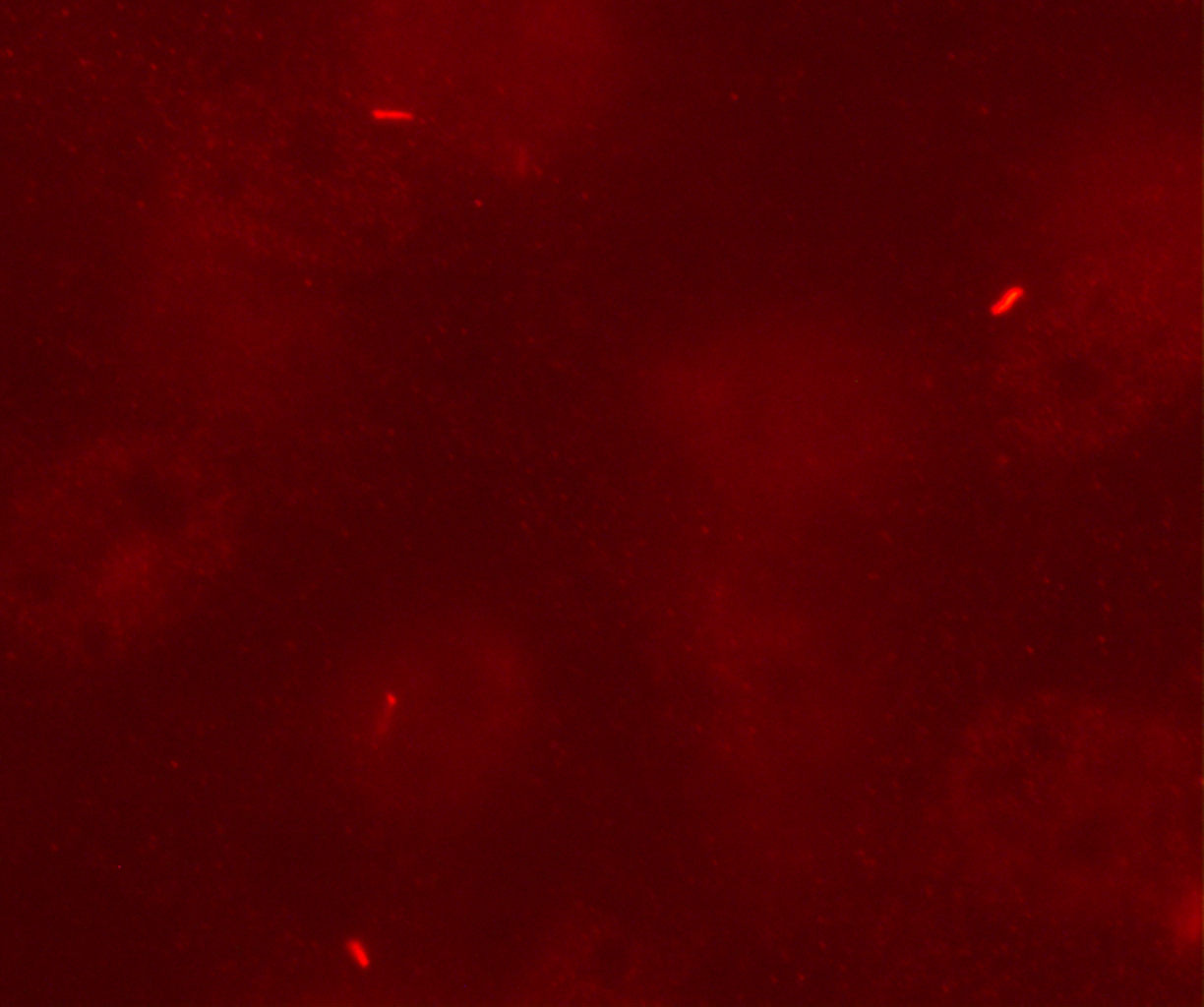

Supplement: Supplementary file 15 — Source Data for Figure 8 [file EMBR-24-e56870-s007.zip › Figure 8/8C/Wound at 18 h/I.tif]

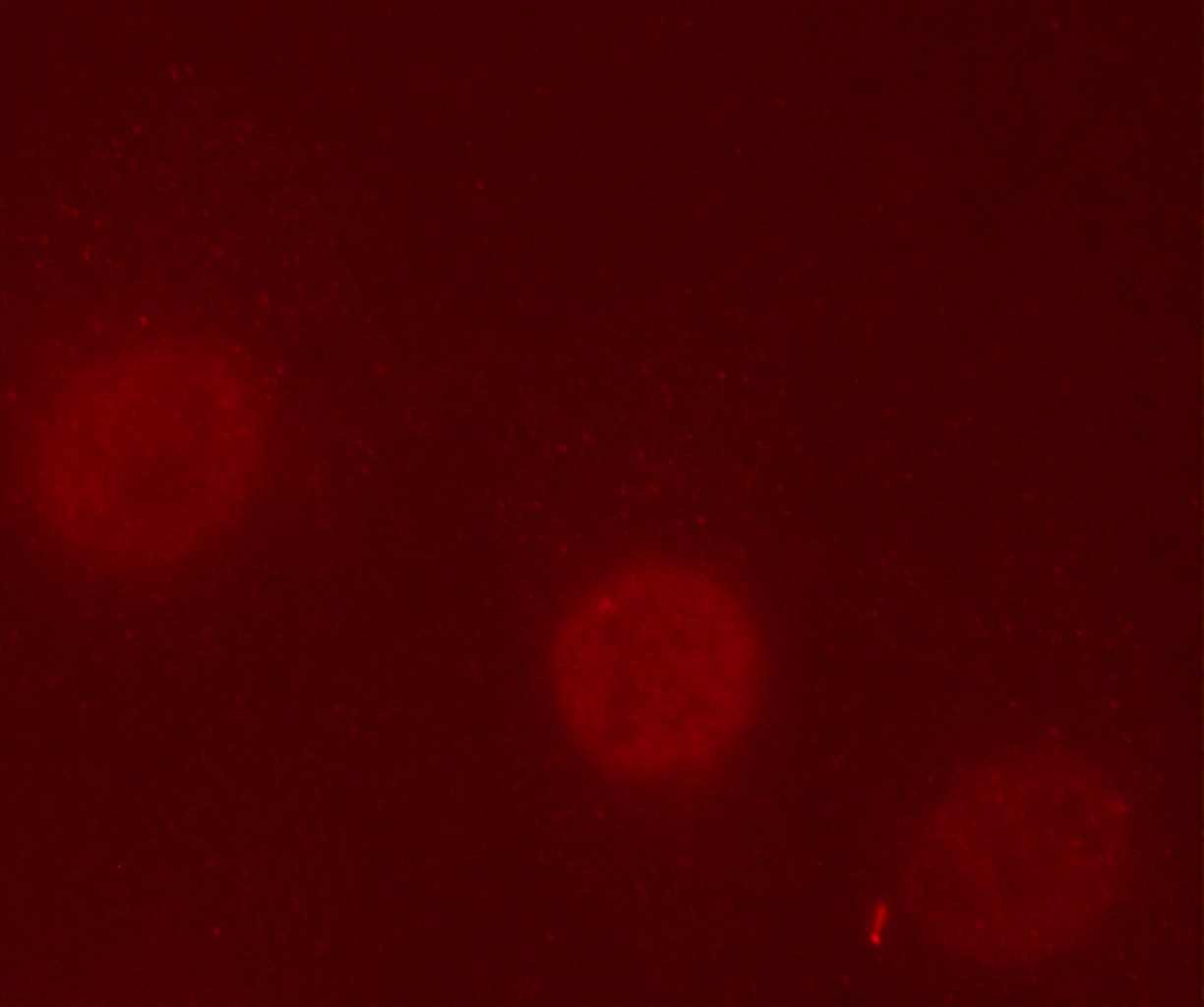

Supplement: Supplementary file 15 — Source Data for Figure 8 [file EMBR-24-e56870-s007.zip › Figure 8/8C/Wound at 18 h/III.tif]

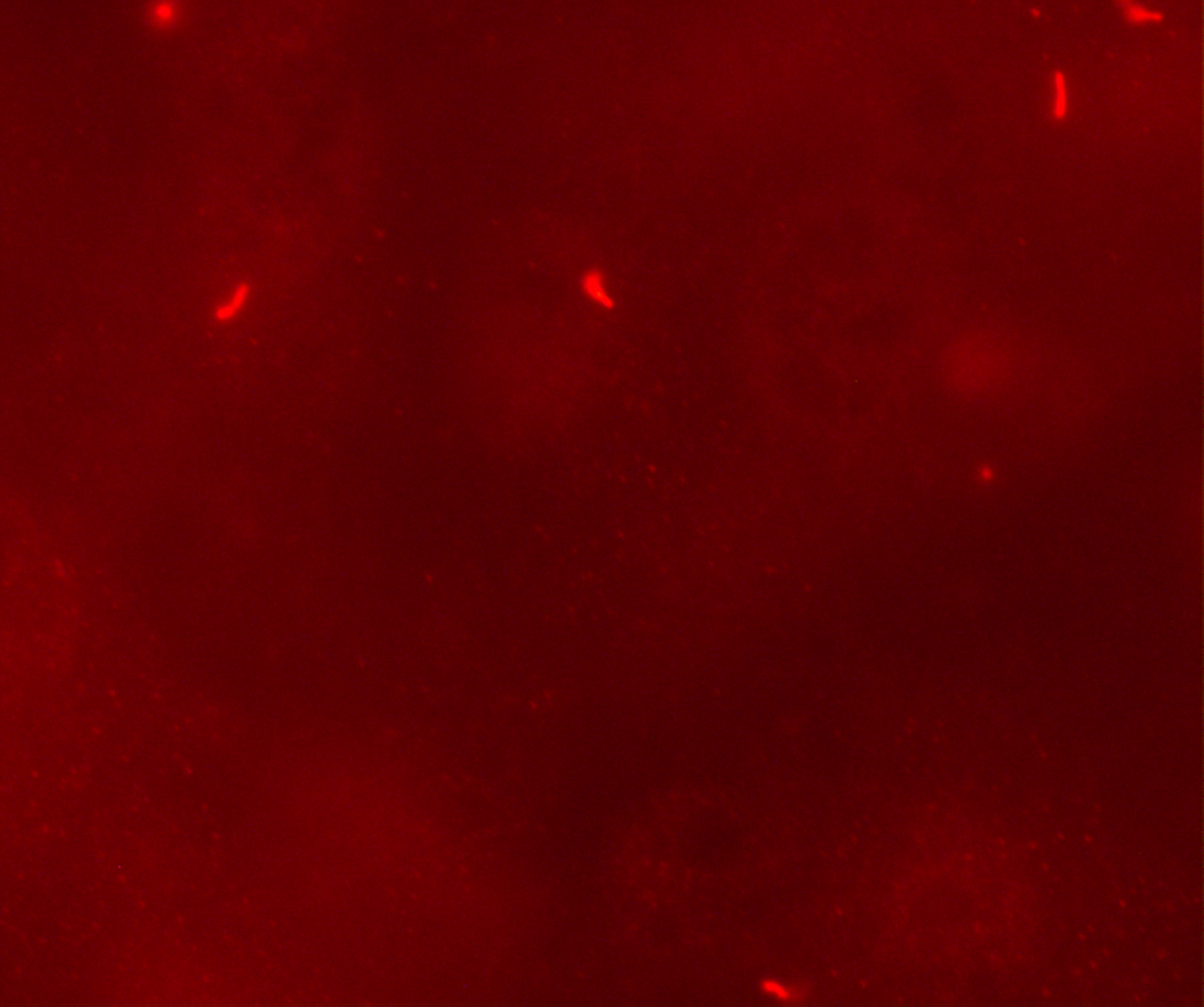

Supplement: Supplementary file 15 — Source Data for Figure 8 [file EMBR-24-e56870-s007.zip › Figure 8/8C/Wound at 30 h/II.tif]

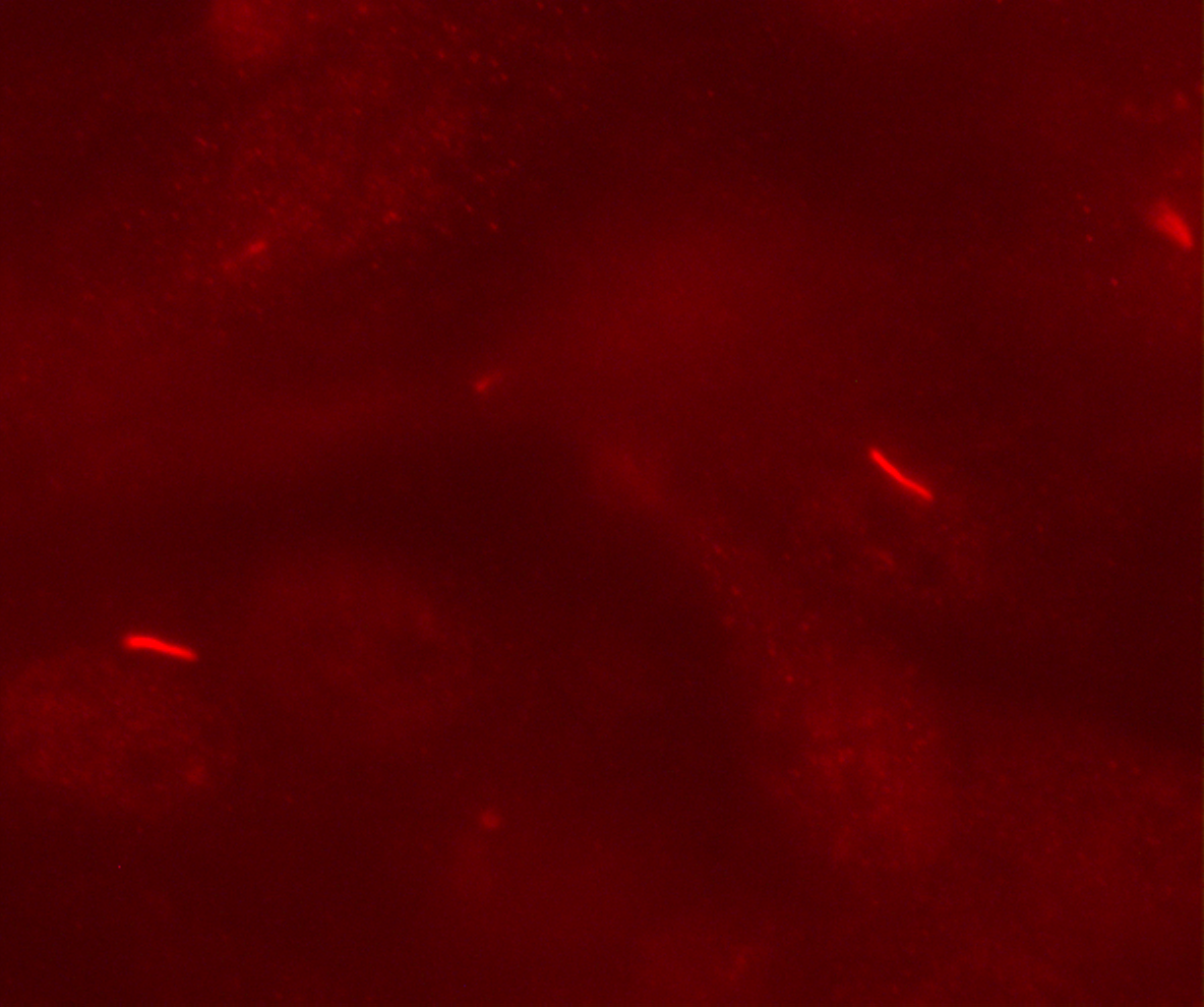

Supplement: Supplementary file 15 — Source Data for Figure 8 [file EMBR-24-e56870-s007.zip › Figure 8/8C/Wound at 30 h/I.tif]

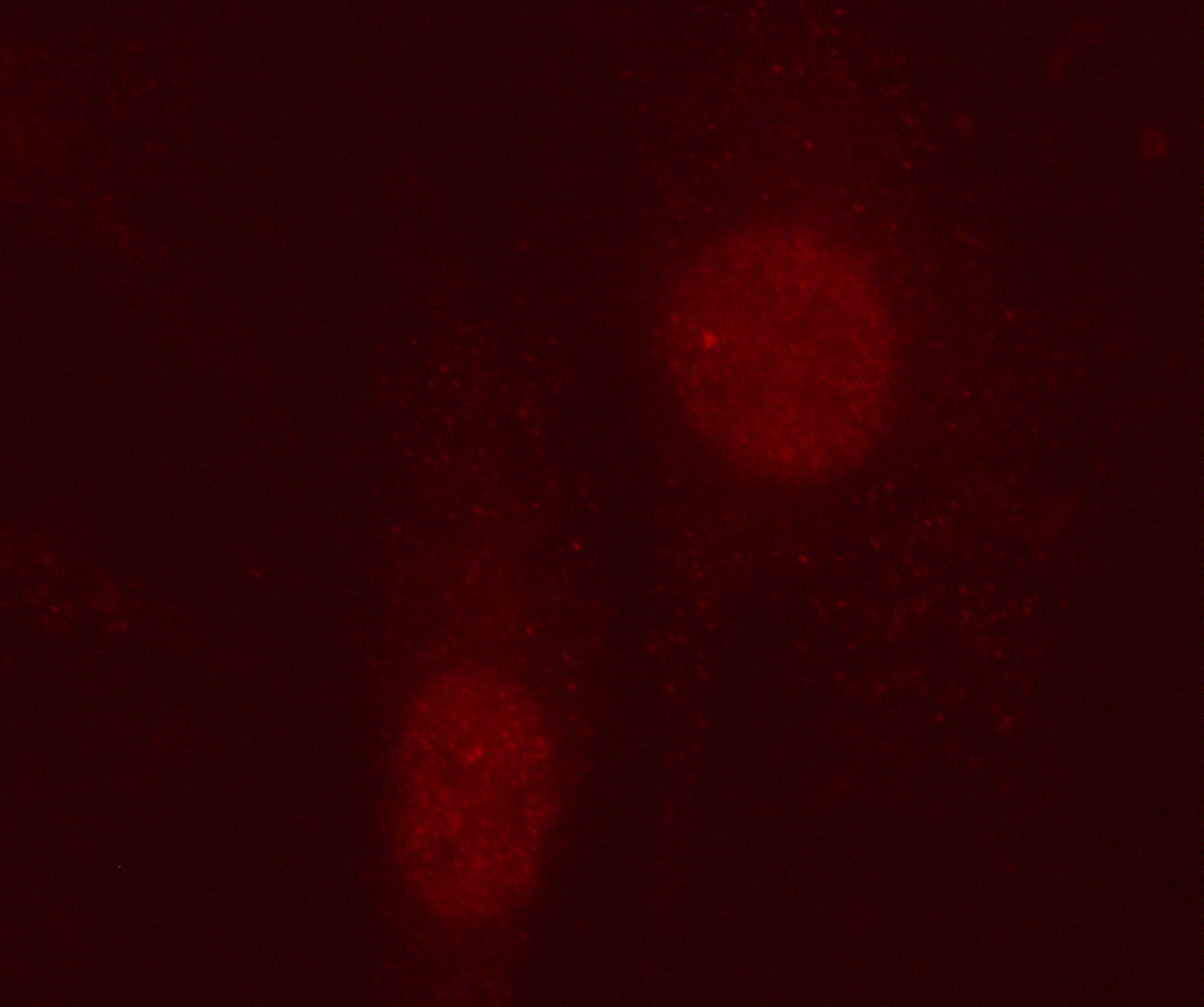

Supplement: Supplementary file 15 — Source Data for Figure 8 [file EMBR-24-e56870-s007.zip › Figure 8/8C/Wound at 30 h/III.tif]
