# Supplementary material for: Circadian oscillation in primary cilium length by clock genes regulates fibroblast cell migration
Source: EMBO Rep. 2023 Nov 16;24(12):e56870. doi: 10.15252/embr.202356870 (PMC10702818; doi:10.15252/embr.202356870)
Supplement: Supplementary file 16 [file EMBR-24-e56870-s015.pdf]

EMBO reports

Circadian oscillation in primary cilium length by clock genes regulate fibroblast cell migration

Ryota Nakazato, Yuki Matsuda, Faryal Ijaz, Koji Ikegami
DOI: 10.15252/embr.202356870

Corresponding author(s): Ryota Nakazato (nakazato@hiroshima-u.ac.jp), Koji Ikegami (k-ikegami@hiroshima-u.ac.jp)

Review Timeline:

Submission Date:	24th Jan 23
Editorial Decision:	8th Mar 23
Revision Received:	7th Jul 23
Editorial Decision:	18th Aug 23
Revision Received:	30th Aug 23
Accepted:	20th Oct 23

Editor: Ioannis Papaioannou / Deniz Senyilmaz Tiebe

Transaction Report:

(Note: With the exception of the correction of typographical or spelling errors that could be a source of ambiguity, letters and reports are not edited. Depending on transfer agreements, referee reports obtained elsewhere may or may not be included in this compilation. Referee reports are anonymous unless the Referee chooses to sign their reports.)

Dear Ryota,

Thank you for submitting your research manuscript for consideration by EMBO reports. It has been seen by three experts in the field, and we have received the full set of their reports (included below). I have already sent you a copy of the reports, and you have provided us with a point-by-point response to the comments as well as a proposed revision plan.

The referees acknowledge that the topic and the findings are interesting and novel, but they also identify several limitations and raise a number of concerns. According to your revision plan, you are willing and able to address essentially all points experimentally and/or with textual improvements.

Given these constructive comments, we would like to invite you to revise your manuscript along the lines you suggested in your revision plan with the understanding that the referee concerns (as detailed in their reports) must be fully addressed and their suggestions taken on board. Please address all referee concerns in a complete point-by-point response. Acceptance of the manuscript will depend on a positive outcome of a second round of review. It is EMBO reports policy to allow a single round of revision only and acceptance or rejection of the manuscript will therefore depend on the completeness of your responses included in the next, final version of the manuscript. If you have any questions or comments, we can also discuss the revisions in a video chat, if you like.

We realize that it is difficult to revise to a specific deadline. In the interest of protecting the conceptual advance provided by the work, we usually recommend a revision within 3 months (June 7th). Please discuss with me the revision progress ahead of this time if you require more time to complete the revisions.

*****
IMPORTANT NOTE:
We perform an initial quality control of all revised manuscripts before re-review. Your manuscript will FAIL this control and the handling will be DELAYED if the following APPLIES:

1) If a data availability section providing access to data deposited in public databases is missing. If you have not deposited any data, please add a sentence to the data availability section that explains that (see below for more information).

2) If your manuscript contains statistics and error bars based on n=2. Please use scatter plots in these cases. No statistics should be calculated if n=2.

When submitting your revised manuscript, please carefully review the instructions that follow below. Failure to include requested items will delay the evaluation of your revision.
*****

When submitting your revised manuscript, we will require:

1) A .docx formatted version of the manuscript text (including legends for main figures, EV figures and tables). Please make sure that the changes are highlighted to be clearly visible.

2) Individual production quality figure files as .eps, .tif, .jpg (one file per figure).
Please download our Figure Preparation Guidelines (figure preparation pdf) from our Author Guidelines pages https://www.embopress.org/page/journal/14693178/authorguide for more info on how to prepare your figures.

3) A .docx formatted letter INCLUDING the reviewers' reports and your detailed point-by-point responses to their comments. As part of the EMBO Press transparent editorial process, the point-by-point response is part of the Review Process File (RPF), which will be published alongside your paper unless you opt out of this (please see below for further information).

4) A complete author checklist, which you can download from our author guidelines (<https://www.embopress.org/page/journal/14693178/authorguide>). Please insert information in the checklist that is also reflected in the manuscript. The completed author checklist will also be part of the RPF (please see below for more information).

5) Please note that all corresponding authors are required to supply an ORCID ID for their name upon submission of a revised manuscript (<https://orcid.org/>). Please find instructions on how to link your ORCID ID to your account in our manuscript tracking system in our Author guidelines (<https://www.embopress.org/page/journal/14693178/authorguide#authorshipguidelines>)

6) We replaced Supplementary Information with Expanded View (EV) Figures and Tables that are collapsible/expandable online.

A maximum of 5 EV Figures can be typeset. EV Figures should be cited as 'Figure EV1, Figure EV2" etc... in the text and their respective legends should be included in the main text after the legends of regular figures.

- For the figures that you do NOT wish to display as Expanded View figures, they should be bundled together with their legends in a single PDF file called *Appendix*, which should start with a short Table of Content. Appendix figures should be referred to in the main text as: "Appendix Figure S1, Appendix Figure S2" etc. See detailed instructions regarding expanded view here: <https://www.embopress.org/page/journal/14693178/authorguide#expandedview>

- Additional Tables/Datasets should be labeled and referred to as Table EV1, Dataset EV1, etc. Legends have to be provided in a separate tab in case of .xls files. Alternatively, the legend can be supplied as a separate text file (README) and zipped together with the Table/Dataset file.

7) Please note that a "Data availability" section at the end of Materials and Methods is now mandatory. In case you have no data that require deposition in a public database, please state so instead of refereeing to the database: "Our study includes no data deposited in public repositories." under the heading "Data availability".
See also <https://www.embopress.org/page/journal/14693178/authorguide#dataavailability>). Please note that the Data availability statement is restricted to new primary data that are part of this study.

8) We request authors to consider both actual and perceived competing interests. Please review the new policy (<https://www.embopress.org/competing-interests>) and update your competing interests statement if necessary. Please name this section 'Disclosure and competing interests statement' and place it after the Acknowledgements section.

9) Figure legends and data quantification:
The following points must be specified in each figure legend:

- the name of the statistical test used to generate error bars and P values,
- the number (n) of independent experiments (please specify technical or biological replicates) underlying each data point,
- the nature of the bars and error bars (s.d., s.e.m.)
- If the data are obtained from n {less than or equal to} 2, use scatter plots showing the individual data points.

Discussion of statistical methodology can be reported in the materials and methods section, but figure legends should contain a basic description of n, P and the test applied.

See also the guidelines for figure legend preparation:
https://www.embopress.org/page/journal/14693178/authorguide#figureformat

- Please also include scale bars in all microscopy images.

10) We now request publication of original source data with the aim of making primary data more accessible and transparent to the reader. Our source data coordinator will contact you to discuss which figure panels we would need source data for and will also provide you with helpful tips on how to upload and organize the files.

11) Our journal encourages inclusion of *data citations in the reference list* to directly cite datasets that were re-used and obtained from public databases. Data citations in the article text are distinct from normal bibliographical citations and should directly link to the database records from which the data can be accessed. In the main text, data citations are formatted as follows: "Data ref: Smith et al, 2001" or "Data ref: NCBI Sequence Read Archive PRJNA342805, 2017". In the Reference list, data citations must be labeled with "[DATASET]". A data reference must provide the database name, accession number/identifiers and a resolvable link to the landing page from which the data can be accessed at the end of the reference. Further instructions are available at <https://www.embopress.org/page/journal/14693178/authorguide#referencesformat>.

12) Please also note our reference format:
<http://www.embopress.org/page/journal/14693178/authorguide#referencesformat>.

13) We now use CRediT to specify the contributions of each author in the journal submission system. CRediT replaces the author contribution section, which should be removed from the manuscript. Please use the free text box to provide more detailed descriptions. See also guide to authors:
<https://www.embopress.org/page/journal/14693178/authorguide#authorshipguidelines>.

14) As part of the EMBO publications' Transparent Editorial Process, EMBO reports publishes online a Review Process File to accompany accepted manuscripts. This File will be published in conjunction with your paper and will include the referee reports, your point-by-point response and all pertinent correspondence relating to the manuscript.

You can opt out of this by letting the editorial office know (emboreports@embo.org). If you do opt out, the Review Process File link will point to the following statement: "No Review Process File is available with this article, as the authors have chosen not to

make the review process public in this case."

We would also welcome the submission of cover suggestions, or motifs to be used by our Graphics Illustrator in designing a cover.

I look forward to seeing a revised version of your manuscript when it is ready. Please let me know if you have any questions or comments regarding the revision.

Best regards,

Ioannis

Ioannis Papaioannou, PhD
Editor
EMBO reports

Referee #1:

This work by Nakazato et al reports a number of interesting observations, including circadian variation of primary cilia length in several cell types in vivo, primary cilia length in NIH3T3 cells in vitro, and wound-induced fibroblast migration in vitro. The authors combine strong immunocytochemical analyses for various markers of primary cilia and their assembly with perturbations including a REVERBa agonist and wounding assays. The experiments are well described and executed. The authors are applauded for efforts to replicate their findings (e.g. Fig. 1E and 2G) in vitro and compare to in vivo measures (Fig. 3C). Based on their data, the authors conclude that "circadian dynamics of primary cilia length by clock genes control fibroblast migration" (abstract) "by regulating PCNT accumulation in centriolar satellites" (discussion). This conclusion is not fully supported by the findings. The manuscript will be greatly improved when the authors address the following major and minor concerns.

Major critiques:

1. The major conclusion is that primary cilia length is under control of the molecular circadian clock. The evidence for cell-intrinsic circadian regulation of primary cell length is based on data that are also consistent with other models such as DEX acutely decrease cilia length without circadian regulation. The best evidence for circadian regulation comes from cultures collected at 4-hour intervals over 24 h (Figures 1D, 1E and 2G) in response to DEX. Unfortunately, this does not meet the standards for demonstrating circadian regulation (Hughes et al., 2017, PMID 29098954). Collecting samples for an additional 24 h would reveal the potential for circadian increases and decreases in cilia length.
2. The authors conclude that there is circadian regulation of primary cilia in NIH3T3 cells and hippocampal neurons, but not astrocytes. This is hard to reconcile. For example, it is only possible explain their observation that 40% of desynchronized NIH3T3 cells have cilia at all times and 30-60% of synchronized cells have cilia if we assume only 20-60% of NIH3T3 cells have detectable circadian regulation of primary cilia. The authors should state this. In addition, the authors should consider live-cell imaging of cilia length for at least 48 h so that we can see the dynamics they infer from fixed and pooled samples.
3. Conflating cilia length with ciliation status - changes in the two are functionally different. This mistake is exemplified in the Kif3a KO experiments where they model disrupted circadian rhythm in cilia length using a total loss of cilia, which produces many consequences other than an isolated effect on circadian cilia length.
4. Conflating correlation with causation - comparing wound healing differences in cells at circadian time 30 h ("short cilia stage") vs. 18 or 42 h ("long cilia stage") does not prove causality between cilia length and wound healing. It's akin to saying if I'm asleep in the night and the sky also happens to be dark at night, then my sleep-wake cycle is controlling the sky. If there is to be a mechanistic link, then how might the authors propose that cilia are actually regulating cell migration? This key question remains unanswered. Signaling pathways were alluded - including cilia-mediated cell sensitivity to mechanical stimuli, which is plausible - but no experimental test for these hypotheses.
5. The authors use a non-specific agonist of Reverba (SR9011) to test for a causal link from the circadian clock to cilia length. This Rev-erb agonist suppresses the core clock as well as affects many REVERBa-target genes. It is unclear if the drug directly affects circadian clock genes, cell migration, or primary cilia growth. The authors rely on 2 vs 10 uM SR9011 to segregate cilia elongation and inhibit circadian dynamics of cilia without careful controls to show dose dependence.

Minor points:
6. Quantitation of cilia number and length: analysis method not discussed. Are these manually counted and measured, or did the authors use established automated cilia quantification ImageJ plugins e.g. CiliaQ? What is the number of cells and cilia

quantitated, how many hpf?
7. Pericentrin localizing to satellites is not itself mechanistic support for circadian control of ciliation. What is the actual mechanism? Does PCNT have Reverb/Bmal binding sites in its promoter? Is this protein produced, transported, degraded in a circadian manner?
8. Do cilia length fluctuations actually represent the ciliary cycle of reabsorption and growth? Can they estimate the minimum and maximum growth and resorption rates that would be required to explain their changes in cilia length over the day? Are these rates consistent with known rates of assembly and disassembly?
9. The authors state that this is independent of the cell cycle. What is the evidence for a cilia circadian cycle independent of the cell cycle, and what is its purpose?
10. The authors mention without citation that circadian rhythms in primary cilia length has been reported previously. I found several on Bioarxiv. It would be good to put this study into context with appropriate citations of peer-reviewed papers on circadian primary cilia.

Referee #2:

In this manuscript, Nakazato et. al. elucidated the regulation of ciliogenesis and cilium length by circadian rhythm. They showed that number and length of primary cilia in murine fibroblast cells show similar dynamics with rhythmic oscillations of clock genes. They also demonstrate similar oscillation in primary cilia of neurons, and astrocytes of the murine brain in vivo. Moreover, they investigated the mechanism by which the clock regulates primary cilium length by focusing on centriolar satellite protein Pericentrin and showed that its distribution in cells also changes during rhythmic clock oscillations. Finally, they used wound healing assays to show that cilium length changes contribute to the migration defects.

This manuscript is of general interest due to its focus on dissecting new relationship between circadian rhythm and primary cilia regulation, two important biological processes deregulated in disease. The relationship between circadian rhythm and primary cilium regulation has not been studies before, which is the novel and exciting part of the manuscript. The findings from the first two figures are therefore very important and opens new areas of investigation. However, the characterization of the mechanisms that underlie ciliary length changes and their functional relevance is premature and the data presented for these parts are not solid and quantatitative (i.e. centriolar satelite phenotypes). Moreover, the conclusions reached for certain phenotypes were based on experiments that disrupt many cellular events (i.e. microtubule depolymerization, SR9011 treatment). These experiments suffer from specificity. Finally, the manuscript in most parts, particularly in introduction, does not have the proper references for the concepts introduced and discussed. Manuscript text has to be revised carefully to improve clarity in introduction, results and conclusions. Below are some of my major and minor comments:

1- Although the authors only quantified cilium formation efficiency and length in functional assays, they refer to their analysis as investigation of "cilium morphology", which is not right. Morphology of cilia can only be assessed by imaging approaches with higher resolution (i.e. SEM, expansion microscopy).
2- Fig.1: The quantification of the clock oscillations after DEX induction was performed over 96 h. To determine whether the ciliary oscillations of biogenesis and length correlates with clock oscillation, % of ciliated cells and cilium length can be quantified over 96 h instead of 48 h.
3- Fig. 4A-C: The major mechanism the authors focused on for explaining the ciliary changes during circadian oscillations are centriolar satellite distribution and number. However, they only analyzed this angle by focusing on pericentrin, a protein that localizes both to the centrosome and cilia (Fig. 4). To convincingly show that centriolar satellites in part underlie the ciliary changes after DEX induction, centriolar satellites should be marked by their scaffolding protein PCM1 and their number, fluorescent intensity and cellular distribution should be quantified with better quantitative approaches than the ones used for Pericentrin (i.e. Galati, DF et al. Dev Cell 2018). Similar quantitative analysis should also be performed for pericentrin, with an adaptation to remove the centrosomal signal of pericentirn.
4- Fig. 4D-J: Colchicine treatment not only disrupts centriolar satellite positioning, but also affects other cellular structures transported by microtubules and function during cilium biogenesis. Therefore, this experiment is not sufficient to conclude that satellite positioning and thereby transport underlie the ciliary changes upon DEX induction. The method used by Aydin et al. 2021 to misposition satellites to the cell periphery can be used to specifically test the role of satellites in ciliary regulation during circadian oscillations.
5- To uncover the mechanism by which cilium length is regulated after DEX induction, other key regulators of cilium length such as basal body/cilium recruitment of IFT proteins should be assessed after DEX induction. Microscopy-based analysis of centrosomal or ciliary levels of ciliogenesis factors at different time points after DEX induction will strengthen the conclusions of the manuscript on the link between clock and cilium length regulation.
6- In addition to cell migration, ciliary functions such as Hedgehog signaling can also be checked to determine the physiological relevance of length-clock relationship and will complement the manuscript better.
7- Fig. 6: What were the parameters used for defining cells facing the wound and cells invading the wound area?

Minor:
References and text should be revised carefully to include the important references for the concepts included in the manuscript

and to improve clarity. I listed below some of examples to this comment:
1- Reference is missing for the following sentence in introduction:
"For example, the autonomous cellular clock regulates fibroblast
migration by controlling actin dynamics."
2- Revise the following sentence in introduction: "Defects in primary cilia due to congenital factors..."
3- "static" should be removed or replaced by "motile" in the following sentence: "Primary cilia are immotile, but not static."
4- Detailed introduction to ciliary decapitation and signaling can be removed from the introduction as the major focus of the manuscript is cilium assembly and length control.
5- The following sentence is not clear:
"Numerous studies on the effects of loss or mutation of primary cilia on cellular
function in ciliopathy have revealed much regarding the importance of the "presence" of primary cilia."
6- The paragraph about cilium length and morphology control should be revised to introduce the state-of-art. There are several comprehensive reviews on cilium length control and its physiological relevance (i.e. Broekhuis, JR. 2013, Keeling, J. 2016). These should be cited and included in the introduction.
7- There are several important papers that uncovered the relationship between pericentrin, centriolar satellites and primary cilium (i.e. Chad Pearson's lab). They should be cited in the results part for Fig. 4 or even better in the introduction.

Referee #3:

Review of "Circadian oscillation in primary cilium length by clock genes regulate fibroblast cell migration"
- Ryota Nakazato, Yuki Matsuda, Koji Ikegami

The authors of this manuscript provide evidence that a cell's cilia length is under control of the circadian clockwork, and that this control influences cell motility using a combination of cell-culture and in vivo approaches. This is an interesting and novel area - circadian control of cilia/cell motility in mammalian cells. The authors data generally support their overall conclusions, but could use several modifications, clarifications and analyses, as detailed below.

1. To say this is regulated by clock "genes" is substantially overstated. The authors did not directly disrupt clock genes, the only clock perturbation used is drug that may have unknown effects outside of modulating RevErb activity. However, they can simply delete "by clock genes" from the title and throughout without diminishing anything.
2. Cilia length - how did they account for cilia projecting in different directions in 3D space? Is it possible that cilia lengths are sometimes in the z-axis? This would make them 'appear' shorter in a 2D confocal image (i.e. Fig 3D for example). This is an important potential caveat the authors should at least discuss. The differences observed in "length" could potentially be due to synchronized location/orientation instead of actual size.
3. Statistics throughout the manuscript are inadequate. The authors use T-tests in place of ANOVAs (and Two-Way ANOVAs) throughout - this must be fixed for the sake of demonstrating experimental rigor. Similarly, the "n" used is often confusing - they state "3 experiments" but the measure is "number of cells" - n should be "number of cells".
4. Several of their images are impossible to see what the authors are describing. Fig 1C and 6C are particularly bad - I cannot see even blown on my screen the differences reported.

Referee #1:

This work by Nakazato et al reports a number of interesting observations, including circadian variation of primary cilia length in several cell types in vivo, primary cilia length in NIH3T3 cells in vitro, and wound-induced fibroblast migration in vitro. The authors combine strong immunocytochemical analyses for various markers of primary cilia and their assembly with perturbations including a REVERBa agonist and wounding assays. The experiments are well described and executed. The authors are applauded for efforts to replicate their findings (e.g. Fig. 1E and 2G) in vitro and compare to in vivo measures (Fig. 3C). Based on their data, the authors conclude that "circadian dynamics of primary cilia length by clock genes control fibroblast migration" (abstract) "by regulating PCNT accumulation in centriolar satellites" (discussion). This conclusion is not fully supported by the findings. The manuscript will be greatly improved when the authors address the following major and minor concerns.

> We appreciate the reviewer for the constructive and encouraging comments, by which our work have gotten improved greatly.

Major critiques:

1. The major conclusion is that primary cilia length is under control of the molecular circadian clock. The evidence for cell-intrinsic circadian regulation of primary cell length is based on data that are also consistent with other models such as DEX acutely decrease cilia length without circadian regulation. The best evidence for circadian regulation comes from cultures collected at 4-hour intervals over 24 h (Figures 1D, 1E and 2G) in response to DEX. Unfortunately, this does not meet the standards for demonstrating circadian regulation (Hughes et al., 2017, PMID 29098954). Collecting samples for an additional 24 h would reveal the potential for circadian increases and decreases in cilia length.

> We have collected samples at 4-hour intervals with an additional 24-hour culture. We replaced Fig 1E with the new one, and added data in Fig. EV1A and EV1C for Fig. 1D and 2G, respectively.

2. The authors conclude that there is circadian regulation of primary cilia in NIH3T3 cells and hippocampal neurons, but not astrocytes. This is hard to reconcile. For example, it is only possible explain their observation that 40% of desynchronized NIH3T3 cells have cilia at all times and 30-60% of synchronized cells have cilia if we assume only 20-60% of NIH3T3 cells have detectable circadian regulation of primary cilia. The authors should state this. In addition, the authors should consider live-cell imaging of cilia length for at least 48 h so that we can see the dynamics they infer from fixed and pooled samples.

> We agree with the reviewer's comment. Some population of cells show circadian rhythm of primary cilia. We stated this in the discussion (p. 20, line 5-17).

We have also performed live-cell imaging for 72 hours using NIH/3T3 cells that express Arl13b-venus endogenously and physiologically (Nakazato et al., *Meth. Cell. Biol.*, 175:45-68, 2023). The data show that individual primary cilia exhibited rhythmic elongation/shortening at ~24-hr cycle (Figure EV1B and Movie EV3).

3. Conflating cilia length with ciliation status - changes in the two are functionally different. This mistake is exemplified in the Kif3a KO experiments where they model disrupted circadian rhythm in cilia length using a total loss of cilia, which produces many consequences other than an isolated effect on circadian cilia length.

> We appreciate this critical comment. We thus generated Bbs2-deficient (Bbs2-KO) cells using CRISPR/Cas9-mediated NHEJ (Ijaz, Nakazato et al., *Sci. Rep.*, 12:11681, 2022) to have short cilia cells. Bbs2-KO NIH/3T3 cells did not completely lose primary cilia, but had very short primary cilia (Fig. EV3A). Bbs2-KO cells showed almost the same results as Kif3a-KO cells, i.e. Bbs2-KO cells lost rhythmic behaviors of wound healing as did Kif3a-KO cells (Fig. EV3B and C).

4. Conflating correlation with causation - comparing wound healing differences in cells at circadian time 30 h ("short cilia stage") vs. 18 or 42 h ("long cilia stage") does not prove causality between cilia length and wound healing. It's akin to saying if I'm asleep in the night and the sky also happens to be dark at night, then my sleep-wake cycle is controlling the sky. If there is to be a mechanistic link, then how might the authors propose that cilia are actually regulating cell migration? This key question remains unanswered. Signaling pathways were alluded - including cilia-mediated cell sensitivity to mechanical stimuli, which is plausible - but no experimental test for these hypotheses.

> We also appreciate this critical comment. We agree with the reviewer's concern. To address this issue, we generated two conditions where cells have normal length cilia or long cilia. As known well, forced expression of Arl13b causes elongation of primary cilia. We used cells that express Arl13b-venus in a forced manner as the long cilia model (Fig. EV4A). A normal cilia model is generated with physiological expression of Arl13b-venus under the endogenous Arl13b promoter (Fig. EV4A). Long-cilia cells showed slower healing after

wound (Fig. EV4B and EV4C). This supports the idea that cells with long cilia migrate more slowly than cell with short cilia in Fig. 8 (in the initial submission, Fig. 6).

5. The authors use a non-specific agonist of Reverba (SR9011) to test for a causal link from the circadian clock to cilia length. This Rev-erb agonist suppresses the core clock as well as affects many REVERBa-target genes. It is unclear if the drug directly affects circadian clock genes, cell migration, or primary cilia growth. The authors rely on 2 vs 10 uM SR9011 to segregate cilia elongation and inhibit circadian dynamics of cilia without careful controls to show dose dependence.

> We tested our hypothesis with a genetical approach in addition to the pharmacological approach. We newly generated Bmal1-KO cells using CRISPR/Cas9-mediated genome editing (Ijaz, Nakazato et al., *Sci. Rep.*, 12:11681, 2022). Bmal1-KO NIH/3T3 cells lost the rhythmic oscillation of primary cilia lengths (Fig. 2H and 2I)

Minor points:

6. Quantitation of cilia number and length: analysis method not discussed. Are these manually counted and measured, or did the authors use established automated cilia quantification ImageJ plugins e.g. CiliaQ? What is the number of cells and cilia quantitated, how many hpf?

> We have added the information in the methods section (p.31, line 16-23).

7. Pericentrin localizing to satellites is not itself mechanistic support for circadian control of ciliation. What is the actual mechanism? Does PCNT have Reverb/Bmal binding sites in its promoter? Is this protein produced, transported, degraded in a circadian manner?

> We have added the information in the discussion section (p.23, line 8-13).

8. Do cilia length fluctuations actually represent the ciliary cycle of reabsorption and growth? Can they estimate the minimum and maximum growth and resorption rates that would be required to explain their changes in cilia length over the day? Are these rates consistent with known rates of assembly and disassembly?

> Circadian rhythm of primary cilium length are observed in NIH/3T3 cells with slow cell cycle progression due to serum starvation and in neurons, which are non-dividing cells. Therefore, the circadian rhythm of primary cilium length in this study would be different from the

general primary cilium assembly/disassembly cycle linked to cell cycle.

9. The authors state that this is independent of the cell cycle. What is the evidence for a cilia circadian cycle independent of the cell cycle, and what is its purpose?

> In a precise sense, there is no direct evidence is provided for that the circadian oscillation of primary cilium length is independent of the cell cycle. In in vitro experiments, we cultured cells under serum-starved conditions, where usually NIH/3T3 cells enter almost a quiescent phase or the cell cycle slow down to more than 48 hours. These support our interpretation that 24-h oscillation of cilia length is independent of cell cycle. More simply, circadian changes of primary cilium length are also observed in neurons, which completely cease cell proliferation and are in G0 phase perfectly.

10. The authors mention without citation that circadian rhythms in primary cilia length has been reported previously. I found several on Bioarxiv. It would be good to put this study into context with appropriate citations of peer-reviewed papers on circadian primary cilia.

> We have added appropriate references in the introduction section (p.5, line 10-12).

Referee #2:

In this manuscript, Nakazato et. al. elucidated the regulation of ciliogenesis and cilium length by circadian rhythm. They showed that number and length of primary cilia in murine fibroblast cells show similar dynamics with rhythmic oscillations of clock genes. They also demonstrate similar oscillation in primary cilia of neurons, and astrocytes of the murine brain in vivo. Moreover, they investigated the mechanism by which the clock regulates primary cilium length by focusing on centriolar satellite protein Pericentrin and showed that its distribution in cells also changes during rhythmic clock oscillations. Finally, they used wound healing assays to show that cilium length changes contribute to the migration defects.

This manuscript is of general interest due to its focus on dissecting new relationship between circadian rhythm and primary cilia regulation, two important biological processes deregulated in disease. The relationship between circadian rhythm and primary cilium regulation has not been studies before, which is the novel and exciting part of the manuscript. The findings from the first two

figures are therefore very important and opens new areas of investigation. However, the characterization of the mechanisms that underlie ciliary length changes and their functional relevance is premature and the data presented for these parts are not solid and quantatitative (i.e. centriolar satelite phenotypes). Moreover, the conclusions reached for certain phenotypes were based on experiments that disrupt many cellular events (i.e. microtubule depolymerization, SR9011 treatment). These experiments suffer from specificity. Finally, the manuscript in most parts, particularly in introduction, does not have the proper references for the concepts introduced and discussed. Manuscript text has to be revised carefully to improve clarity in introduction, results and conclusions. Below are some of my major and minor comments:

> We appreciate the reviewer for the constructive and encouraging comments, by which our work have gotten improved greatly.

1- Although the authors only quantified cilium formation efficiency and length in functional assays, they refer to their analysis as investigation of "cilium morphology", which is not right. Morphology of cilia can only be assessed by imaging approaches with higher resolution (i.e. SEM, expansion microscopy).

> We agree with this comment and have avoided the use of the "morphology".

2- Fig.1: The quantification of the clock oscillations after DEX induction was performed over 96 h. To determine whether the ciliary oscillations of biogenesis and length correlates with clock oscillation, % of ciliated cells and cilium length can be quantified over 96 h instead of 48 h.

> Ninety six-hour culture of NIH/3T3 cells under the serum-starved condition is too harsh for the cells to be healthy. The maximal culture time under the serum-starved condition is 72 hours. As shown in the response to Referee #1's comment, we have extended the time to measure the primary cilia length over 72 hours (Fig. 1E, EV1A and EV1C).

3- Fig. 4A-C: The major mechanism the authors focused on for explaining the ciliary changes during circadian oscillations are centriolar satellite distribution and number. However, they only analyzed this angle by focusing on pericentrin, a protein that localizes both to the centrosome and cilia (Fig. 4). To convincingly show that centriolar satellites in part underlie the ciliary changes after DEX induction, centriolar satellites should be marked by their scaffolding protein PCM1 and their number, fluorescent intensity and cellular distribution should be quantified with better quantitative approaches than the ones used for Pericentrin (i.e. Galati, DF et al. Dev Cell 2018). Similar

quantitative analysis should also be performed for pericentrin, with an adaptation to remove the centrosomal signal of pericentirn.

> As to the quantification, we quantified PCNT localization according to the work by Galati et al. (Fig. 4D). The increase in PCNT at 30 hr after DEX release was reproduced in the better quantification (Fig. 4D).

We have also added data about localization of PCM1 in NIH/3T3 cells at each time points after synchronization in Fig. 4E and 4F. Surprisingly, PCM1 did not show clock-dependent oscillation. This unexpected results are discussed in p. 22, line 22-24; p. 23, line 1-13.

4- Fig. 4D-J: Colchicine treatment not only disrupts centriolar satellite positioning, but also affects other cellular structures transported by microtubules and function during cilium biogenesis. Therefore, this experiment is not sufficient to conclude that satellite positioning and thereby transport underlie the ciliary changes upon DEX induction. The method used by Aydin et al. 2021 to misposition satellites to the cell periphery can be used to specifically test the role of satellites in ciliary regulation during circadian oscillations.

> We cordially appreciate the reviewer's suggestion, and thoroughly agree with this comment. We thus tried manipulating PCNT localization with chemical dimerizing techniques. We used GFP-PCM1-FKBP, HA-Kif5b-FRB, and HA-BICD2-FRB for chemical dimerization (Fig. 5H). We succeeded in manipulating PCNT localization by controlling PCM1 localization with chemical dimerization; the satellite-like or cloud-like localization of PCNT at 30 hr after DEX release was perturbed both in Kif5b-FRB and BICD2-FRB expression with rapamycin treatment (Fig. 5J). The perturbation of satellite-like distribution of PCNT resulted in the shortening of primary cilia 12 hours later (i.e. 42 hours after DEX release) (Fig. 5K).

5- To uncover the mechanism by which cilium length is regulated after DEX induction, other key regulators of cilium length such as basal body/cilium recruitment of IFT proteins should be assessed after DEX induction. Microscopy-based analysis of centrosomal or ciliary levels of ciliogenesis factors at different time points after DEX induction will strengthen the conclusions of the manuscript on the link between clock and cilium length regulation.

> We have added data of IFT88 localization in primary cilia. In

DEX-synchronized cells, IFT88 showed oscillatory intra-cilia accumulation with the peak at 40 hours after DEX release (Fig. EV2B). In contrast, IFT88 amount at the base of primary cilia did not show circadian rhythms (Fig. EV2C). This results are discussed in p.22, line 16-17.

6- In addition to cell migration, ciliary functions such as Hedgehog signaling can also be checked to determine the physiological relevance of length-clock relationship and will complement the manuscript better.

> We appreciate this comment. We have measured the expression of *Gli1* and *Ptch1*, which are expressed downstream of the Hedgehog signaling pathway, in NIH/3T3 exposed to SAG by qPCR. Both genes expression responded to SAG better when cells have shorter cilia (28-32 hr after DEX release) (Fig. 6B).

7- Fig. 6: What were the parameters used for defining cells facing the wound and cells invading the wound area?

> We have added the information in the methods section (p. 31, line 12-14).

Minor:

References and text should be revised carefully to include the important references for the concepts included in the manuscript and to improve clarity. I listed below some of examples to this comment:

1- Reference is missing for the following sentence in introduction:

"For example, the autonomous cellular clock regulates fibroblast migration by controlling actin dynamics."

> We have added the references in the revised manuscript (p.4, line 4).

2- Revise the following sentence in introduction: "Defects in primary cilia due to congenital factors..."

> We have corrected the noted point and added the references in the revised manuscript (p.4, line 18-20).

3- "static" should be removed or replaced by "motile" in the following sentence: "Primary cilia are immotile, but not static."

> We agree with this comment and have removed the noted point in the revised manuscript.

4- Detailed introduction to ciliary decapitation and signaling can be removed from the introduction as the major focus of the manuscript is cilium assembly and length control.

> We agree with this comment and have removed the section in the revised manuscript.

5- The following sentence is not clear:

"Numerous studies on the effects of loss or mutation of primary cilia on cellular function in ciliopathy have revealed much regarding the importance of the "presence" of primary cilia."

> We have removed the noted point in the revised manuscript.

6- The paragraph about cilium length and morphology control should be revised to introduce the state-of-art. There are several comprehensive reviews on cilium length control and its physiological relevance (i.e. Broekhuis, JR. 2013, Keeling, J. 2016). These should be cited and included in the introduction.

> We have added these references and correct the noted point in the introduction section (p.5, line 1-4).

7- There are several important papers that uncovered the relationship between pericentrin, centriolar satellites and primary cilium (i.e. Chad Pearson's lab). They should be cited in the results part for Fig. 4 or even better in the introduction.

> We have added the references from Chad Pearson's lab in the revised manuscript.

Referee #3:

Review of "Circadian oscillation in primary cilium length by clock genes regulate fibroblast cell migration"

- Ryota Nakazato, Yuki Matsuda, Koji Ikegami

The authors of this manuscript provide evidence that a cell's cilia length is under control of the

circadian clockwork, and that this control influences cell motility using a combination of cell-culture and in vivo approaches. This is an interesting and novel area - circadian control of cilia/cell motility in mammalian cells. The authors data generally support their overall conclusions, but could use several modifications, clarifications and analyses, as detailed below.

> We appreciate the reviewer for the constructive and encouraging comments, by which our work have gotten improved greatly.

1. To say this is regulated by clock "genes" is substantially overstated. The authors did not directly disrupt clock genes, the only clock perturbation used is drug that may have unknown effects outside of modulating RevErb□ activity. However, they can simply delete "by clock genes" from the title and throughout without diminishing anything.

> We have generated Bmal1-KO cells (Fig. 2H and 2I). We have already indicated our response to this comment in our response to Referee #1.

2. Cilia length - how did they account for cilia projecting in different directions in 3D space? Is it possible that cilia lengths are sometimes in the z-axis? This would make them 'appear' shorter in a 2D confocal image (i.e. Fig 3D for example). This is an important potential caveat the authors should at least discuss. The differences observed in "length" could potentially be due to synchronized location/orientation instead of actual size.

> The primary cilia in NIH/3T3 cells grow parallel to the glass surface. The length of primary cilia in brain tissue is measured in three dimensions on the XYZ axis. Therefore, the difference in primary cilia length is not due to orientation. We have explained these carefully and in detail in the methods section (p. 31, line 17-23).

3. Statistics throughout the manuscript are inadequate. The authors use T-tests in place of ANOVAs (and Two-Way ANOVAs) throughout - this must be fixed for the sake of demonstrating experimental rigor. Similarly, the "n" used is often confusing - they state "3 experiments" but the measure is "number of cells" - n should be "number of cells".

> We have performed the statistics for all data using One-way ANOVA. We have also changed the notation to "three individual experiments" instead of using "n" for the number of cells or cilia only.

4. Several of their images are impossible to see what the authors are describing. Fig 1C and 6C are particularly bad - I cannot see even blown on my screen the differences reported.

> We have replaced Fig. 1C and Fig. 8C (in the initial submission, Fig. 6C) by the appropriate images.

1st Revision - Editorial Decision **18th Aug 2023**

Dear Dr. Nakazato,

Thank you for submitting your revised manuscript to EMBO reports and for your patience during its peer review. I apologize for the delayed response, which was due to the unavailability of the referees during the summer vacation period, and a backlog in our editorial offices. We have now received the full set of reports from the three referees that were asked to re-evaluate your study. Please find their comments included below.

As you will see, the referees acknowledge that the revised manuscript is substantially improved and that most of their previous concerns have been successfully addressed. However, referees #1 and #2 point out a few remaining concerns that remain to be addressed before we can proceed with acceptance of your manuscript for publication in EMBO reports. Please address all referees' comments in a revised version of your manuscript (make sure that all changes are highlighted or "tracked" to be clearly visible) and in a point-by-point response describing in detail any changes you might make in the revised manuscript.

From the editorial side, there are also a few things that we need from you before we can proceed with further handling of your manuscript:

- Please make sure that the title and the abstract of the revised manuscript are brief, yet explicit, even to non-specialists. The length of the title should not exceed 100 characters (including spaces), and the abstract should be a single paragraph not exceeding 175 words.

- Please provide up to 5 keywords in your revised manuscript (after the Abstract).

- The author contributions statement should be removed from the manuscript file. Instead, we now use CRediT to specify the contributions of each author in the journal submission system. Please use the free text box to provide more detailed descriptions. See also our guide to authors:
<https://www.embopress.org/page/journal/14693178/authorguide#authorshipguidelines>.

- The heading of your conflict-of-interest statement should be updated to "Disclosure and competing interests statement".

- Your Figure legends have been inspected by our data editors for completeness and accuracy. Please see the required changes in the attached Word file and address all comments in your revised manuscript (with tracked changes).

- Please upload each one of your movies in an individual ZIP folder also containing a text file with the respective movie legend.

- We noticed that Movie EV3 is in two parts. Please consider making one of them Movie EV4. In that case, please update the callouts accordingly.

- Please note that EMBO press papers are accompanied online by:
A) a short (1-2 sentences) summary of the findings and their significance,
B) 2-4 short bullet points highlighting the key results, and
C) a synopsis image that is exactly 550 pixels wide and 300-600 pixels high (the height is variable). You can either show a model or key data in the synopsis image. Please note that the text needs to be readable at the final size.
Please upload this information along with your revised manuscript (the text for A and B should be provided in a separate Word file).

Please also note that as part of the EMBO publications' Transparent Editorial Process, EMBO reports publishes online a Review Process File to accompany accepted manuscripts. This File will be published in conjunction with your paper and will include the referee reports, your point-by-point response and all pertinent correspondence relating to the manuscript.

You can opt out of this by letting the editorial office know (emboreports@embo.org). If you do opt out, the Review Process File link will point to the following statement: "No Review Process File is available with this article, as the authors have chosen not to make the review process public in this case."

We would also welcome the submission of cover suggestions or motifs to be used by our Graphics Illustrator in designing a cover.

We look forward to seeing a final version of your manuscript as soon as possible.

Yours sincerely,

Ioannis Papaioannou, PhD
Editor
EMBO reports

Referee #1:

The authors have added new data and text to address the concerns from the prior three reviewers. They are applauded for independent tests of circadian rhythmicity in primary cilia length in NIH3T3 cells: 48h sampling from cultured cells, 72-h imaging of single cilia, and addition of Bmal1 knockout cells. The authors also cite a recent publication that found similar daily rhythms in cilia length in neurons of the suprachiasmatic nucleus (Tu et al., 2023, Science). This reviewer is impressed that there may be a circadian rhythm in NIH3T3 cells and not astrocytes, as claimed by the authors, but not fully convinced.

Figures 1E. These extra 24-h of data in Figure1 strengthen the manuscript, but require further explanation. The authors remain married to the conclusion that their NIH3T3 cells have circadian rhythms with limited support. They should acknowledge that the small variability in cilia length of the vehicle-treated cells (Fig. 1E) is also consistent with all cells having no daily rhythm in those dishes. If there were more variability in the controls, we could conclude that DEX synchronizes their daily rhythms. Instead, we now have more evidence that DEX induces a program that changes cilia length, but I remain concerned this is not under circadian control.

Figure EV1. The new data in Figure EV1 are too preliminary to include in this manuscript without further replication and better image quality. The movie is so noisy that I cannot see the daily rhythm of a single cilium they claim to track for 72 hours in Fig. EV1B. It is unclear how they could accurately measure length from the highly pixelated cilium which appears to move in and out of focus at irregular intervals. Minimally, the authors should add time to the images in the movie and revise their Discussion. The authors claim to consider that some cells are not circadian in their Discussion on P. 20, lines 5-17, but they really need to state that their "fraction of ciliated cells data are consistent with only 20-60% of NIH3T3 cells having detectable circadian regulation of primary cilia."

Referee #2:

In this manuscript, Nakazato et. al. elucidated the regulation of ciliogenesis and cilium length by circadian rhythm. They showed that the number and length of primary cilia in murine fibroblast cells show similar dynamics with rhythmic oscillations of clock genes. I agree with the authors that the reviewer experiments they performed for the revised manuscript significantly improved the manuscript. I thank the authors for all the effort they put into the revision process. Although most of my comments were addressed, the following points that pertain to centriolar satellite-based mechanisms and quantifications need to be addressed to support the major conclusions of the paper.

1- Fig.1: The quantification of the clock oscillations after DEX induction was performed over 96 h. To determine whether the ciliary oscillations of biogenesis and length correlates with clock oscillation, % of ciliated cells and cilium length can be quantified over 96 h instead of 48 h.
The authors partially addressed this comment. Even though I agree with their point on 96 h being too harsh to serum starve the cells, they only quantified cilium length over 72 h. As I indicated previously, they should also quantify the percentage of ciliated cells over 72 h, which is important to support their model on ciliogenesis and clock oscillations (Fig. 1D)

2- Why do authors discuss the PACT domain and calmodulin interaction to explain the different phenotype exhibited by PCM1 and pericentrin? PACT domain is responsible for centrosomal targeting of pericentrin, and does not contribute to its centriolar satellite localization.

3- Fig. 5A: Better representative images for control cells are required to indicate intact microtubule cytoskeleton.

4- Fig. 5J: Pericentrin does not colocalize with GFP-PCM1 puncta in control and Kif5b-FRB cells. How did the authors conclude that pericentrin redistributes with pericentrin based on this data? For example, pericentrin accumulates in a big aggregate in Kif-5b case whereas PCM1-positive satellites are at the periphery. If this is the case, it is not possible to conclude that pericentrin repositioning results in cilium length reduction.

5- The following sentence should be revised as the cited paper reported functions for centriolar satellites during primary cilium maintenance.

The centriolar satellites are reported to be required for the process of primary cilium formation, but do not contribute much to the stability of primary cilia (Aydin et al., 2020).

6- The following sub-paragraph should be revised as the message that is conveyed through this discussion is ambiguous. Although it is not reported how long it takes for the primary cilia to reach their maximum length from the formation of centriolar satellites, a time lag of about 12 h, as in the results of this study, is expected to exist. Indeed, the increase in primary cilia of IFT88 in the present study reached a maximum at 40 h after synchronization.

Referee #3:

The authors were thorough in addressing any and all concerns from my previous review, as well as the other reviewers. Everything looks good.

Referee #1:

The authors have added new data and text to address the concerns from the prior three reviewers. They are applauded for independent tests of circadian rhythmicity in primary cilia length in NIH3T3 cells: 48h sampling from cultured cells, 72-h imaging of single cilia, and addition of Bmal1 knockout cells. The authors also cite a recent publication that found similar daily rhythms in cilia length in neurons of the suprachiasmatic nucleus (Tu et al., 2023, Science). This reviewer is impressed that there may be a circadian rhythm in NIH3T3 cells and not astrocytes, as claimed by the authors, but not fully convinced.

> We appreciate the reviewer for the encouraging comments.

Figures 1E. These extra 24-h of data in Figure1 strengthen the manuscript, but require further explanation. The authors remain married to the conclusion that their NIH3T3 cells have circadian rhythms with limited support. They should acknowledge that the small variability in cilia length of the vehicle-treated cells (Fig. 1E) is also consistent with all cells having no daily rhythm in those dishes. If there were more variability in the controls, we could conclude that DEX synchronizes their daily rhythms. Instead, we now have more evidence that DEX induces a program that changes cilia length, but I remain concerned this is not under circadian control.

> The error bars shown in Figure 1E indicate variation among three means of each independent experiments and do not indicate variation in the length of primary cilia among individual cells. Therefore, the variation in the data of vehicle-treated cells is small.

Figure EV1. The new data in Figure EV1 are too preliminary to include in this manuscript without further replication and better image quality. The movie is so noisy that I cannot see the daily rhythm of a single cilium they claim to track for 72 hours in Fig. EV1B. It is unclear how they could accurately measure length from the highly pixelated cilium which appears to move in and out of focus at irregular intervals. Minimally, the authors should add time to the images in the movie and revise their Discussion. The authors claim to consider that some cells are not circadian in their Discussion on P. 20, lines 5-17, but they really need to state that their "fraction of ciliated cells data are consistent with only 20-60% of NIH3T3 cells having detectable circadian regulation of primary cilia."

> The pixel size of movies is 0.32 µm. Since the difference between the

maximum and minimum primary cilium lengths is more than 10 pixels, we consider the spatial resolution of movies to be enough to measure the change of primary cilium length. Defocusing seems to be quite limited at least for our eyes. Primary cilia were placed in the focal plane for 72 hours. These were achieved by using LCV110, which is embedded in CO2 incubator, and a non-high-NA dry lens, which provides large depth of field (DOF). We added these information to the Methods (p. 30, line 20-23).

We have decreased the background noises of movies by manipulating image contrasts, inverted the color for cilia to be more visible, and added time stamps to movies (Figure EV1A; Movie EV3 and EV4). We also stated the noted point in the discussion (p. 20, line 9-11).

Referee #2:

In this manuscript, Nakazato et. al. elucidated the regulation of ciliogenesis and cilium length by circadian rhythm. They showed that the number and length of primary cilia in murine fibroblast cells show similar dynamics with rhythmic oscillations of clock genes. I agree with the authors that the reviewer experiments they performed for the revised manuscript significantly improved the manuscript. I thank the authors for all the effort they put into the revision process. Although most of my comments were addressed, the following points that pertain to centriolar satellite-based mechanisms and quantifications need to be addressed to support the major conclusions of the paper.

> We appreciate the reviewer for the encouraging comments.

1- Fig.1: The quantification of the clock oscillations after DEX induction was performed over 96 h. To determine whether the ciliary oscillations of biogenesis and length correlates with clock oscillation, % of ciliated cells and cilium length can be quantified over 96 h instead of 48 h.
The authors partially addressed this comment. Even though I agree with their point on 96 h being too harsh to serum starve the cells, they only quantified cilium length over 72 h. As I indicated previously, they should also quantify the percentage of

ciliated cells over 72 h, which is important to support their model on ciliogenesis and clock oscillations (Fig. 1D)

> We have measured and quantified the percentage of ciliated cells over 72 h. We have replaced Fig. 1C and 1D with the new one, and removed data of Fig. EV1A.

2- Why do authors discuss the PACT domain and calmodulin interaction to explain the different phenotype exhibited by PCM1 and pericentrin? PACT domain is responsible for centrosomal targeting of pericentrin, and does not contribute to its centriolar satellite localization.

> We agree with this comment and have removed the noted point in the revised manuscript.

3- Fig. 5A: Better representative images for control cells are required to indicate intact microtubule cytoskeleton.

> We have replaced Fig. 5A by the better images.

4- Fig. 5J: Pericentrin does not colocalize with GFP-PCM1 puncta in control and Kif5b-FRB cells. How did the authors conclude that pericentrin redistributes with pericentrin based on this data? For example, pericentrin accumulates in a big aggregate in Kif-5b case whereas PCM1-positive satellites are at the periphery. If this is the case, it is not possible to conclude that pericentrin repositioning results in cilium length reduction.

> We appreciate this comment. PCM1 puncta and PCNT puncta are not always considered to be in the same place. Indeed, it has been reported that the merge ratio of PCM1 puncta and PCNT puncta is 48% (Kubo et al., 2002). PCM1 is required for PCNT to be present in the centriolar satellites, but PCNT is not necessarily present where PCM1 is present. Thus, PCNT puncta is not likely to be present in the same ectopic PCM1 puncta locations found in Kif5b-FRB-expressing cells. We have added these explanation in the discussion (p. 23, line 13-17).

5- The following sentence should be revised as the cited paper reported functions for centriolar satellites during primary cilium maintenance.

The centriolar satellites are reported to be required for the process of primary cilium

formation, but do not contribute much to the stability of primary cilia (Aydin et al., 2020).

> We agree with this comment and have removed the noted point in the revised manuscript.

6- The following sub-paragraph should be revised as the message that is conveyed through this discussion is ambiguous.

Although it is not reported how long it takes for the primary cilia to reach their maximum length from the formation of centriolar satellites, a time lag of about 12 h, as in the results of this study, is expected to exist. Indeed, the increase in primary cilia of IFT88 in the present study reached a maximum at 40 h after synchronization.

> We agree with this comment and have revised the noted point in the revised manuscript (p.22, line 17-22).

Referee #3:

The authors were thorough in addressing any and all concerns from my previous review,

> We appreciate the reviewer for the encouraging comments.

2nd Revision - Editorial Decision 20th Oct 2023

Dear Dr. Nakazato,

Thank you for submitting your revised manuscript. Since my colleague Ioannis has moved over to The EMBO Journal, I have stepped in as the handling editor of your manuscript. I have now looked at everything and all is fine. Therefore, I am very pleased to accept your manuscript for publication in EMBO Reports.

Congratulations on a nice work!

Kind regards,

Deniz Senyilmaz Tiebe
--
Deniz Senyilmaz Tiebe, PhD
Scientific Editor
EMBO Reports

--

At the end of this email I include important information about how to proceed. Please ensure that you take the time to read the information and complete and return the necessary forms to allow us to publish your manuscript as quickly as possible.

As part of the EMBO publication's Transparent Editorial Process, EMBO reports publishes online a Review Process File to accompany accepted manuscripts. As you are aware, this File will be published in conjunction with your paper and will include the referee reports, your point-by-point response and all pertinent correspondence relating to the manuscript.

If you do NOT want this File to be published, please inform the editorial office within 2 days, if you have not done so already, otherwise the File will be published by default [contact: emboreports@embo.org]. If you do opt out, the Review Process File link will point to the following statement: "No Review Process File is available with this article, as the authors have chosen not to make the review process public in this case."

Thank you again for your contribution to EMBO reports and congratulations on a successful publication. Please consider us again in the future for your most exciting work.

****

THINGS TO DO NOW:

Please note that you will be contacted by Wiley Author Services to complete licensing and payment information. The required 'Page Charges Authorization Form' is available here: https://www.embopress.org/pb-assets/embo-site/er_apc.pdf - please download and complete the form and return to embopressproduction@wiley.com

EMBO Press participates in many Publish and Read agreements that allow authors to publish Open Access with reduced/no publication charges. Check your eligibility: https://authorservices.wiley.com/author-resources/Journal-Authors/open-access/affiliation-policies-payments/index.html

You will receive proofs by e-mail approximately 2-3 weeks after all relevant files have been sent to our Production Office; you should return your corrections within 2 days of receiving the proofs.

Please inform us if there is likely to be any difficulty in reaching you at the above address at that time. Failure to meet our deadlines may result in a delay of publication, or publication without your corrections.

All further communications concerning your paper should quote reference number EMBOR-2023-56870V3 and be addressed to emboreports@wiley.com.

Should you be planning a Press Release on your article, please get in contact with emboreports@wiley.com as early as possible, in order to coordinate publication and release dates.

EMBO Press Author Checklist

Corresponding Author Name: Ryota Nakazato

Journal Submitted to: EMBO Reports
Manuscript Number: EMBOR-2023-56870V1

USEFUL LINKS FOR COMPLETING THIS FORM
The EMBO Journal - Author Guidelines
EMBO Reports - Author Guidelines
Molecular Systems Biology - Author Guidelines
EMBO Molecular Medicine - Author Guidelines

Reporting Checklist for Life Science Articles (updated January
This checklist is adapted from Materials Design Analysis Reporting (MDAR) Checklist for Authors. MDAR establishes a minimum set of requirements in transparent reporting in the life sciences (see Statement of Task: 10.31222/osf.io/9sm4x). Please follow the journal's guidelines in preparing your
Please note that a copy of this checklist will be published alongside your article.

Abridged guidelines for figures
1. Data
The data shown in figures should satisfy the following conditions:
- the data were obtained and processed according to the field's best practice and are presented to reflect the results of the experiments in an accurate and unbiased manner.
- ideally, figure panels should include only measurements that are directly comparable to each other and obtained with the same assay.
- plots include clearly labeled error bars for independent experiments and sample sizes. Unless justified, error bars should not be shown for technical
- if n<5, the individual data points from each experiment should be plotted. Any statistical test employed should be justified.
- Source Data should be included to report the data underlying figures according to the guidelines set out in the authorship guidelines on Data

2. Captions
Each figure caption should contain the following information, for each panel where they are relevant:
- a specification of the experimental system investigated (eg cell line, species name).
- the assay(s) and method(s) used to carry out the reported observations and measurements.
- an explicit mention of the biological and chemical entity(ies) that are being measured.
- an explicit mention of the biological and chemical entity(ies) that are altered/varied/perturbed in a controlled manner.
- the exact sample size (n) for each experimental group/condition, given as a number, not a range;
- a description of the sample collection allowing the reader to understand whether the samples represent technical or biological replicates (including how many animals, litters, cultures, etc.).
- a statement of how many times the experiment shown was independently replicated in the laboratory.
- definitions of statistical methods and measures:
  - common tests, such as t-test (please specify whether paired vs. unpaired), simple χ2 tests, Wilcoxon and Mann-Whitney tests, can be unambiguously identified by name only, but more complex techniques should be described in the methods section;
  - are tests one-sided or two-sided?
  - are there adjustments for multiple comparisons?
  - exact statistical test results, e.g., P values = x but not P values < x;
  - definition of 'center values' as median or average;
  - definition of error bars as s.d. or s.e.m.

Please complete ALL of the questions below.
Select "Not Applicable" only when the requested information is not relevant for your study.

Materials

Newly Created Materials	Information included in the manuscript?	In which section is the information available? (Reagents and Tools Table, Materials and Methods, Figures, Data Availability Section)
New materials and reagents need to be available; do any restrictions apply?	Not Applicable	

Antibodies	Information included in the manuscript?	In which section is the information available? (Reagents and Tools Table, Materials and Methods, Figures, Data Availability Section)
For **antibodies** provide the following information:<br>- Commercial antibodies: RRID (if possible) or supplier name, catalogue number and or/clone number<br>- Non-commercial: RRID or citation	Yes	Materials and Methods

DNA and RNA sequences	Information included in the manuscript?	In which section is the information available? (Reagents and Tools Table, Materials and Methods, Figures, Data Availability Section)
**Short novel DNA or RNA including primers, probes**: provide the sequences.	Yes	Materials and Methods

Cell materials	Information included in the manuscript?	In which section is the information available? (Reagents and Tools Table, Materials and Methods, Figures, Data Availability Section)
**Cell lines:** Provide species information, strain. Provide accession number in repository **OR** supplier name, catalog number, clone number, and/**OR** RRID.	Yes	Materials and Methods
**Primary cultures:** Provide species, strain, sex of origin, genetic modification status.	Not Applicable	
Report if the cell lines were recently **authenticated** (e.g., by STR profiling) and tested for mycoplasma contamination.	Not Applicable	

Experimental animals	Information included in the manuscript?	In which section is the information available? (Reagents and Tools Table, Materials and Methods, Figures, Data Availability Section)
**Laboratory animals or Model organisms:** Provide species, strain, sex, age, genetic modification status. Provide accession number in repository **OR** supplier name, catalog number, clone number, **OR** RRID.	Yes	Materials and Methods
**Animal observed in or captured from the field:** Provide species, sex, and age where possible.	Yes	Materials and Methods
Please detail **housing and husbandry conditions**.	Yes	Materials and Methods

Plants and microbes	Information included in the manuscript?	In which section is the information available? (Reagents and Tools Table, Materials and Methods, Figures, Data Availability Section)
**Plants:** provide species and strain, ecotype and cultivar where relevant, unique accession number if available, and source (including location for collected wild specimens).	Not Applicable	
**Microbes:** provide species and strain, unique accession number if available, and source.	Not Applicable	

Human research participants	Information included in the manuscript?	In which section is the information available? (Reagents and Tools Table, Materials and Methods, Figures, Data Availability Section)
If collected and within the bounds of privacy constraints report on age, sex and gender or ethnicity for all study participants.	Not Applicable	

Core facilities	Information included in the manuscript?	In which section is the information available? (Reagents and Tools Table, Materials and Methods, Figures, Data Availability Section)
If your work benefited from core facilities, was their service mentioned in the acknowledgments section?	Not Applicable	

Design

Study protocol	Information included in the manuscript?	In which section is the information available? (Reagents and Tools Table, Materials and Methods, Figures, Data Availability Section)

If study protocol has been **pre-registered, provide DOI in the manuscript**. For clinical trials, provide the trial registration number **OR** cite DOI.	Not Applicable	
Report the **clinical trial registration number** (at ClinicalTrials.gov or equivalent), where applicable.	Not Applicable	

Laboratory protocol	Information included in the manuscript?	In which section is the information available? (Reagents and Tools Table, Materials and Methods, Figures, Data Availability Section)
Provide DOI OR other citation details if **external detailed step-by-step protocols** are available.	Not Applicable	

Experimental study design and statistics	Information included in the manuscript?	In which section is the information available? (Reagents and Tools Table, Materials and Methods, Figures, Data Availability Section)
Include a statement about **sample size** estimate even if no statistical methods were used.	Yes	Figure legends
Were any steps taken to minimize the effects of subjective bias when allocating animals/samples to treatment (e.g. **randomization procedure**)? If yes, have they been described?	Yes	Materials and Methods
Include a statement about **blinding** even if no blinding was done.	Yes	Materials and Methods
Describe **inclusion/exclusion criteria** if samples or animals were excluded from the analysis. Were the criteria pre-established?<br><br>If sample or data points were omitted from analysis, report if this was due to attrition or intentional exclusion and provide justification.	Not Applicable	
For every figure, are **statistical tests** justified as appropriate? Do the data meet the assumptions of the tests (e.g., normal distribution)? Describe any methods used to assess it. Is there an estimate of variation within each group of data? Is the variance similar between the groups that are being statistically compared?	Yes	Materials and Methods and Figure legends

Sample definition and in-laboratory replication	Information included in the manuscript?	In which section is the information available? (Reagents and Tools Table, Materials and Methods, Figures, Data Availability Section)
In the figure legends: state number of times the experiment was **replicated** in laboratory.	Yes	
In the figure legends: define whether data describe **technical or biological replicates**.	Yes	

Ethics

Ethics	Information included in the manuscript?	In which section is the information available? (Reagents and Tools Table, Materials and Methods, Figures, Data Availability Section)
Studies involving **human participants**: State details of **authority granting ethics approval** (IRB or equivalent committee(s), provide reference number for approval.	Not Applicable	
Studies involving **human participants**: Include a statement confirming that **informed consent** was obtained from all subjects and that the experiments conformed to the principles set out in the WMA Declaration of Helsinki and the Department of Health and Human Services Belmont Report.	Not Applicable	
Studies involving **human participants:** For publication of **patient photos**, include a statement confirming that consent to publish was obtained.	Not Applicable	
Studies involving experimental **animals**: State details of **authority granting ethics approval** (IRB or equivalent committee(s), provide reference number for approval. Include a statement of compliance with ethical regulations.	Yes	Materials and Methods
Studies involving **specimen and field samples:** State if relevant **permits** obtained, provide details of authority approving study; if none were required, explain why.	Not Applicable	

Dual Use Research of Concern (DURC)	Information included in the manuscript?	In which section is the information available? (Reagents and Tools Table, Materials and Methods, Figures, Data Availability Section)
Could your study fall under dual use research restrictions? Please check biosecurity documents and list of **select agents and toxins** (CDC): https://www.selectagents.gov/sat/list.htm	Not Applicable	
If you used a select agent, is the security level of the lab appropriate and reported in the manuscript?	Not Applicable	
If a study is subject to dual use research of concern regulations, is the name of the **authority granting approval and reference number** for the regulatory approval provided in the manuscript?	Not Applicable	

Reporting

The MDAR framework recommends adoption of discipline-specific guidelines, established and endorsed through community initiatives. Journals have their own policy about requiring specific guidelines and recommendations to complement MDAR.

Adherence to community standards	Information included in the manuscript?	In which section is the information available? (Reagents and Tools Table, Materials and Methods, Figures, Data Availability Section)
State if relevant guidelines or checklists (e.g., **ICMJE, MIBBI, ARRIVE, PRISMA**) have been followed or provided.	Not Applicable	
For **tumor marker prognostic studies,** we recommend that you follow the **REMARK** reporting guidelines (see link list at top right). See author guidelines, under 'Reporting Guidelines'. Please confirm you have followed these guidelines.	Not Applicable	
For **phase II and III randomized controlled trials**, please refer to the **CONSORT** flow diagram (see link list at top right) and submit the CONSORT checklist (see link list at top right) with your submission. See author guidelines, under 'Reporting Guidelines'. Please confirm you have submitted this list.	Not Applicable	

Data Availability

Data availability	Information included in the manuscript?	In which section is the information available? (Reagents and Tools Table, Materials and Methods, Figures, Data Availability Section)
Have **primary datasets** been deposited according to the journal's guidelines (see 'Data Deposition' section) and the respective accession numbers provided in the Data Availability Section?	Not Applicable	
Were **human clinical and genomic datasets** deposited in a public access-controlled repository in accordance to ethical obligations to the patients and to the applicable consent agreement?	Not Applicable	
Are **computational models** that are central and integral to a study available without restrictions in a machine-readable form? Were the relevant accession numbers or links provided?	Not Applicable	
If publicly available data were reused, provide the respective **data citations in the reference list**.	Not Applicable	
